# Identification of five novel genetic loci related to facial morphology by genome-wide association studies

**DOI:** 10.1186/s12864-018-4865-9

**Published:** 2018-06-19

**Authors:** Seongwon Cha, Ji Eun Lim, Ah Yeon Park, Jun-Hyeong Do, Si Woo Lee, Chol Shin, Nam Han Cho, Ji-One Kang, Jeong Min Nam, Jong-Sik Kim, Kwang-Man Woo, Seung-Hwan Lee, Jong Yeol Kim, Bermseok Oh

**Affiliations:** 1 Future Medicine Division, Korea Institute of Oriental Medicine, Daejeon, 34054 Republic of Korea; 2 Department of Biochemistry and Molecular Biology, School of Medicine, Kyung Hee University, Seoul, 02447 Republic of Korea; 3 Mibyeong Research Center, Korea Institute of Oriental Medicine, Daejeon, 34054 Republic of Korea; 4 Division of Pulmonary Sleep and Critical Care Medicine, Department of Internal Medicine, Korea University Ansan Hospital and Institute of Human Genomic Study, Korea University Ansan Hospital, Ansan, 15355 Republic of Korea; 5 Department of Preventive Medicine, Ajou University School of Medicine, Suwon, 16499 Republic of Korea; 6 DNA Forensic Division, Supreme Prosecutors’ Office, Seoul, 06590 Republic of Korea; 7 KM Fundamental Research Division, Korea Institute of Oriental Medicine, Daejeon, 34054 Republic of Korea

**Keywords:** Face morphology, GWAS, Korean, *OSR1-WDR35*, *HOXD1-MTX2*, *WDR27*, *SOX9*, *DHX35*

## Abstract

**Background:**

Face morphology is strongly determined by genetic factors. However, only a small number of genes related to face morphology have been identified to date. Here, we performed a two-stage genome-wide association study (GWAS) of 85 face morphological traits in 7569 Koreans (5643 in the discovery set and 1926 in the replication set).

**Results:**

In this study, we analyzed 85 facial traits, including facial angles. After discovery GWAS, 128 single nucleotide polymorphisms (SNPs) showing an association of *P* < 5 × 10^− 6^ were selected to determine the replication of the associations, and meta-analysis of discovery GWAS and the replication analysis resulted in five genome-wide significant loci. The *OSR1-WDR35* [rs7567283, G allele, beta (se) = −0.536 (0.096), *P* = 2.75 × 10^− 8^] locus was associated with the facial frontal contour; the *HOXD1-MTX2* [rs970797, A allele, beta (se) = 0.015 (0.003), *P* = 3.97 × 10^− 9^] and *WDR27* [rs3736712, C allele, beta (se) = 0.293 (0.048), *P* = 8.44 × 10^− 10^] loci were associated with eye shape; and the *SOX9* [rs2193054, C allele, beta (se) (ln-transformed) = −0.007 (0.001), *P* = 6.17 × 10^− 17^] and *DHX35* [rs2206437, A allele, beta (se) = −0.283 (0.047), *P* = 1.61 × 10^− 9^] loci were associated with nose shape. *WDR35* and *SOX9* were related to known craniofacial malformations, i.e., cranioectodermal dysplasia 2 and campomelic dysplasia, respectively. In addition, we found three independent association signals in the *SOX9* locus, and six known loci for nose size and shape were replicated in this study population. Interestingly, four SNPs within these five face morphology-related loci showed discrepancies in allele frequencies among ethnic groups.

**Conclusions:**

We identified five novel face morphology loci that were associated with facial frontal contour, nose shape, and eye shape. Our findings provide useful genetic information for the determination of face morphology.

**Electronic supplementary material:**

The online version of this article (10.1186/s12864-018-4865-9) contains supplementary material, which is available to authorized users.

## Background

Face morphology is an important feature for both craniofacial clinics and forensic science. Clinically, craniofacial deformities comprise over half of all congenital malformations [1], and affected patients suffer not only from clinical syndromes but also social maladjustment [2, 3]. In forensic science, genetic information is considered to be important evidence, providing data regarding personal identification and externally visible characteristics such as iris and hair color [4, 5]. Recently, advanced high-resolution three-dimensional imaging techniques that use face morphology as a statistical value have opened up new possibilities of genetically aided facial modeling technology combined with more available DNA information [6, 7].

The heritability of craniofacial traits has been reported to be 0.8 based on analysis of the lateral cephalogram from X-ray profiles of parents and their offspring [8] or 0.41–0.86 based on twin studies [9], suggesting that face morphology is more strongly determined by genetic factors than environmental factors. The process of face formation is an evolutionarily conserved, precisely orchestrated process involving the cellular migration, interaction, proliferation, and differentiation of diverse tissue cells [10]. Studies using human subjects and animal models with congenital craniofacial malformations have explored the genetic factors affecting face formation. Consequently, multiple signaling pathways have been reported to be important during embryonic craniofacial morphogenesis, including bone morphogenic protein, sonic hedgehog, fibroblast growth factor, growth hormone receptor, and Wnt/β-catenin pathways [11].

Paternoster et al. [12] conducted a genome-wide association study (GWAS) and found that a genetic variant of the *PAX3* locus was associated with nose shape. Liu et al. [13] confirmed the association of the *PAX3* locus in European subjects, and further identified four novel genetic variants affecting face morphology close to the genes *PRDM16, TP63, C5orf50*, and *COL17A1*. More recently, Adhikari et al. [14] identified five more genetic variants related to nose shape close to the genes *DCHS2, RUNX2, GLI3, PAX1*, and *EDAR.* Shaffer et al. [15] identified seven additional genetic variants for face traits such as facial width and depth, and nose shape. Cole et al. [16] identified two additional genetic variants of *SCHIP1* and *PDE8A* associated with facial size, and Lee et al. [17] identified two additional genetic variants of *FREM1* and *PARK2* associated with face shape.

Despite these studies, our current understanding of face morphology falls far short of expectations in craniofacial medicine and forensic science. Therefore, the discovery of more genetic variants using a large-scale face morphology GWAS may contribute to identification of the etiology of human craniofacial malformations and externally visible characteristics. Therefore, in this study, we aimed to identify genetic factors associated with face morphology in a large cohort of 7569 samples from the Korean population (East Asians). To the best of our knowledge, this study represents the first GWAS on facial morphology in the Korean population.

## Results

### Characteristics of facial traits and their heritability

**Fig. 1:**
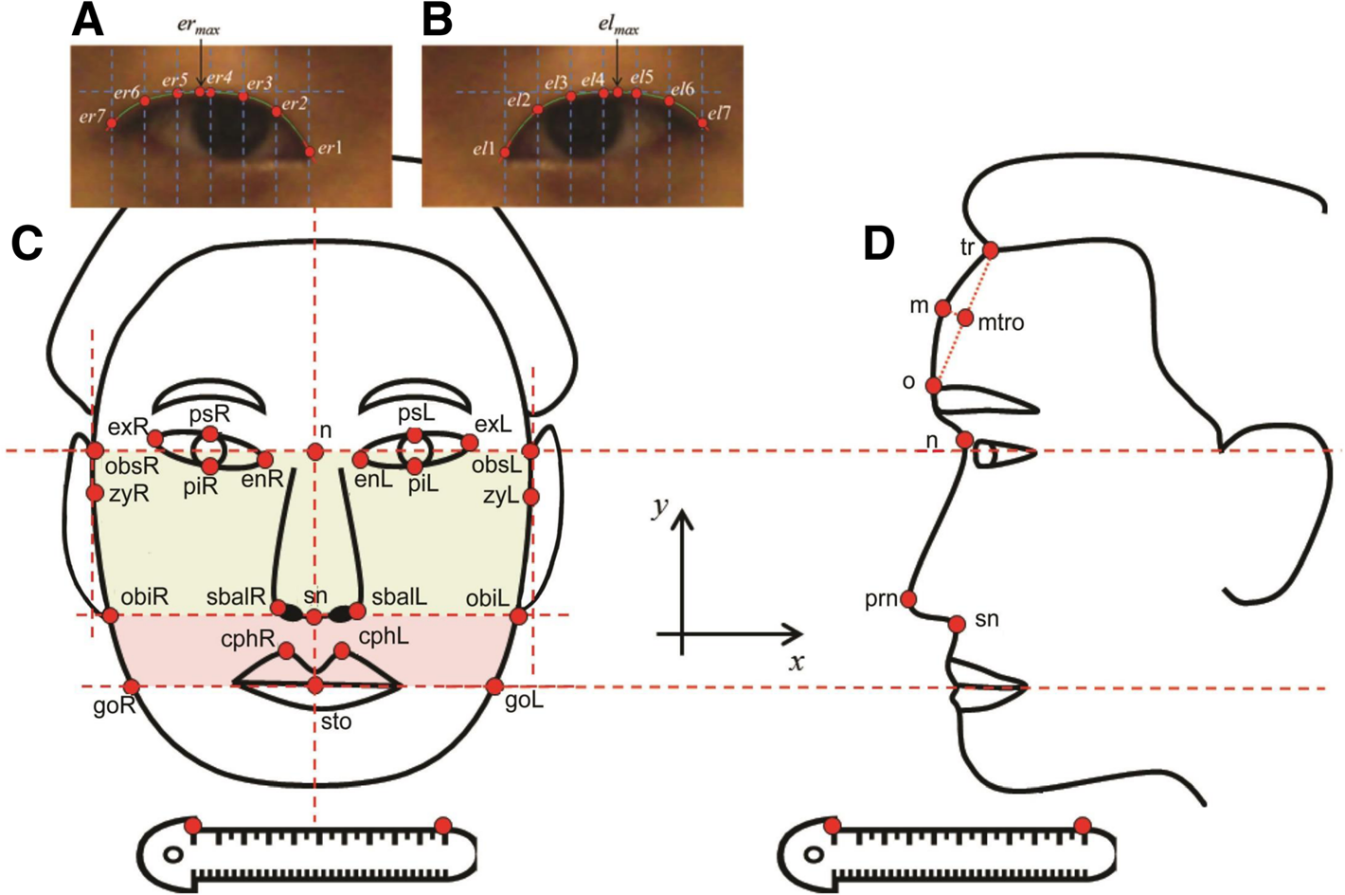
Facial points used for the facial traits. The participants were photographed in both frontal and profile views, and 23 frontal and seven lateral face points were extracted (modified from Fig. 1 of reference [34]). Facial phenotypes, such as distance, angle, and area, were measured based on in-house facial data acquisition software. **a** Points in the right eye, **b** points in the left eye, **c** points and areas in frontal images, **d** points in lateral images

For a comprehensive investigation of face morphology, a total of 85 facial parameters were selected from both frontal and lateral pictures of the individual subjects, including 17 frontal, 11 forehead, 13 eye, 32 upper eyelid, 11 nose, and two mouth features (Table 1). Facial traits were delineated by distance, distance ratio, angle, area, and curvature (eyelid) from 23 frontal and seven lateral face points, which were automatically extracted from each picture using in-house developed software (Fig. 1) [18].

**Table 1:** Eighty-five facial traits from 23 frontal, 7 profile, and 16 upper eyelid points

Size-related variables			Shape-related variables		
	Abbreviation	Description		Abbreviation	Description
Face shape	(17 traits)				
Width^a^	zyR-zyL	Facial base width	Ratio	zyR-zyL/goR -goL	Facial width ratio of base to chin
	goR -goL	Lower facial width		zyR-zyL/n-sto	Facial ratio of base width to height
	obsR-obsL	Upper facial width		goR -goL/n-sto	Facial ratio of chin width to height
	obiR-obiL	Middle facial width			
Height^b^	sn-sto	Upper lip height	Angle^c^	(A) enR-exR-goR	Right facial angle of en-ex-go
	n-sto	Facial height		(A) enL-exL-goL	Left facial angle of en-ex-go
Area	(AR) obsR-obsL-obiR-obiL	Upper facial area		(A) psR-exR-goR	Right facial angle of ps-ex-go
	(AR) obiR-obiL-goR -goL	Lower facial area		(A) psL-exL-goL	Left facial angle of ps-ex-go
				(A) enR-psR-goR	Right facial angle of en-ps-go
				(A) enL-psL-goL	Left facial angle of en-ps-go
Forehead	(10 traits)				
Height^b^	(V) tr-o	Forehead height	Angle^c^	(A) m-tr	Upper forehead slant angle
	(V) m-o	Lower forehead height		(A) o-n	Brow ridge protrusion angle
	(V) tr-m	Upper forehead height	Ratio	mtro-o/t-o	Metopion position ratio
	(V) o-n	Brow ridge height	Depth	(H) tr-m	Upper forehead slant depth
				(H) o-n	Brow ridge protrusion
				m-mtro	Metopion eminence depth
Eye	(15 traits)				
Width^a^	enR-enL	Intercanthal width	Angle^c^	(A) exR-psR	Right eye angle of ex-ps
	exR-exL	Outercanthal width		(A) exL-psL	Left eye angle of ex-ps
				(A) enR-psR	Right eye angle of en-ps
				(A) enL-psL	Left eye angle of en-ps
Height^b^	psR-piR	Right palpebral fissure height		(A) enR-psR-exR	Right eye angle of en-ps-ex
	psL-piL	Left palpebral fissure height		(A) enL-psL-exL	Left eye angle of en-ps-ex
Length	enR-exR	Right palpebral fissure length	Length	psR-exR	Eye tail length
	enL-exL	Left palpebral fissure length	Ratio	psR-piR/enR-exR	Eye ratio of width to height
				(enR-exR + enL-exL)/zyR-zyL	Length ratio of eyes to face
Nose	(11 traits)				
Width^a^	sbalR-sbalL	Subnasal width	Angle^c^	(A) prn-n	Nasal bridge angle
Height^b^	(V) n -sn	Frontal nasal height		(A) prn-sn	Nasolabial angle
	n-sn	Profile nasal length		(A) n-prn-sn	Profile nasal angle
	(V) n-prn	Nasal bridge height			
	(V) prn-sn	Nasal tip height			
Depth	(H) n-prn	Nasal bridge depth			
	(H) prn-sn	Nasal tip protrusion			
Area	(AR) n-prn-sn	Profile nasal area			
Mouth	(2 traits)				
Height^b^	(V) cphR-sto	Right upper lip thickness			
	(V) cphL-sto	Left upper lip thickness			
Upper eyelid	(30 traits)				
Width^a^	(H) er1-ermax	Right eyelid peak width	Angle^c^	(A) Tan_er1~ 7	Tangent line angle of er1 ~ er7
	(H) el1-elmax	Left eyelid peak width		(A) Tan_el1~ 7	Tangent line angle of el1 ~ el7
	(H) er1-er7	Right eyelid width	Ratio	(H) er1-ermax/(H) er1-er7	Right eyelid peak position ratio
	(H) el1-el7	Left eyelid width		(H) el1-elmax/(H) el1-el7	Left eyelid peak position ratio
				(V) er1-er7/(H) er1-er7	Right eyelid slant
				(V) el1-el7/(H) el1-el7	Left eyelid slant
				(V) er1-ermax/(H) er1-ermax	Right eyelid medial slant
				(V) el1-elmax/(H) el1-elmax	Left eyelid medial slant
				(V) ermax-er7/(H) ermax-er7	Right eyelid lateral slant
				(V) elmax- el7/(H) elmax- el7	Left eyelid lateral slant
			Curvature^d^	(AC) er1-er7	Right eyelid average curvature
				(MC) er1-er7	Right eyelid maximal curvature
				(AC) el1-el7	Left eyelid average curvature
				(MC) el1-el7	Left eyelid maximal curvature

Trait *abbreviations*: *cph* crista philtri, *el* left upper eyelid, *elmax* left upper eyelid peak, *en* endocanthion, *er* right upper eyelid, *ermax* right upper eyelid peak, *ex* exocanthion, *go* gonion, *m* metopion, *mtro* metopion position on tr-o, *n* nasion, *o* ophryon, *obi* otobasion inferius, *obs* otobasion superius, *pi* palpebral inferius, *prn* pronasale, *ps* palpebral superius, *sbal* subalare, *sn* subnasale, *sto* stomion, *tr* trichion, *zy* zygion, *(A)* angle, *(AC)* average curvature, *(AR)* area, *(H)* horizontal, *(MC)* maximal curvature, *(V)* vertical

^a^Width refers to the horizontal distance between the two landmarks in a frontal (or lateral) image

^b^Height similarly refers to the vertical distance between the two landmarks in a frontal (or lateral) image

^c^Angle refers to the angle made from the line through two landmarks and a horizontal line in a frontal (or lateral) image

^d^Curvature indicates the radius of circle at a point n1 in a frontal upper eyelid

We first analyzed the correlation between body mass index (BMI) and facial traits. As shown in Additional file 1: Table S1, the correlation was the greatest for facial width traits, including facial base width, lower facial width, upper facial width, and middle facial width (*r* = 0.355–0.487). We also analyzed the correlation between sex or age and facial traits. As shown in Additional file 1: Table S1, the correlations of sex were the greatest for profile nasal area (*r* = −0.542) and upper facial area (*r* = −0.516), and the correlations of age were the greatest for eye traits such as tangent line angle of er7 or el7 (*r* = −0.306 to −0.326) and palpebral fissure heights (*r* = −0.301 to −0.312). As expected, significant correlations were observed between similar traits under the criterion of a Bonferroni-adjusted *P*-value threshold for significance of 1 × 10^− 5^ (Additional file 2: Table S2). The correlation coefficients (*r*) between facial width traits, including facial base width, lower facial width, upper facial width, and middle facial width, ranged from 0.643 to 0.928. Among the nose traits, the profile nasal area was correlated to nasal bridge depth (*r* = 0.573) and to nasal tip protrusion (*r* = 0.771).

We also estimated narrow-sense heritability for the facial traits using the GCTA program, and found moderate values for most traits (Additional file 3: Table S3). The highest heritability was detected in nasal tip protrusion (0.417), nasolabial angle (0.365), and upper lip thickness (0.299 and 0.344, right and left respectively).

### Discovery GWAS

We performed a discovery GWAS (Phase 1; *n* = 5643) and a replication analysis (Phase 2; *n* = 1926) for the 85 facial traits, as shown in Additional file 4: Figure S1. The characteristics of the participants in the two phases are described in Additional file 5: Table S4.

In total, 311,944 single nucleotide polymorphisms (SNPs) were examined in the linear regression model as independent variables of facial traits, controlled for age, sex, and BMI as covariates. The Q-Q plots of the discovery GWAS for each face trait are shown in Additional file 6: Figure S2. The GWAS results are also displayed as −log_10_(*P*) values against the chromosomal position on Manhattan plots in Additional file 7: Figure S3.

Among the 85 facial traits, seven traits, including two eye-related traits [eye tail length (psR-exR) and tangent line angle of el3 ((A) Tan_el3)] and five nose-related traits [nasal bridge depth ((H) n-prn], nasal tip protrusion [(H) prn-sn], profile nasal area [(AR) n-prn-sn], nasolabial angle [(A) prn-sn], and profile nasal angle [(A) n-prn-sn)] showed genetic associations at the genome-wide significance level (*P* < 5 × 10^− 8^) in the discovery GWAS (Additional file 8: Table S5). *SOX9*, *TBX3-MED13L*, and *VPS13B* loci met the genome-wide significance level for nose-related traits, and *WDR27* and *HOXD-MTX2* loci met the genome-wide significance level for eye shape.

### Follow-up study and meta-analysis

In the following phase, 128 SNPs (17 in frontal, 18 in forehead, 21 in eye, 50 in upper eyelid, and 40 in nose traits) were selected based on the suggestive association (*P* < 5 × 10^− 6^) in 65 facial traits, and were validated for their associations in an additional 1926 samples (Phase 2). Among the 128 variants, 117 SNPs were successfully genotyped and analyzed with related traits (Additional file 8: Table S5). Twenty-one association signals with 11 SNPs (two in face, one in forehead, one in eye, four in upper eyelid, and 13 in nose traits) were replicated with the criterion of *P* < 0.05.

**Fig. 2:**
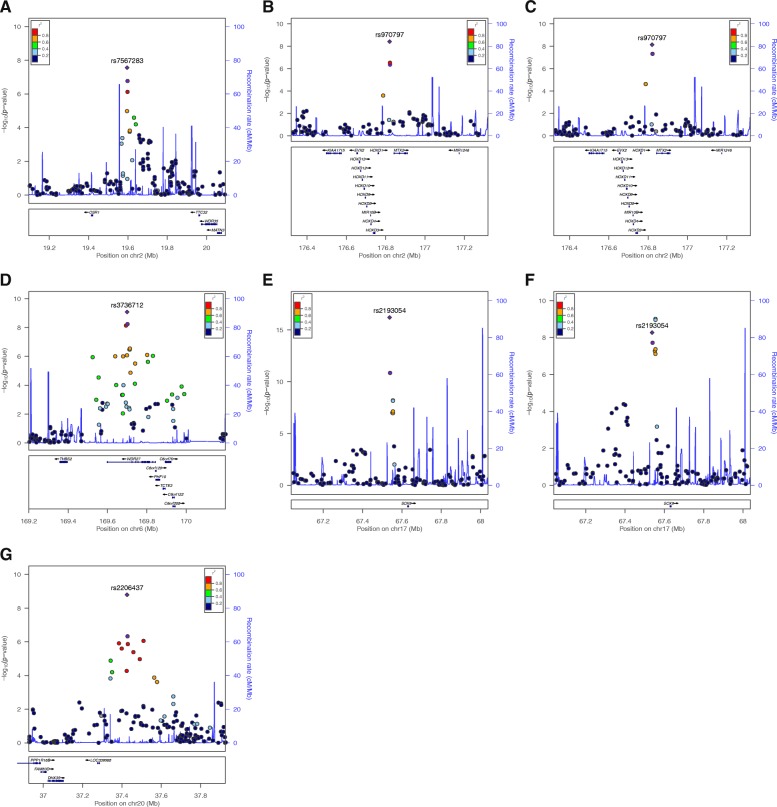
Regional association plots for five novel loci across a 1-Mb window. Association of individual SNPs in the discovery GWAS plotted as −log_10_(*P*) against the chromosomal base-pair position. The y-axis on the right shows the recombination rate, estimated from the HapMap CHB and JPT populations. *P*-values are from the discovery phase. The purple circle and diamond represent the results of discovery and meta-analysis (Phase 1 + 2), respectively. Seven signal plots for five novel SNPs are illustrated, which show the genome-wide significant *P*-values in the meta-analysis (Phase 1 + 2). **a** rs7567283 of the right facial angle of en-ex-go [(A) enR-exR-goR], **b** rs970797 of the tangent line angle of er3 [(A) Tan_er3], **c** rs970797 of the tangent line angle of el3 [(A) Tan_el3], **d** rs3736712 of eye tail length (psR-exR), **e** rs2193054 of profile nasal angle [(A) n-prn-sn], **f** rs2193054 of nasal tip protrusion [(H) prn-sn], and **g** rs2206437 of subnasal width (sbalR-sbalL)

In a meta-analysis of the results of the two cohorts (Phase 1 + 2), five loci reached genome-wide significance, including rs7567283 (the *OSR1-WDR35* locus), rs970797 (the *HOXD-MTX2* locus), rs3736712 (the *WDR27* locus), rs2193054 (the *SOX9* locus), and rs2206437 (the *DHX35* locus; Table 2). The genetic regions of these five loci and their association results are depicted as regional association plots in Fig. 2.

**Table 2:** Association results of five novel SNPs in Phase 1, Phase 2, and the Phase 1 + 2 meta-analysis

SNP	CHR:Position(bp)^a^	Gene^b^	Coded allele	Non-coded allele	Facial traits	Association results					Meta-analysis (Phase 1 + 2)			
							AF	beta ± se	*P*-value		beta ± se	*P*-value	Q	I^2^
rs7567283	2:19,595,772	*OSR1-WDR35*	G	A	Right facial angle of en-ex-go	Phase 1	0.24	−0.572 ± 0.109	1.72 × 10^−7^		− 0.536 ± 0.096	**2.75 × 10** ^**−8**^	0.48	0.00
						Phase 2	0.24	− 0.410 ± 0.205	0.046					
rs970797	2:176,820,065	*HOXD1-MTX2*	A	C	Tangent line angle of el3	Phase 1	0.33	0.017 ± 0.003	**4.90 × 10** ^**−8**^		0.015 ± 0.003	**7.40 × 10** ^**−9**^	0.29	12.52
						Phase 2	0.34	0.011 ± 0.005	0.030					
					Tangent line angle of er3	Phase 1	0.33	0.015 ± 0.003	4.51 × 10^−7^		0.015 ± 0.003	**3.97 × 10** ^**− 9**^	0.93	0.00
						Phase 2	0.34	0.014 ± 0.005	0.003					
rs3736712	6:169,699,889	*WDR27*	C	T	Eye tail length	Phase 1	0.37	0.322 ± 0.055	**5.89 × 10** ^**−9**^		0.293 ± 0.048	**8.44 × 10** ^**−10**^	0.30	6.92
						Phase 2	0.38	0.208 ± 0.095	0.029					
rs2193054	17:67,537,404	*SOX9*	C	G	Profile nasal angle	Phase 1	0.47	−0.007 ± 0.001	**1.43 × 10** ^**−11**^		−0.007 ± 0.001	**6.17 × 10** ^**− 17**^	0.22	33.67
						Phase 2	0.46	−0.009 ± 0.002	4.60 × 10^− 7^					
					Nasal tip protrusion	Phase 1	0.47	0.019 ± 0.003	**1.93 × 10** ^**−8**^		0.017 ± 0.003	**5.34 × 10** ^**−9**^	0.23	31.80
						Phase 2	0.46	0.011 ± 0.005	0.050					
rs2206437	20:37,426,155	*DHX35*	A	T	Subnasal width	Phase 1	0.26	−0.272 ± 0.054	4.75 × 10^− 7^		− 0.283 ± 0.047	**1.61 × 10** ^**−9**^	0.69	0.00
						Phase 2	0.28	− 0.316 ± 0.095	8.67 × 10^−4^					

Bold and underlined text indicates genome-wide significant *P* values (5 × 10^−8^)

*CHR* chromosome, *AF* coded allele frequency, *Q P*-value for Cochrane’s Q statistic, *I*^*2*^ heterogeneity index

^a^Positions according to NCBI Build 36

^b^Genes are defined as the gene within the SNP locates or genes closest to the SNP within a ±400-kb window when the SNP dose not locate within a gene

**Fig. 3:**
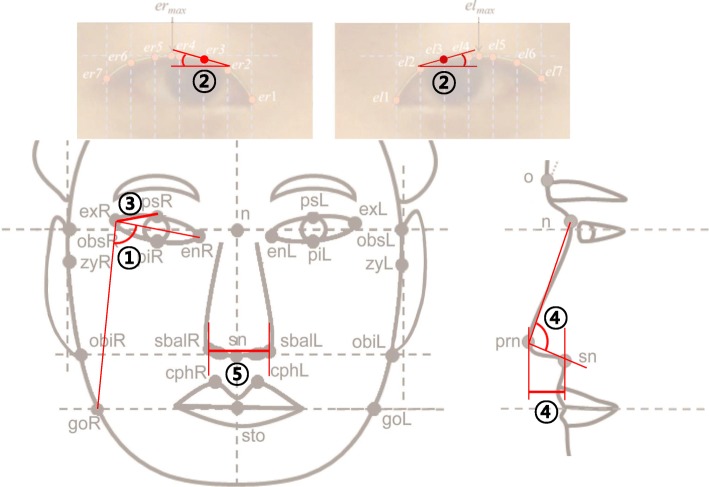
Associated facial traits for five novel SNPs. Five novel SNPs and the associated facial traits are illustrated on the frontal, lateral, and eye images. (1) rs7567283 (*OSR1-WDR35*) and right facial angle, (2) rs970797 (*HOXD1-MTX2*) and left and right curvature of the upper eyelid, (3) rs3736712 (*WDR27*) and eye tail length, (4) rs2193054 (*SOX9*) and nose shape (angle and height), and (5) rs2206437 (*DHX35*) and subnasal width

The *OSR1-WDR35* locus (rs7567283) was associated with the right facial angle of en-ex-go [(A) enR-exR-goR] (*P* = 2.75 × 10^− 8^) with regard to the face contour from a frontal view (Fig. 3). The *HOXD-MTX2* locus (rs970797) was associated with curvature of the upper eyelid, i.e., the tangent line angle of el3 [(A) Tan_el3] in the left eye (*P* = 7.40 × 10^− 9^) and tangent line angle of er3 [(A) Tan_er3] in the right eye (*P* = 3.97 × 10^− 9^), which affect eye shape. The *WDR27* locus (rs3736712) was associated with eye tail length (psR-exR) (*P* = 8.44 × 10^− 10^). The *SOX9* locus (rs2193054) exhibited the strongest signal in this study and was associated with nose shape, i.e., profile nasal angle [(A) n-prn-sn) (*P* = 6.17 × 10^− 17^) and nasal tip protrusion [(H) prn-sn] (*P* = 5.34 × 10^− 9^). The *DHX35* locus (rs2206437) was associated with the subnasal width (sbalR-sbalL) (*P* = 1.61 × 10^− 9^).

We also analyzed the phenotypic variances explained by SNP(s) (%) is obtained by R^2^ fraction of the associated SNP(s) from linear regression model (R^2^ of SNP(s) - R^2^ of covariates) (Additional file 9: Table S6). As a result, the phenotypic variances explained by the associated SNPs were lower than 1% in all SNPs.

The loci identified in this study showed multiple associations among the facial traits analyzed. Table 2 shows only the traits that met the genome-wide significance level according to the *P*-value. If the criteria were less stringently applied (i.e., *P* < 1 × 10^− 4^), the number of suggestive associated traits increased in the discovery GWAS (Additional file 10: Table S7 and Additional file 11: Figure S4). Most of the suggestive associations showed similar facial traits to those of the five novel variants identified in this study.

### Multiple signals in the *SOX9* locus

**Fig. 4:**
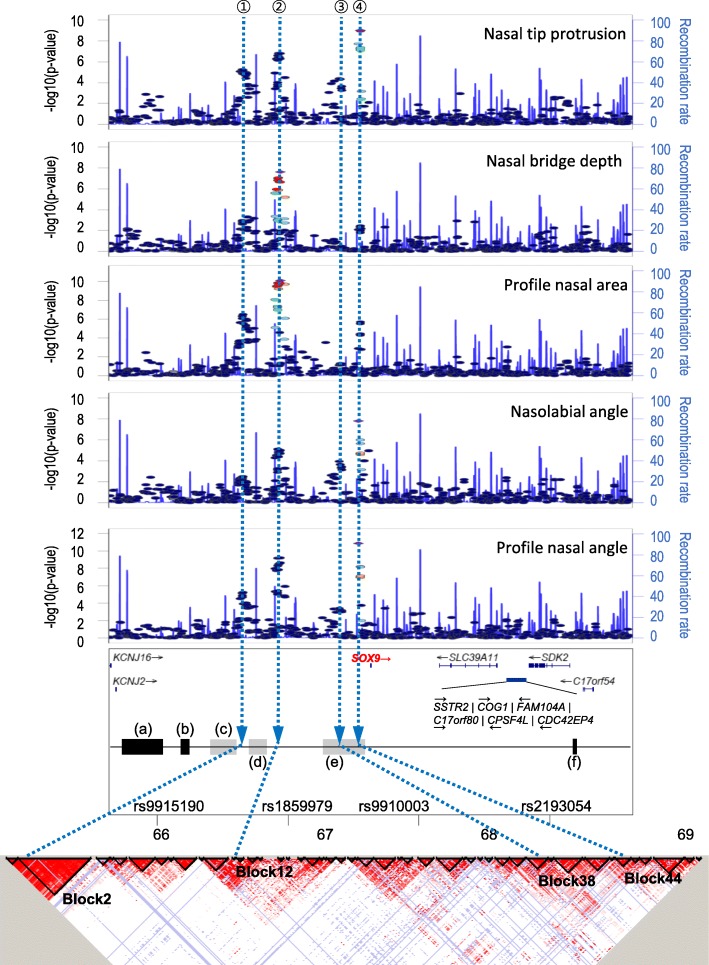
Association signals in the *SOX9* locus and genomic environment surrounding *SOX9* across a 4-Mb window. The upper five panels present multiple signals, (1) rs9915190, (2) rs1859979, (3) rs9910003, and (4) rs2193054, in the *SOX9* locus for five nose traits: nasal tip protrusion [(H) prn-sn], nasal bridge depth [(H) n-prn], profile nasal area [(AR) n-prn-sn], nasolabial angle [(A) prn-sn], and profile nasal angle [(A) n-prn-sn]. These are plotted as −log_10_(*P*) against base-pair position on chromosome 17 (Mb) and all *P*-values are from the discovery phase. The sixth panel shows genes and regulatory domains. Gray boxes represent approximate boundaries of translocation breakpoint clusters, and black boxes represent microdeletions. (a) Sp4 [31], (b) F1 [31], (c) Pierre Robin sequence (PRS) breakpoint cluster [31, 32], (d) distal breakpoint cluster [30], (e) proximal breakpoint cluster [30], and (f) Sp2 [31]. The last panel presents LD blocks based on the HapMap database (HapMap Phase II JPT + CHB, hg18)

The *SOX9* locus displayed multiple signals, as shown in regional association plots of the discovery GWAS (Fig. 4). These four signals were present in a similarly associated pattern for the five nose traits of nasal tip protrusion [(H) prn-sn], nasal bridge depth [(H) n-prn], profile nasal area [(AR) n-prn-sn], nasolabial angle [(A) prn-sn], and profile nasal angle [(A) n-prn-sn]. The SNPs representing the signals were rs2193054, rs9910003, rs1859979, and rs9915190, which were located approximately 91, 238, 688, and 974 kb upstream of the *SOX9* transcription initiation site, respectively. Both rs2193054 (91 kb) and rs1859979 (688 kb) met the genome-wide significance level in the discovery GWAS, whereas the two SNPs rs9910003 (238 kb) and rs9915190 (974 kb) showed rather weak associations.

These four variants were tested for their independence of association signals by investigating the pair-wise linkage disequilibrium (LD) and conducting conditional association analyses. The four *SOX9* SNPs showed no LD in both the Phase 1 and 1000 Genomes populations, except for weak LD detected between rs2193054 and rs9910003 (0.19 in the Phase 1 population and 0.08–0.25 in the 1000 Genomes database) (Additional file 12: Table S8). The association of rs9910003 did not persist after conditioning with rs2193054, although the associations of other variants persisted (Additional file 13: Table S9). Therefore, these SNPs (rs2193054, rs1859979, and rs9915190) were further genotyped with the 1940 Phase 2 samples for validation of their associations (Fig. 4, Table 3). These three SNPs passed the threshold of genome-wide significance after meta-analysis, and further examination of these variants with HaploReg [19] identified the promoter and enhancer histone marks, DNase hypersensitivity regions, and changed motifs in their sequences (Additional file 14: Table S10).

**Table 3:** Association results of SNPs within the *SOX9* locus (5643 discovery GWAS participants and 1940 *SOX9* replication participants)

SNP	CHR:Position (bp)^a^	Distance from 5′ of SOX9	Coded allele (AF)	Non-coded allele	Nose Traits	Association results				Meta-analysis (Phase 1 + *SOX9* replication)			
							beta ± se	*P*-value		beta ± se	*P*-value	Q	I^2^
rs9915190	7:66,654,223	974 kb	A(0.45)	C	Nasal tip protrusion	Phase 1	−0.015 ± 0.003	6.03 × 10^−6^		− 0.016 ± 0.003	**1.72 × 10** ^**− 9**^	0.72	0.00
						Phase 1 (3 SNPs conditional)	−0.016 ± 0.003	2.08 × 10^− 6^					
						*SOX9* replication set	− 0.017 ± 0.004	7.04 × 10^− 5^					
					Nasal bridge depth	Phase 1	− 0.198 ± 0.063	1.68 × 10^−3^		− 0.112 ± 0.052	3.05 × 10^− 2^	0.02	82.56
						Phase 1 (3 SNPs conditional)	−0.204 ± 0.063	1.22 × 10^− 3^					
						*SOX9* replication set	0.066 ± 0.091	4.65 × 10^−1^					
					Profile nasal area	Phase 1	− 0.017 ± 0.003	3.10 × 10^− 7^		− 0.016 ± 0.003	**2.34 × 10** ^**− 9**^	0.61	0.00
						Phase 1 (3 SNPs conditional)	− 0.017 ± 0.003	1.26 × 10^− 7^					
						*SOX9* replication set	−0.014 ± 0.005	1.91 × 10^− 3^					
					Nasolabial angle	Phase 1	0.007 ± 0.002	3.21 × 10^− 3^		0.009 ± 0.002	2.68 × 10^− 5^	0.34	0.00
						Phase 1 (3 SNPs conditional)	0.008 ± 0.002	1.69 × 10^− 3^					
						*SOX9* replication set	0.011 ± 0.004	1.73 × 10^− 3^					
					Profile nasal angle	Phase 1	0.005 ± 0.001	5.79 × 10^− 6^		0.004 ± 0.001	4.07 × 10^− 7^	0.47	0.00
						Phase 1 (3 SNPs conditional)	0.005 ± 0.001	1.02 × 10^− 6^					
						*SOX9* replication set	0.003 ± 0.001	1.83 × 10^− 2^					
rs1859979	7:66,940,738	688 kb	C(0.46)	T	Nasal tip protrusion	Phase 1	0.018 ± 0.003	1.56 × 10^− 7^		0.017 ± 0.003	**1.91 × 10** ^**− 10**^	0.68	0.00
						Phase 1 (3 SNPs conditional)	0.017 ± 0.003	7.50 × 10^− 7^					
						*SOX9* replication set	0.016 ± 0.004	2.98 × 10^− 4^					
					Nasal bridge depth	Phase 1	0.331 ± 0.064	2.21 × 10^− 7^		0.272 ± 0.052	1.92 × 10^− 7^	0.11	61.76
						Phase 1 (3 SNPs conditional)	0.323 ± 0.064	4.76 × 10^− 7^					
						*SOX9* replication set	0.152 ± 0.091	9.33 × 10^− 2^					
					Profile nasal area	Phase 1	0.022 ± 0.003	**8.07 × 10** ^**− 11**^		0.018 ± 0.003	**7.22 × 10** ^**− 12**^	0.09	64.77
						Phase 1 (3 SNPs conditional)	0.020 ± 0.003	**1.39 × 10** ^**− 9**^					
						*SOX9* replication set	0.012 ± 0.005	6.59 × 10^− 3^					
					Nasolabial angle	Phase 1	−0.011 ± 0.002	9.86 × 10^− 6^		− 0.013 ± 0.002	**1.13 × 10** ^**− 10**^	0.11	60.21
						Phase 1 (3 SNPs conditional)	− 0.011 ± 0.002	2.10 × 10^− 5^					
						*SOX9* replication set	−0.018 ± 0.004	8.09 × 10^− 7^					
					Profile nasal angle	Phase 1	− 0.006 ± 0.001	**2.37 × 10** ^**− 9**^		− 0.007 ± 0.001	**9.58 × 10** ^**− 15**^	0.63	0.00
						Phase 1 (3 SNPs conditional)	− 0.006 ± 0.001	**2.53 × 10** ^**− 9**^					
						*SOX9* replication set	− 0.007 ± 0.001	8.17 × 10^− 7^					
rs9910003	7:67,390,279	238 kb	A(0.28)	G	Nasal tip protrusion	Phase 1	0.015 ± 0.004	4.05 × 10^− 5^					
						Phase 1 (3 SNPs conditional)	0.007 ± 0.004	9.07 × 10^− 2^					
						*SOX9* replication set							
					Nasal bridge depth	Phase 1	0.038 ± 0.071	5.90 × 10^− 1^					
						Phase 1 (3 SNPs conditional)	− 0.077 ± 0.079	3.33 × 10^− 1^					
						*SOX9* replication set							
					Profile nasal area	Phase 1	0.007 ± 0.004	7.71 × 10^− 2^					
						Phase 1 (3 SNPs conditional)	0.001 ± 0.004	8.50 × 10^− 1^					
						*SOX9* replication set							
					Nasolabial angle	Phase 1	−0.011 ± 0.003	1.12 × 10^− 4^					
						Phase 1 (3 SNPs conditional)	−0.004 ± 0.003	1.99 × 10^− 1^					
						*SOX9* replication set							
					Profile nasal angle	Phase 1	−0.004 ± 0.001	3.87 × 10^− 4^					
						Phase 1 (3 SNPs conditional)	0.000 ± 0.001	8.06 × 10^− 1^					
						*SOX9* replication set							
rs2193054	17:67,537,404	91 kb	C(0.47)	G	Nasal tip protrusion	Phase 1	0.019 ± 0.003	**1.93 × 10** ^**− 8**^		0.017 ± 0.003	**1.85 × 10** ^**− 10**^	0.35	0.00
						Phase 1 (3 SNPs conditional)	0.015 ± 0.004	3.26 × 10^− 5^					
						*SOX9* replication set	0.013 ± 0.004	1.74 × 10^− 3^					
					Nasal bridge depth	Phase 1	0.165 ± 0.062	8.02 × 10^−3^		0.130 ± 0.051	1.10 × 10^− 2^	0.33	0.00
						Phase 1 (3 SNPs conditional)	0.211 ± 0.070	2.50 × 10^− 3^					
						*SOX9* replication set	0.057 ± 0.091	5.30 × 10^− 1^					
					Profile nasal area	Phase 1	0.010 ± 0.003	1.33 × 10^− 3^		0.010 ± 0.003	8.14 × 10^− 5^	0.98	0.00
						Phase 1 (3 SNPs conditional)	0.011 ± 0.004	3.62 × 10^− 3^					
						*SOX9* replication set	0.010 ± 0.005	2.25 × 10^−2^					
					Nasolabial angle	Phase 1	−0.014 ± 0.002	**1.56 × 10** ^**− 8**^		− 0.013 ± 0.002	**2.25 × 10** ^**− 10**^	0.49	0.00
						Phase 1 (3 SNPs conditional)	−0.012 ± 0.003	7.34 × 10^− 6^					
						*SOX9* replication set	− 0.011 ± 0.004	3.35 × 10^− 3^					
					Profile nasal angle	Phase 1	− 0.007 ± 0.001	**1.43 × 10** ^**− 11**^		− 0.006 ± 0.001	**9.01 × 10** ^**− 14**^	0.24	27.90
						Phase 1 (3 SNPs conditional)	−0.007 ± 0.001	**1.50 × 10** ^**− 9**^					
						*SOX9* replication set	− 0.005 ± 0.001	8.53 × 10^− 4^					

Bold and underlined text indicates genome-wide significant *P* values (5 × 10^− 8^)

*CHR* chromosome, *AF* coded allele frequency, *Q P*-value for Cochrane’s Q statistic, *I*^*2*^ heterogeneity index

^a^Positions according to NCBI Build 36

### Replication analysis of previously reported facial trait GWAS loci

We performed a replication analysis for previously reported face morphology GWAS loci [12–17] (Table 4). A total of 41 lead SNPs were selected for this analysis. Since the Affymetrix Genome-Wide Human SNP array 5.0 genotype platform did not include these SNPs, except for rs6555969 (*C5orf50*), we extracted proxy SNPs of these 41 lead SNPs using LDlink (https://analysistools.nci.nih.gov/LDlink) [20] under the criterion of r^2^ > 0.6. After filtering low-frequency SNPs [minor allele frequency (MAF) < 0.05], X chromosome SNPs, and GWAS QC-failed SNPs, 63 proxy SNPs from 13 lead SNPs were selected and examined for their association with relevant facial traits examined in this study. As shown in Table 4, one proxy SNP per lead SNP that showed the highest LD (r^2^) value among the proxy SNPs was identified.

**Table 4:** Replication analysis of previous facial morphology GWASs

Previously reported index SNP	Gene^a^	Previously reported association phenotype	Reference	Tested SNP	LD (r^2^)^b^	Coded allele	Non-coded allele	Associated facial traits in Phase 1	Association results	
									beta ± se	*P*-value
rs7559271	*PAX3*	Nasion position,n-men	Adhikari, et al. [14] Paternoster et al. [12]	rs11683100	0.70	G	T	Profile nasal area	0.007 ± 0.004	0.043
rs4648379	*PRDM16*	AlrR-Prn, AlrL-Prn	Liu et al. [13]	rs4648478	0.65	G	A	Profile nasal angle	−0.005 ± 0.001	5.70 × 10^− 6^
rs6555969	*C5orf50*	ZygR-Nsn,ZygL-Nsn,EyeR-Nsn,EyeL-Nsn	Liu et al. [13]	rs6555969	1.00	T	C	Right palpebrale fissure length	0.086 ± 0.069	0.210
rs805722	*COL17A1*	EyeR-Nsn,EyeL-Nsn	Liu et al. [13]	rs805693	0.97	T	C	Left palpebral fissure height	−0.039 ± 0.036	0.288
rs2045323	*DCHS2*	Columella inclination,Nose protrusion,Nose tip angle	Adhikari et al. [14]	rs4315762	0.68	G	C	Subnasal width	−0.247 ± 0.054	4.84 × 10^−6^
rs1852985	*SUPT3H/RUNX2*	Nose bridge breadth	Adhikari et al. [14]	rs1284964	1.00	G	A	Nasal bridge angle	0.429 ± 0.101	2.28 × 10^−5^
rs2424399	*PAX1*	Nasal width	Shaffer et al. [15]	rs6082475	1.00	G	A	Subnasal width	0.164 ± 0.049	7.28 × 10^−4^
rs9868698	*SCHIP1*	PC4(facial height, nasal width)	Cole et al. [16]	rs627865	0.98	G	C	Subnasal width	0.036 ± 0.051	0.485
rs2817419	*TFAP2B*	EX_R_EX_L(outer canthal width)	Cole et al. [16]	rs3857597	0.77	T	C	Outercanthal width	0.228 ± 0.138	0.098
rs35965172	*TFAP2B*	EN_EX(Palpebral fissure length)	Cole et al. [16]	rs3857597	0.78	T	C	Right eyelid width	0.011 ± 0.008	0.196
rs7836044	*intergenic*	STO_SL(lower lip height)	Cole et al. [16]	rs3850505	0.99	C	T	Left upper lip thickness	−0.006 ± 0.004	0.163
rs9456748	*PARK2*	factor 9(Facial height related to the vertical position of nasion; Nasion)	Lee et al. [17]	rs6904579	0.86	G	A	Facial height	−0.212 ± 0.099	0.033
rs5781117	*LYPLAL1*	nose size	Pickrell et al. [21]	rs2605100	0.78	T	C	Profile nasal angle	0.003 ± 0.001	0.030
rs424737	*ROBO1*	nose size	Pickrell et al. [21]	rs333472	1.00	C	T	Nasal tip height	0.007 ± 0.003	6.58 × 10^−3^
rs2929451	*PPP1R3B*	nose size	Pickrell et al. [21]	rs2929453	0.94	G	A	Nasal tip protrusion	−0.009 ± 0.007	0.212
rs10779169	*RASSF9*	nose size	Pickrell et al. [21]	rs2405254	0.99	T	A	Profile nasal angle	0.003 ± 0.001	8.30 × 10^−3^
rs11782517	*MSRA*	nose size	Pickrell et al. [21]	rs12544801	0.90	G	C	Nasolabial angle	−0.005 ± 0.003	0.081
rs767764	*DLC1*	nose size	Pickrell et al. [21]	rs2278945	0.71	G	A	Nasal bridge angle	−0.307 ± 0.092	8.94 × 10^−4^
rs34702092	*RAD51B*	nose size	Pickrell et al. [21]	rs11624333	1.00	G	A	Nasal bridge angle	−0.438 ± 0.156	5.02 × 10^−3^
rs6101567	*DHX35*	nose size	Pickrell et al. [21]	rs6129346	0.99	C	T	Frontal nasal height	−0.299 ± 0.076	7.53 × 10^−5^

*AlrL* alare left, *AlrR* alare right, *Prn* pronasale, *ZygL* zygion left, *ZygR* zygion right, *Nsn* nasion, *EyeL* eyeball left, *EyeR* eyeball right

^a^Genes are defined as previously reported

^b^r^2^ is calculated based on data from phase 3 of the 1000 Genomes Project in the Asian panel (Chinese and Japanese)

The rs4648379 locus was previously associated with nose width and nose height [13], and its proxy SNP rs4648478 (r^2^ = 0.61) showed an association of *P* = 5.70 × 10^− 6^ with profile nasal angle [(A) n-prn-sn] in our analysis. Because there were 11 nose-related traits in this study, the criterion of replication significance was set to *P* = 4.5 × 10^− 3^ considering the Bonferroni multiple correction. Therefore, the SNP rs4648379 appeared to be replicated in this study. Similarly, the locus rs2045323 was reported to be associated with three nose phenotypes (columella inclination, nose protrusion, and nose tip angle) [14], and this locus was replicated in this study, demonstrating an association with subnasal width (sbalR-sbalL) (proxy SNP rs4315762, r^2^ = 0.68, *P* = 4.84 × 10^− 6^). Another SNP, rs1852985, associated with nose bridge breadth [14], was also replicated with nasal bridge angle [(A) prn-n] (rs1284964, r^2^ = 0.85, *P* = 2.28 × 10^− 5^), and rs2424399, previously associated with nasal width [15], was replicated with subnasal width (sbalR-sbalL) (rs6082475, r^2^ = 1.0, *P* = 7.28 × 10^− 4^).

In addition, we performed another replication analysis of the SNPs identified by Pickrell et al. [21], who used self-reported information in a very large cohort (*n* > 70,000). They identified 23 lead SNPs associated with nose size. After applying the same filtering process described above, eight lead SNPs were further selected for analysis in our sample, and their 40 proxy SNPs were examined for association with nose traits. As a result, two SNPs, rs767764 and rs6101567, were replicated in the present study (Table 4).

## Discussion

Through the first two-stage GWAS on facial morphology in the Korean population, we identified five genetic loci that were significantly associated with facial traits, including facial frontal contour, eye shape, and nose shape. Only one SNP, rs97097, in the *HOXD-MTX2* locus was found in the GWAS catalogue and the UK Biobank, which has been associated with monobrow and earlobe attachment [15, 22]. In addition, during the review process of this paper, Claes et al. reported the facial trait association of two SNPs in *HOXD1-MTX2* and *SOX9* (rs970797 and rs5821892, respectively) [23].

### Face morphology GWAS identified genes for craniofacial malformations

Among the associations, two loci seemed to be related to known craniofacial malformations. The SNP rs2193054 in the *SOX9* locus is located at the proximal break point cluster region of campomelic dysplasia (CMPD [MIM 114290]). CMPD is caused by mutations in the *SOX9* gene at 17q24.3 and mainly manifests as skeletal defects such as tubular bone bowing by autosomal dominant inheritance (Additional file 15: Table S11) [24, 25]. CMPD frequently presents as abnormal facial features, including a flat face and depressed nasal bridge, suggesting an important role for *SOX9* in craniofacial chondrocyte differentiation.

Another variant, rs7567283 in 2p24.1, was located 378 kb downstream of the *WDR35* gene, which belongs to the WD repeat family, providing a beta propeller scaffold for the assembly of multiple protein complexes [26]. The *WDR35* gene is related to cranioectodermal dysplasia 2 (CED2 [MIM 613610]), which manifests as forehead bossing, dolichocephaly, and metaphyseal dysplasis [27]. Dolichocephaly is a condition in which the head is longer than expected relative to its width and is correlated with frontal face width traits associated with the variant rs7567283 in the *WDR35* locus (Additional file 15: Table S11).

### Allele frequencies of associated variants are different among ethnic groups

Interestingly, four out of five associated variants showed differences in allele frequencies among ethnic groups, suggesting that these variants may contribute to facial differences among ethnic groups (Additional file 16: Table S12). The reference allele G in rs7567283 (*OSR1-WDR35*) resulted in a narrower frontal face than that of the alternate allele (A). Based on the 1000 Genome Project data, the G allele is less frequent in East Asians (24%) than in both Europeans (82%) and Africans (67%). Similarly, the reference allele C in rs3736712 (*WDR27*) results in longer eye tail lengths than alternate alleles and is less frequent in East Asians (39%) than in both Europeans (93%) and Africans (93%).

The reference allele C of rs2193054 (*SOX9*) results in higher nasal protrusion than the alternate allele and is less frequent in Africans (24%) than in both East Asians (45%) and Europeans (50%). The profile nose size-increasing alleles of the three other conditionally independent *SOX9* variants (rs9915190, rs1859979, and rs9910003) are less frequent in East Asians that in both Europeans and Africans (54, 44, and 30% in East Asians versus 64, 95, and 46% in Europeans and 83, 90, and 57% in Africans, respectively). The T allele of rs2206437 (*DHX35*) results in a wider and lower nose than the alternate allele A from the front view and is more frequent in both East Asians (77%) and Africans (75%) than in Europeans (52%). Thus, we found several significant variants for nose shape manifesting differential allele frequencies among different ethnic populations. Indeed, several previous GWASs have identified variants associated with nose traits (Table 4), suggesting that nasal traits are highly heritable in humans [28].

### Multiple association signals in the *SOX9* locus confirmed the long-range regulation of the *SOX9* gene

Three association signals of rs2193054 (91 kb), rs1859979 (688 kb), and rs9915190 (974 kb) upstream of the *SOX9* gene were identified as independently associated based on both the LD block and conditional analyses. Consistent with these findings, genetic analyses of patients with CMPD and Pierre Robin sequence (PRS, [MIM 261800]) revealed multiple translocation breakpoints and deletions in the ~ 3-Mb region spanning the *SOX9* coding region, indicating that *SOX9* may be modulated by multiple regulatory sequences within the long-distance range [29]. The proximal breakpoint cluster [30] [50–375 kb, depicted as (e) in the lower gene map panel of Fig. 4], distal breakpoint cluster [30] [789–932 kb, Fig.4 (d)], and PRS breakpoint cluster [31, 32] [~ 1.13 Mb, Fig. 4 (c)] are translocation breakpoints upstream of the *SOX9* gene, and deletions were identified throughout the region over 1 Mb upstream of *SOX9* [Fig. 4 (a) and (b)] and in the downstream region [Fig. 4 (f)] [31, 33].

To gain more insights into the regulatory functions of the associated variants, we aligned the SNPs with mutation sites found in patients with CMPD and PRS. The location of rs2193054 closest to the *SOX9* gene corresponding to the proximal breakpoint cluster region rs1859979 was just downstream of the distal breakpoint cluster, and rs9915190 was located between the PRS and the distal breakpoint cluster. Therefore, patients with CMPD harboring a chromosomal break in the proximal breakpoint cluster may lose both regulatory elements at 688 kb (rs1859979, (2) in the lower gene map panel of Fig. 4) and 974 kb (rs9915190, (1) in Fig. 4), whereas patients with CMPD harboring a break in the distal breakpoint cluster may lose only the element at 974 kb upstream of the *SOX9* gene.

### Study limitations

The main limitation of the study was that the facial measurements were derived from two-dimensional images rather than three-dimensional images, which could be the gold standard in the GWAS of facial traits. Given this limitation, we tried to measure more facial traits than evaluated in previous studies, such as the angles, ratios, and curvatures, as well as conventional distances including widths and heights.

## Conclusion

We identified five novel face morphology loci that were associated with facial frontal contour, nose shape, and eye shape. Our findings were further emphasized by three observations: 1) two of the loci have been implicated in craniofacial malformations; 2) the allele frequencies of four variants differed among ethnic groups; and 3) the *SOX9* locus contained three independent association signals. Thus, face morphology GWASs may expand our understanding of craniofacial malformations and provide useful genetic information for externally visible characteristics in forensic science.

## Methods

### Study participants

The participants for the discovery GWAS (Phase 1) were recruited from two regions in South Korea (Ansan and Ansung) from 2009 to 2012 for the Korean Genome and Epidemiology Study (KoGES) [34]. The criterion for inclusion of participants in the study was the availability of facial images and genome-wide genotype data. The criteria for exclusion were as follows: a history of cancer, gender inconsistencies, cryptic relatedness, low genotype call rate (< 95%), and sample contamination, as previously described [34]. A total of 5643 individuals (2648 men and 2995 women) met the requirements and were selected for the discovery GWAS (Phase 1).

The follow-up analysis (Phase 2) was performed using 2009 individuals from 19 Oriental medical clinics recruited from 2007 to 2012 for the Korea Constitution Multicenter Study (KCMS). Among the 2009 study participants, we extracted 1926 samples (687 men and 1239 women) who were over the age of 20 and showed high genotype call rates (≥95%).

To validate the multiple association signals in the *SOX9* locus, we used another independent replication set of participants recruited from 2011 to 2012 for the Korea Constitution Multicenter Study (KCMS). After applying the above-mentioned inclusion and exclusion criteria, 1940 individuals (587 men and 1353 women) were used to confirm the association between nose traits and multiple variants within the *SOX9* locus.

### Facial traits

The overall procedure for feature extraction is shown in Additional file 17: Figure S5. The participants were confirmed to not be wearing any cosmetic make-up and were photographed from both frontal and lateral views using a digital camera (DSLR Nikon D90 with a Nikon AF 50-mm F1.8D lens, 3216 × 2136 pixels) under the following standard conditions: the hair was pulled back with a hair band; the center points of the two pupils and the two points connecting the facial contour and upper auricular perimeters (e.g., points obsR and obsL from the frontal image shown in Fig. 1) were on the same horizontal line; and a ruler was placed approximately 10 mm below the chin to convert pixels into millimeters [35].

Facial feature points in frontal and lateral images were automatically extracted by detecting and analyzing the face, eyes, nose, mouth, and contours via our own developed program in Visual Studio C++ with the use of OpenCV (Open Source Computer Vision Library). Given an input image, the region of interest (ROI) was reduced in stages by detecting the face, eyes, nose, and mouth sequentially with Adaboost-based detectors [36, 37]. In each ROI, the facial feature points were found from facial contours obtained through histogram-based image segmentation. The positions of the extracted points were confirmed by a well-trained operator, and the same extraction procedure for facial feature points was applied to both the discovery and replication sets. The accuracy of the automatic landmarking was 98.8% on average, and we excluded the data for cases with inaccurate landmarking.

The facial traits analyzed in the study were delineated by distance, distance ratio, angle, area, and eye curvature from 23 frontal and seven lateral facial points (Fig. 1 and Table 1). The detailed descriptions of these traits were presented in previous studies [38, 39]. For linear regression analysis, 15 severely skewed facial traits, including upper forehead slant depth [(H) tr-m], brow ridge protrusion [(H) o-n], metopion eminence depth (m-mtro), left eyelid average curvature [(AC) el1-el7], left eyelid maximal curvature [(MC) el1-el7], right eyelid average curvature [(AC) el1-el7], right eyelid maximal curvature [(MC) er1-er7], profile nasal length (n-sn), nasal tip height [(V) prn-sn], nasal tip protrusion [(H) prn-sn], profile nasal area [(AR) n-prn-sn], nasolabial angle [(A) prn-sn], profile nasal angle [(A) n-prn-sn], right upper lip thickness [(V) cphR-sto], and left upper lip thickness [(V) cphL-sto], were ln-transformed. We removed outliers in each facial trait, which were defined using the first and third quartiles and interquartile range of each facial trait. In each facial trait, outliers under the first quartile – 2.0 × interquartile range or over the third quartile + 2.0 × interquartile range were excluded.

### Genotyping

Genotyping of DNA from the discovery GWAS (Phase 1) population was performed using an Affymetrix Genome-Wide Human SNP array 5.0 (Affymetrix, Santa Clara, CA, USA), as described in a previous report [34]. Among 500,568 SNPs in the Affymetrix SNP array, 311,944 autosomal SNPs were examined in the GWAS, after excluding SNPs with a high missing call rate (> 5%), low MAF (< 0.05), and deviation from Hardy-Weinberg equilibrium (*P* < 0.0001).

For the follow-up of SNPs that met the suggestive threshold of association with facial traits (*P* < 5 × 10^− 6^), we conducted LD pruning and selected the 128 sentinel SNPs that tagged each locus. Replication genotyping of the 1926 samples (Phase 2) was performed using an Applied Biosystems QuantStudio 12 K Flex Real-Time PCR System. Genotyping quality was controlled by excluding SNPs with low call rates (< 95%), low MAF (< 0.01), and bad genotype clustering. Individuals with a high missing genotype call rate (> 5%) were excluded from analysis.

The genotypes of three variants (rs9915190, rs1859979, and rs2193054) within the *SOX9* locus were determined using an unlabeled oligonucleotide probe (UOP) on a polymorphic nucleotide for 1940 participants (*SOX9* replication set). The detailed process of genotyping using a UOP for the variant was described in a previous report [40, 41]. An aliquot of the polymerase chain reaction amplicon including the SNP site was diluted in a solution containing 1 mM UOP, 5 mM SYTO-9 (Invitrogen, Carlsbad, CA, USA), 12.5 mM EDTA, and 10 mM Tris (pH 8.0). The DNA in the UOP sample sequentially underwent denaturation (95 °C for 5 s), annealing (60 °C for 1 min), and melting with a gradual increase to 74 °C at a rate of 1 °C/s; the fluorescence emission was read using a Light Cycler 480 instrument (Roche, Indianapolis, IN, USA). The genotype was determined from three melting patterns of the UOP (major homozygote, heterozygote, and minor homozygote).

### Statistical analysis

GWASs were performed for discovering variants associated with facial traits such as frontal and lateral images of the eye, nose, contour, and other features, using PLINK version 1.09 [42] by linear regression analysis in an additive model, with adjustment for age, sex, and BMI. The cut-off *P*-value for discovery GWAS (Phase 1) was 5.0 × 10^− 6^. Quantile-quantile plots for facial traits were constructed with the distribution of observed *P-*values against the theoretical distribution of expected *P*-values. A regional association plot for a genomic region of 1 Mb centered on the peak SNP was constructed using LocusZoom [43].

In the follow-up analysis (Phase 2), multiple linear regression analysis was performed to determine the association of 117 SNPs in 1926 participants with the corresponding facial traits, with adjustment for age, sex, and BMI, using PLINK v1.09.

Conditional analyses were performed to identify the SNP(s) in the *SOX9* locus that were independently associated with nose traits in Phase 1 subjects. Multiple linear regression analysis of each *SOX9* SNP was carried out for each nose trait, with adjustment for age, sex, BMI, and another *SOX9* SNP (or the three other *SOX9* SNPs together). In addition, we carried out multiple linear regression analysis using R version 3.0.2 to validate multiple association signals for nose traits in another independent replication set including 1940 participants (*SOX9* replication set).

All meta-analysis calculations were implemented in PLINK and METAL [44] under the assumption of fixed effects using Cochran’s Q test to determine between-study heterogeneity. The SNPs in the combined analysis were considered significant when *P*-values were below 5.0 × 10^− 8^ (as the traditional genome-wide significance level).

For eQTL analysis, databases from GTExPortal [45] and BRAINEAC [46] were used, along with additional data from Schadt et al. [47], Westra et al. [48], and Fairfax et al. [49]. Functional annotations such as chromatin structure, methylation, protein motifs, and transcription factor binding were summarized using HaploReg [19], and functional variant scores were calculated using RegulomeDB [50].

## Additional files

Additional file 1: **Table S1.** Correlation between face traits and covariates. (XLSX 18 kb)

Additional file 2: **Table S2.** Correlations of face traits. (XLSX 88 kb)

Additional file 3: **Table S3.** Heritability of 85 facial traits from the Phase 1 population. (DOCX 19 kb)

Additional file 4: **Figure S1.** Study design for facial morphology GWASs. (PDF 116 kb)

Additional file 5: **Table S4.** Characteristics of the study participants. (DOCX 32 kb)

Additional file 6: **Figure S2.** Q-Q plots for the discovery GWAS (85 facial traits). (PDF 1945 kb)

Additional file 7: **Figure S3.** Manhattan plots for the discovery GWAS (85 facial traits). (PDF 2645 kb)

Additional file 8: **Table S5.** Association results in Phase 1, Phase 2, and the Phase 1 + 2 meta-analysis (63 phenotypes, 117 SNPs). (DOCX 78 kb)

Additional file 9: **Table S6.** Phenotypic variance explained by five face-associated SNPs shown in Table 2 in the Phase 1 population (DOCX 15 kb)

Additional file 10: **Table S7.** Additional associated facial traits of five novel SNPs in the discovery GWAS (*P* < 0.0001). (DOCX 18 kb)

Additional file 11: **Figure S4.** Multiple associations of five loci among the 85 facial traits in the discovery GWAS. (PDF 266 kb)

Additional file 12: **Table S8.** Pairwise linkage disequilibrium analyses with four nose-associated SNPs in the *SOX9* locus. (DOCX 16 kb)

Additional file 13: **Table S9.** Results of conditional analysis for four variants in the upstream region of *SOX9* (Phase 1). (DOCX 34 kb)

Additional file 14: **Table S10.** Functional analysis of three variants in the upstream region of *SOX9*. (DOCX 15 kb)

Additional file 15: **Table S11.** Comparison of clinical phenotypes and facial traits. (DOCX 14 kb)

Additional file 16: **Table S12.** Comparisons of allele frequencies between Koreans and other populations from the 1000 Genomes Project Phase 3. (DOCX 16 kb)

Additional file 17: **Figure S5.** The procedure for facial feature point extraction. (PDF 362 kb)

## Abbreviations

BMI: Body mass index
CED2: Cranioectodermal dysplasia 2
CMPD: Campomelic dysplasia
GWAS: Genome-wide association study
KCMS: Korea Constitution Multicenter Study
KoGES: Korean Genome and Epidemiology Study
LD: Linkage disequilibrium
MAF: Minor allele frequency
OpenCV: Open Source Computer Vision Library
PRS: Pierre Robin sequence
ROI: Region of interest
SNP: Single nucleotide polymorphism
UOP: Unlabeled oligonucleotide probe
