# Supplementary material for: Identification of five novel genetic loci related to facial morphology by genome-wide association studies
Source: BMC Genomics. 2018 Jun 19;19:481. doi: 10.1186/s12864-018-4865-9 (PMC6008943; doi:10.1186/s12864-018-4865-9)
Supplement: Supplementary file 3 — Table S3. Heritability of 85 facial traits from the Phase 1 population. (DOCX 19 kb) [file 12864_2018_4865_MOESM3_ESM.docx]

**Table S3.** Heritability of 85 facial traits from the Phase 1 population

| **Facial traits** | | **Heritability** | **SE** | **P-value** |
| --- | --- | --- | --- | --- |
| Face shape | Facial base width | 0.230 | 0.052 | 9.87E-07 |
|  | Lower facial width | 0.146 | 0.050 | 5.45E-04 |
|  | Upper facial width | 0.260 | 0.052 | 2.54E-08 |
|  | Middle facial width | 0.192 | 0.051 | 9.35E-06 |
|  | Upper lip height | 0.208 | 0.052 | 2.42E-05 |
|  | Facial height | 0.212 | 0.052 | 5.90E-06 |
|  | Upper facial area | 0.231 | 0.052 | 5.78E-07 |
|  | Lower facial area | 0.203 | 0.052 | 2.08E-05 |
|  | Facial width ratio of base to chin | 0.148 | 0.051 | 1.34E-03 |
|  | Facial ratio of base width to height | 0.183 | 0.052 | 2.18E-04 |
|  | Facial ratio of chin width to height | 0.166 | 0.052 | 5.71E-04 |
|  | Right facial angle of en-ex-go | 0.253 | 0.052 | 3.28E-07 |
|  | Left facial angle of en-ex-go | 0.096 | 0.050 | 1.89E-02 |
|  | Right facial angle of ps-ex-go | 0.214 | 0.052 | 1.05E-05 |
|  | Left facial angle of ps-ex-go | 0.132 | 0.051 | 4.52E-03 |
|  | Right facial angle of en-ps-go | 0.132 | 0.052 | 4.77E-03 |
|  | Left facial angle of en-ps-go | 0.118 | 0.050 | 5.90E-03 |
| Forehead | Forehead height | 0.059 | 0.050 | 1.05E-01 |
|  | Lower forehead height | 0.018 | 0.053 | 3.74E-01 |
|  | Upper forehead height | 0.016 | 0.043 | 3.34E-01 |
|  | Brow ridge height | 0.165 | 0.054 | 4.83E-04 |
|  | Upper forehead slant angle | 0.178 | 0.053 | 3.41E-04 |
|  | Brow ridge protrusion angle | 0.220 | 0.054 | 1.49E-05 |
|  | Upper forehead slant depth | 0.142 | 0.052 | 1.24E-03 |
|  | Brow ridge protrusion | 0.023 | 0.050 | 3.29E-01 |
|  | Metopion position ratio | 0.000001 | 0.052 | 5.00E-01 |
|  | Metopion eminence depth | 0.156 | 0.054 | 1.47E-03 |
| Eye | Intercanthal width | 0.256 | 0.053 | 4.32E-07 |
|  | Outercanthal width | 0.241 | 0.051 | 4.57E-08 |
|  | Right palpebral fissure height | 0.090 | 0.048 | 1.56E-02 |
|  | Left palpebral fissure height | 0.075 | 0.047 | 3.31E-02 |
|  | Right palpebrale fissure length | 0.134 | 0.050 | 1.42E-03 |
|  | Left palpebrale fissure length | 0.056 | 0.045 | 7.11E-02 |
|  | Right eye angle of ex-ps | 0.202 | 0.051 | 3.02E-05 |
|  | Left eye angle of ex-ps | 0.183 | 0.051 | 1.15E-04 |
|  | Right eye angle of en-ps | 0.103 | 0.050 | 1.06E-02 |
|  | Left eye angle of en-ps | 0.144 | 0.051 | 1.49E-03 |
|  | Right eye angle of en-ps-ex | 0.109 | 0.049 | 8.77E-03 |
|  | Left eye angle of en-ps-ex | 0.137 | 0.050 | 2.67E-03 |
|  | Eye tail length | 0.072 | 0.050 | 7.38E-02 |
|  | Eye ratio of width to height | 0.073 | 0.049 | 5.93E-02 |
|  | Ratio of eye width to base width | 0.111 | 0.050 | 8.47E-03 |
| nose | Subnasal width | 0.285 | 0.053 | 8.27E-09 |
|  | Frontal nasal height | 0.216 | 0.052 | 4.58E-06 |
|  | Profile nasal length | 0.157 | 0.053 | 7.53E-04 |
|  | Nasal bridge height | 0.283 | 0.055 | 7.86E-08 |
|  | Nasal tip height | 0.117 | 0.053 | 1.36E-02 |
|  | Nasal bridge depth | 0.195 | 0.053 | 7.24E-05 |
|  | Nasal tip protrusion | 0.417 | 0.055 | 4.55E-15 |
|  | Profile nasal area | 0.280 | 0.055 | 6.80E-08 |
|  | Nasal bridge angle | 0.217 | 0.053 | 1.60E-05 |
|  | Nasolabial angle | 0.365 | 0.055 | 8.32E-12 |
|  | Profile nasal angle | 0.279 | 0.055 | 1.42E-07 |
| mouth | Right upper lip thickness | 0.299 | 0.054 | 5.57E-09 |
|  | Left upper lip thickness | 0.344 | 0.053 | 6.41E-12 |
| Upper eyelid | Right eyelid peak width | 0.145 | 0.051 | 1.09E-03 |
|  | Left eyelid peak width | 0.023 | 0.045 | 2.88E-01 |
|  | Right eyelid width | 0.117 | 0.050 | 6.75E-03 |
|  | Left eyelid width | 0.001 | 0.050 | 4.95E-01 |
|  | Tangent line angle of er1 | 0.058 | 0.051 | 1.34E-01 |
|  | Tangent line angle of er2 | 0.092 | 0.051 | 4.01E-02 |
|  | Tangent line angle of er3 | 0.013 | 0.048 | 4.03E-01 |
|  | Tangent line angle of er4 | 0.046 | 0.048 | 1.51E-01 |
|  | Tangent line angle of er5 | 0.185 | 0.050 | 3.64E-05 |
|  | Tangent line angle of er6 | 0.080 | 0.049 | 5.34E-02 |
|  | Tangent line angle of er7 | 0.064 | 0.050 | 1.06E-01 |
|  | Tangent line angle of el1 | 0.137 | 0.052 | 4.06E-03 |
|  | Tangent line angle of el2 | 0.127 | 0.048 | 1.38E-03 |
|  | Tangent line angle of el3 | 0.081 | 0.049 | 3.32E-02 |
|  | Tangent line angle of el4 | 0.178 | 0.053 | 3.29E-04 |
|  | Tangent line angle of el5 | 0.120 | 0.050 | 7.72E-03 |
|  | Tangent line angle of el6 | 0.150 | 0.050 | 4.85E-04 |
|  | Tangent line angle of el7 | 0.086 | 0.049 | 3.29E-02 |
|  | Right eyelid peak position ratio | 0.000001 | 0.049 | 5.00E-01 |
|  | Left eyelid peak position ratio | 0.083 | 0.051 | 4.65E-02 |
|  | Right eyelid slant | 0.052 | 0.049 | 1.31E-01 |
|  | Left eyelid slant | 0.152 | 0.051 | 9.18E-04 |
|  | Right eyelid medial slant | 0.052 | 0.050 | 1.59E-01 |
|  | Left eyelid medial slant | 0.152 | 0.051 | 1.02E-03 |
|  | Right eyelid lateral slant | 0.111 | 0.051 | 1.46E-02 |
|  | Left eyelid lateral slant | 0.159 | 0.051 | 4.41E-04 |
|  | Right eyelid average curvature | 0.015 | 0.049 | 3.90E-01 |
|  | Right eyelid maximal curvature | 0.004 | 0.043 | 4.60E-01 |
|  | Left eyelid average curvature | 0.099 | 0.050 | 2.15E-02 |
|  | Left eyelid maximal curvature | 0.042 | 0.050 | 2.18E-01 |
