# Supplementary material for: Identification of five novel genetic loci related to facial morphology by genome-wide association studies
Source: BMC Genomics. 2018 Jun 19;19:481. doi: 10.1186/s12864-018-4865-9 (PMC6008943; doi:10.1186/s12864-018-4865-9)

Figure S1: Study design for facial morphology GWASs

We carried out a discovery GWAS for 85 facial traits in 5,643 Koreans (Phase 1) and follow-up analysis in 1,926 Koreans (Phase 2) to validate 128 candidate SNPs (discovery GWAS, $P < 5 \times 10^{-6}$). After the two-stage analysis, a meta-analysis (Phase 1+2) was performed.

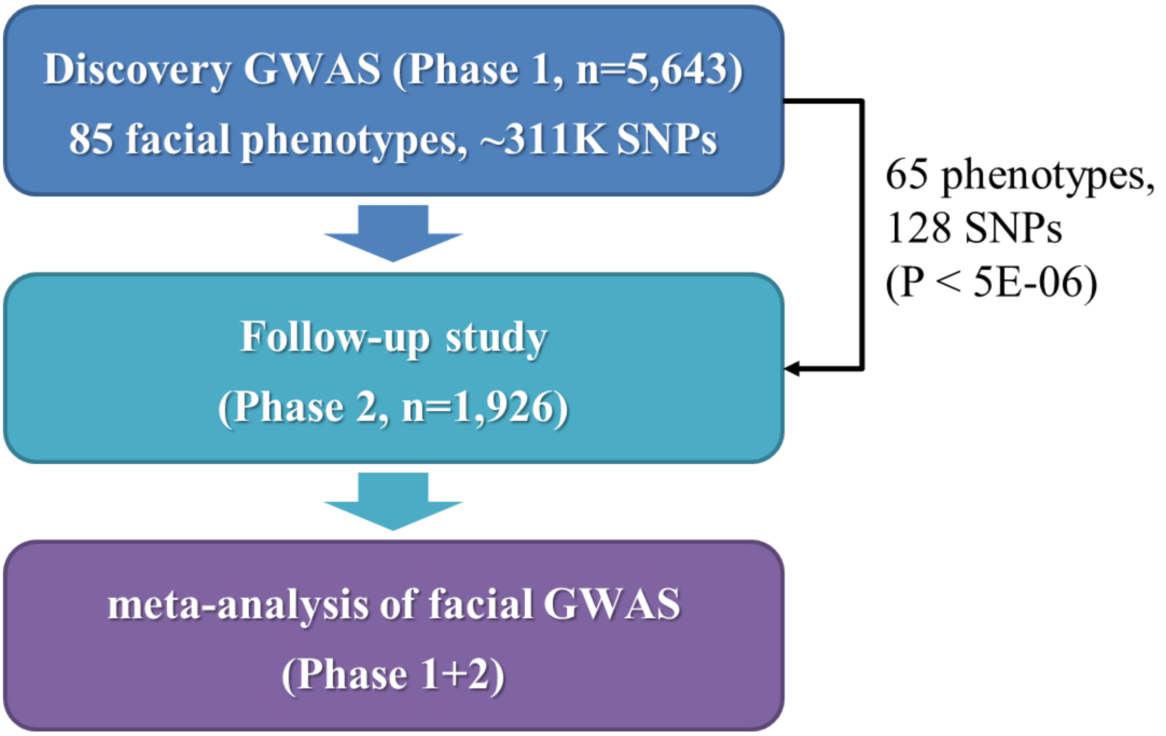

Supplement: Supplementary file 4 — Figure S1. Study design for facial morphology GWASs. (PDF 116 kb) [file 12864_2018_4865_MOESM4_ESM.pdf]
