# Supplementary material for: Identification of five novel genetic loci related to facial morphology by genome-wide association studies
Source: BMC Genomics. 2018 Jun 19;19:481. doi: 10.1186/s12864-018-4865-9 (PMC6008943; doi:10.1186/s12864-018-4865-9)
Supplement: Supplementary file 5 — Table S4. Characteristics of the study participants. (DOCX 32 kb) [file 12864_2018_4865_MOESM5_ESM.docx]

**Table S4. Characteristics of the study participants**

| **Variables** | **Discovery (Phase1)** | | | | | **Follow-up (Phase2)** | | | | | **Replication for *SOX9* locus^b^** | | | | |
| --- | --- | --- | --- | --- | --- | --- | --- | --- | --- | --- | --- | --- | --- | --- | --- |
|  | **Men (n = 2,648)** | | **Women (n = 2,995)** | | **P** | **Men (n = 687)** | | **Women (n = 1,239)** | | **P** | **Men (n = 587)** | | **Women (n = 1,353)** | | **P** |
|  | **Mean** | **SD** | **Mean** | **SD** |  | **Mean** | **SD** | **Mean** | **SD** |  | **Mean** | **SD** | **Mean** | **SD** |  |
| Age (years) | 59.86 | 8.40 | 60.85 | 8.57 | <5.0E-05 | 50.17 | 14.23 | 49.58 | 14.89 | 3.19E-01 | 40.71 | 12.675 | 43.608 | 11.11 | <5.0E-05 |
| BMI (kg/m^2^) | 24.25 | 2.93 | 24.61 | 3.19 | 1.048E-02 | 24.12 | 3.09 | 23.20 | 3.30 | <5.0E-05 | 24.45 | 3.017 | 22.963 | 3.08 | <5.0E-05 |
| Facial base width (mm) | 156.43 | 8.64 | 148.84 | 7.40 | <5.0E-05 | 156.36 | 9.11 | 148.28 | 7.52 | <5.0E-05 |  |  |  |  |  |
| Lower facial width (mm) | 138.08 | 9.75 | 130.45 | 7.98 | <5.0E-05 | 137.20 | 10.10 | 128.64 | 8.69 | <5.0E-05 |  |  |  |  |  |
| Upper facial width (mm) | 151.69 | 8.85 | 144.65 | 7.36 | <5.0E-05 | 152.16 | 9.03 | 144.95 | 7.57 | <5.0E-05 |  |  |  |  |  |
| Middle facial width (mm) | 152.61 | 8.90 | 144.76 | 7.22 | <5.0E-05 | 152.01 | 9.41 | 142.90 | 7.68 | <5.0E-05 |  |  |  |  |  |
| Upper lip height (mm) | 30.24 | 2.80 | 28.89 | 2.55 | <5.0E-05 | 29.63 | 2.98 | 28.01 | 2.62 | <5.0E-05 |  |  |  |  |  |
| Facial height (mm) | 77.85 | 4.86 | 73.21 | 4.56 | <5.0E-05 | 76.46 | 5.11 | 72.50 | 4.53 | <5.0E-05 |  |  |  |  |  |
| Upper facial area (mm^2^) | 7215.48 | 763.47 | 6367.63 | 643.46 | <5.0E-05 | 7074.16 | 830.56 | 6371.70 | 672.28 | <5.0E-05 |  |  |  |  |  |
| Lower facial area (mm^2^) | 4439.72 | 519.28 | 4012.17 | 443.19 | <5.0E-05 | 4342.24 | 563.43 | 3842.72 | 465.25 | <5.0E-05 |  |  |  |  |  |
| Facial width ratio of base to chin | 1.11 | 0.19 | 1.13 | 0.14 | <5.0E-05 | 1.14 | 0.06 | 1.15 | 0.06 | <5.0E-05 |  |  |  |  |  |
| Facial ratio of base width to height | 1.96 | 0.35 | 2.02 | 0.24 | <5.0E-05 | 2.05 | 0.15 | 2.05 | 0.13 | 9.25E-01 |  |  |  |  |  |
| Facial ratio of chin width to height | 1.74 | 0.32 | 1.77 | 0.24 | 6.43E-03 | 1.80 | 0.18 | 1.78 | 0.17 | 6.41E-03 |  |  |  |  |  |
| Right facial angle of en-ex-go (°) | 103.12 | 5.42 | 102.87 | 5.19 | 4.46E-02 | 101.02 | 6.24 | 99.81 | 6.08 | <5.0E-05 |  |  |  |  |  |
| Left facial angle of en-ex-go (°) | 104.11 | 5.44 | 103.46 | 5.29 | <5.0E-05 | 101.52 | 5.95 | 100.26 | 6.24 | <5.0E-05 |  |  |  |  |  |
| Right facial angle of ps-ex-go (°) | 123.61 | 6.17 | 125.53 | 5.77 | <5.0E-05 | 121.19 | 7.05 | 123.03 | 6.47 | <5.0E-05 |  |  |  |  |  |
| Left facial angle of ps-ex-go (°) | 125.16 | 6.00 | 126.48 | 5.70 | <5.0E-05 | 122.53 | 6.79 | 124.13 | 6.36 | <5.0E-05 |  |  |  |  |  |
| Right facial angle of en-ps-go (°) | 89.49 | 7.49 | 85.71 | 7.40 | <5.0E-05 | 87.63 | 7.76 | 82.28 | 8.05 | <5.0E-05 |  |  |  |  |  |
| Left facial angle of en-ps-go (°) | 89.70 | 7.46 | 85.85 | 7.66 | <5.0E-05 | 87.63 | 7.93 | 82.07 | 8.28 | <5.0E-05 |  |  |  |  |  |
| Forehead height (mm) | 51.69 | 10.02 | 44.32 | 7.97 | <5.0E-05 | 51.40 | 10.34 | 46.62 | 8.36 | <5.0E-05 |  |  |  |  |  |
| Lower forehead height (mm) | 28.08 | 9.62 | 23.91 | 7.14 | <5.0E-05 | 27.57 | 10.26 | 24.92 | 6.87 | <5.0E-05 |  |  |  |  |  |
| Upper forehead height (mm) | 22.62 | 8.35 | 20.17 | 6.74 | <5.0E-05 | 22.06 | 9.04 | 20.86 | 7.26 | 9.05E-03 |  |  |  |  |  |
| Brow ridge height (mm) | 29.72 | 4.33 | 29.04 | 3.88 | <5.0E-05 | 29.85 | 4.29 | 28.64 | 3.96 | <5.0E-05 |  |  |  |  |  |
| Upper forehead slant angle (°) | 50.42 | 14.43 | 55.95 | 15.38 | <5.0E-05 | 54.94 | 9.62 | 60.38 | 8.52 | <5.0E-05 |  |  |  |  |  |
| Brow ridge protrusion angle (°) | 88.56 | 5.71 | 87.77 | 5.21 | <5.0E-05 | 87.62 | 6.05 | 86.85 | 5.33 | 5.85E-03 |  |  |  |  |  |
| Upper forehead slant depth^a^ (mm) | 2.82 | 0.54 | 2.44 | 0.44 | <5.0E-05 | 2.72 | 0.58 | 2.41 | 0.49 | <5.0E-05 |  |  |  |  |  |
| Brow ridge protrusiona^a^ (mm) | 0.57 | 0.99 | 0.54 | 0.97 | 5.73E-02 | 0.68 | 1.04 | 0.58 | 0.98 | 1.62E-02 |  |  |  |  |  |
| Metopion position ratio | 0.53 | 0.11 | 0.52 | 0.10 | 7.90E-03 | 0.53 | 0.13 | 0.52 | 0.11 | 2.16E-03 |  |  |  |  |  |
| Metopion eminence depth^a^ (mm) | 1.19 | 0.82 | 1.04 | 0.63 | <5.0E-05 | 1.08 | 0.82 | 1.12 | 0.60 | 3.38E-01 |  |  |  |  |  |
| Intercanthal width (mm) | 35.62 | 3.80 | 35.77 | 3.49 | 1.79E-01 | 36.44 | 3.76 | 36.48 | 3.47 | 7.90E-01 |  |  |  |  |  |
| Outercanthal width (mm) | 99.03 | 6.46 | 95.03 | 5.91 | <5.0E-05 | 99.69 | 6.90 | 95.26 | 6.03 | <5.0E-05 |  |  |  |  |  |
| Right palpebral fissure height (mm) | 7.53 | 1.44 | 7.72 | 1.58 | <5.0E-05 | 7.74 | 1.40 | 8.46 | 1.63 | <5.0E-05 |  |  |  |  |  |
| Left palpebral fissure height (mm) | 7.73 | 1.47 | 7.82 | 1.62 | 3.16E-02 | 7.84 | 1.44 | 8.52 | 1.68 | <5.0E-05 |  |  |  |  |  |
| Right palpebrale fissure length (mm) | 31.90 | 3.67 | 29.87 | 3.60 | <5.0E-05 | 31.73 | 3.41 | 29.66 | 3.33 | <5.0E-05 |  |  |  |  |  |
| Left palpebrale fissure length (mm) | 31.44 | 3.86 | 29.42 | 3.62 | <5.0E-05 | 31.23 | 3.39 | 29.23 | 3.18 | <5.0E-05 |  |  |  |  |  |
| Right eye angle of ex-ps (°) | 21.03 | 4.91 | 23.23 | 4.74 | <5.0E-05 | 18.75 | 4.99 | 21.37 | 4.57 | <5.0E-05 |  |  |  |  |  |
| Left eye angle of ex-ps (°) | 21.64 | 4.90 | 23.52 | 4.58 | <5.0E-05 | 20.05 | 5.13 | 22.55 | 4.70 | <5.0E-05 |  |  |  |  |  |
| Right eye angle of en-ps (°) | 23.39 | 6.02 | 26.88 | 6.27 | <5.0E-05 | 25.87 | 6.06 | 30.20 | 6.08 | <5.0E-05 |  |  |  |  |  |
| Left eye angle of en-ps (°) | 23.79 | 6.18 | 27.17 | 6.47 | <5.0E-05 | 25.37 | 5.86 | 29.96 | 6.52 | <5.0E-05 |  |  |  |  |  |
| Right eye angle of en-ps-ex (°) | 134.49 | 14.99 | 129.38 | 11.96 | <5.0E-05 | 135.30 | 8.42 | 128.30 | 7.97 | <5.0E-05 |  |  |  |  |  |
| Left eye angle of en-ps-ex (°) | 134.73 | 8.60 | 129.41 | 9.00 | <5.0E-05 | 134.57 | 8.28 | 127.44 | 8.43 | <5.0E-05 |  |  |  |  |  |
| Eye tail length (mm) | 18.56 | 2.88 | 18.01 | 2.86 | <5.0E-05 | 18.74 | 2.82 | 18.11 | 2.79 | <5.0E-05 |  |  |  |  |  |
| Eye ratio of width to height | 0.24 | 0.05 | 0.26 | 0.05 | <5.0E-05 | 0.25 | 0.05 | 0.29 | 0.05 | <5.0E-05 |  |  |  |  |  |
| Ratio of eye width to base width | 0.42 | 0.06 | 0.41 | 0.06 | <5.0E-05 | 0.42 | 0.04 | 0.41 | 0.04 | 7.00E-05 |  |  |  |  |  |
| Subnasal width (mm) | 27.93 | 2.63 | 25.34 | 2.37 | <5.0E-05 | 27.14 | 2.86 | 24.67 | 2.60 | <5.0E-05 |  |  |  |  |  |
| Frontal nasal height (mm) | 47.68 | 3.62 | 44.31 | 3.29 | <5.0E-05 | 46.72 | 3.87 | 44.49 | 3.39 | <5.0E-05 |  |  |  |  |  |
| Profile nasal length^a^ (mm) | 3.93 | 0.08 | 3.85 | 0.08 | <5.0E-05 | 3.94 | 0.08 | 3.86 | 0.08 | <5.0E-05 |  |  |  |  |  |
| Nasal bridge height (mm) | 36.08 | 3.92 | 32.93 | 3.59 | <5.0E-05 | 35.92 | 4.02 | 33.32 | 3.71 | <5.0E-05 |  |  |  |  |  |
| Nasal tip height^a^ (mm) | 2.63 | 0.14 | 2.56 | 0.13 | <5.0E-05 | 2.66 | 0.16 | 2.59 | 0.14 | <5.0E-05 |  |  |  |  |  |
| Nasal bridge depth (mm) | 22.97 | 3.45 | 19.70 | 3.11 | <5.0E-05 | 22.67 | 3.63 | 19.21 | 3.07 | <5.0E-05 | 22.30 | 3.038 | 19.211 | 2.71 | <5.0E-05 |
| Nasal tip protrusion^a^ (mm) | 2.56 | 0.17 | 2.44 | 0.18 | <5.0E-05 | 2.59 | 0.16 | 2.52 | 0.17 | <5.0E-05 | 2.63 | 0.137 | 2.556 | 0.13 | <5.0E-05 |
| Profile nasal area^a^ (mm^2^) | 5.98 | 0.17 | 5.76 | 0.17 | <5.0E-05 | 5.99 | 0.18 | 5.82 | 0.17 | <5.0E-05 | 6.01 | 0.139 | 5.842 | 0.14 | <5.0E-05 |
| Nasal bridge angle (°) | 56.34 | 9.47 | 55.88 | 14.18 | <5.0E-05 | 57.89 | 4.97 | 60.06 | 4.75 | <5.0E-05 |  |  |  |  |  |
| Nasolabial angle^a^ (°) | 3.84 | 0.13 | 3.88 | 0.13 | <5.0E-05 | 3.86 | 0.15 | 3.86 | 0.14 | 8.52E-01 | 3.80 | 0.115 | 3.816 | 0.11 | 8.47E-03 |
| Profile nasal angle^a^ (°) | 4.50 | 0.81 | 4.35 | 1.19 | <5.0E-05 | 4.65 | 0.06 | 4.68 | 0.05 | <5.0E-05 | 4.64 | 0.043 | 4.661 | 0.05 | <5.0E-05 |
| Right upper lip thickness^a^ (mm) | 1.93 | 0.56 | 2.11 | 0.36 | <5.0E-05 | 2.12 | 0.24 | 2.16 | 0.20 | 1.89E-03 |  |  |  |  |  |
| Left upper lip thickness^a^ (mm) | 1.90 | 0.55 | 2.09 | 0.34 | <5.0E-05 | 2.11 | 0.23 | 2.16 | 0.19 | 7.37E-05 |  |  |  |  |  |
| Right eyelid peak width (cm) | 1.37 | 0.25 | 1.33 | 0.23 | <5.0E-05 | 1.36 | 0.24 | 1.36 | 0.23 | 8.04E-01 |  |  |  |  |  |
| Left eyelid peak width (cm) | 1.36 | 0.25 | 1.34 | 0.23 | 4.58E-03 | 1.36 | 0.24 | 1.35 | 0.22 | 9.49E-01 |  |  |  |  |  |
| Right eyelid width (cm) | 3.57 | 0.34 | 3.41 | 0.33 | <5.0E-05 | 3.45 | 0.33 | 3.29 | 0.33 | <5.0E-05 |  |  |  |  |  |
| Left eyelid width (cm) | 3.57 | 0.35 | 3.43 | 0.34 | <5.0E-05 | 3.45 | 0.32 | 3.30 | 0.34 | <5.0E-05 |  |  |  |  |  |
| Tangent line angle of er1 (°) | -0.80 | 0.21 | -0.84 | 0.21 | <5.0E-05 | -0.87 | 0.19 | -0.92 | 0.18 | <5.0E-05 |  |  |  |  |  |
| Tangent line angle of er2 (°) | -0.49 | 0.11 | -0.53 | 0.11 | <5.0E-05 | -0.50 | 0.12 | -0.57 | 0.12 | <5.0E-05 |  |  |  |  |  |
| Tangent line angle of er3 (°) | -0.10 | 0.15 | -0.12 | 0.15 | <5.0E-05 | -0.11 | 0.14 | -0.16 | 0.14 | <5.0E-05 |  |  |  |  |  |
| Tangent line angle of er4 (°) | 0.20 | 0.13 | 0.21 | 0.14 | 1.57E-01 | 0.16 | 0.13 | 0.15 | 0.14 | 4.08E-01 |  |  |  |  |  |
| Tangent line angle of er5 (°) | 0.36 | 0.13 | 0.38 | 0.13 | <5.0E-05 | 0.31 | 0.14 | 0.34 | 0.14 | <5.0E-05 |  |  |  |  |  |
| Tangent line angle of er6 (°) | 0.39 | 0.13 | 0.43 | 0.14 | <5.0E-05 | 0.40 | 0.13 | 0.45 | 0.13 | <5.0E-05 |  |  |  |  |  |
| Tangent line angle of er7 (°) | 0.24 | 0.44 | 0.30 | 0.45 | <5.0E-05 | 0.44 | 0.38 | 0.50 | 0.38 | 5.11E-04 |  |  |  |  |  |
| Tangent line angle of el1 (°) | -0.78 | 0.24 | -0.82 | 0.24 | <5.0E-05 | -0.86 | 0.20 | -0.91 | 0.19 | <5.0E-05 |  |  |  |  |  |
| Tangent line angle of el2 (°) | -0.49 | 0.11 | -0.53 | 0.12 | <5.0E-05 | -0.51 | 0.11 | -0.58 | 0.12 | <5.0E-05 |  |  |  |  |  |
| Tangent line angle of el3 (°) | -0.09 | 0.15 | -0.12 | 0.15 | <5.0E-05 | -0.11 | 0.14 | -0.16 | 0.15 | <5.0E-05 |  |  |  |  |  |
| Tangent line angle of el4 (°) | 0.22 | 0.14 | 0.21 | 0.14 | 2.76E-01 | 0.16 | 0.13 | 0.16 | 0.14 | 5.97E-01 |  |  |  |  |  |
| Tangent line angle of el5 (°) | 0.39 | 0.13 | 0.40 | 0.14 | <5.0E-05 | 0.31 | 0.13 | 0.35 | 0.14 | <5.0E-05 |  |  |  |  |  |
| Tangent line angle of el6 (°) | 0.42 | 0.13 | 0.44 | 0.14 | <5.0E-05 | 0.40 | 0.13 | 0.46 | 0.13 | <5.0E-05 |  |  |  |  |  |
| Tangent line angle of el7 (°) | 0.25 | 0.44 | 0.29 | 0.47 | 1.27E-04 | 0.43 | 0.38 | 0.47 | 0.40 | 3.15E-03 |  |  |  |  |  |
| Right eyelid peak position ratio | 0.38 | 0.07 | 0.39 | 0.07 | 3.10E-04 | 0.39 | 0.07 | 0.41 | 0.08 | <5.0E-05 |  |  |  |  |  |
| Left eyelid peak position ratio | 0.38 | 0.07 | 0.39 | 0.07 | <5.0E-05 | 0.39 | 0.08 | 0.41 | 0.07 | <5.0E-05 |  |  |  |  |  |
| Right eyelid slant | 0.01 | 0.07 | 0.01 | 0.07 | 2.83E-01 | -0.01 | 0.08 | -0.02 | 0.08 | 1.57E-03 |  |  |  |  |  |
| Left eyelid slant | 0.02 | 0.07 | 0.02 | 0.07 | <5.0E-05 | -0.01 | 0.07 | -0.02 | 0.07 | 3.64E-02 |  |  |  |  |  |
| Right eyelid medial slant | -0.51 | 0.14 | -0.55 | 0.14 | <5.0E-05 | -0.53 | 0.14 | -0.58 | 0.15 | <5.0E-05 |  |  |  |  |  |
| Left eyelid medial slant | -0.51 | 0.15 | -0.55 | 0.15 | <5.0E-05 | -0.53 | 0.14 | -0.59 | 0.15 | <5.0E-05 |  |  |  |  |  |
| Right eyelid lateral slant | 0.33 | 0.10 | 0.36 | 0.10 | <5.0E-05 | 0.33 | 0.10 | 0.38 | 0.11 | <5.0E-05 |  |  |  |  |  |
| Left eyelid lateral slant | 0.35 | 0.10 | 0.38 | 0.10 | <5.0E-05 | 0.34 | 0.11 | 0.39 | 0.11 | <5.0E-05 |  |  |  |  |  |
| Right eyelid average curvature^a^ (cm) | -4.05 | 0.29 | -3.92 | 0.28 | <5.0E-05 | -3.97 | 0.33 | -3.84 | 0.32 | <5.0E-05 |  |  |  |  |  |
| Right eyelid maximal curvature^a^ (cm) | -3.12 | 0.48 | -3.02 | 0.47 | <5.0E-05 | -3.00 | 0.50 | -2.95 | 0.52 | 2.69E-02 |  |  |  |  |  |
| Left eyelid average curvature^a^ (cm) | -3.99 | 0.49 | -3.89 | 0.41 | <5.0E-05 | -3.98 | 0.30 | -3.83 | 0.31 | <5.0E-05 |  |  |  |  |  |
| Left eyelid maximal curvature^a^ (cm) | -3.12 | 0.52 | -3.02 | 0.49 | <5.0E-05 | -3.01 | 0.49 | -2.94 | 0.51 | 1.60E-02 |  |  |  |  |  |

Data are presented as mean and standard deviation (SD). BMI, body mass index.

^a^ ln-transformed

^b^This population was used for replicating associations between five nose traits (nasal bridge depth, nasal tip protrusion, profile nasal area, nasolabial angle, and profile nasal angle) and *SOX9* SNPs.
