# Supplementary material for: Identification of five novel genetic loci related to facial morphology by genome-wide association studies
Source: BMC Genomics. 2018 Jun 19;19:481. doi: 10.1186/s12864-018-4865-9 (PMC6008943; doi:10.1186/s12864-018-4865-9)

Figure S2: Q-Q plots for the discovery GWAS (85 facial traits)

[Face shape – width]

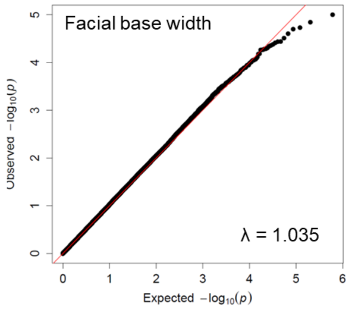

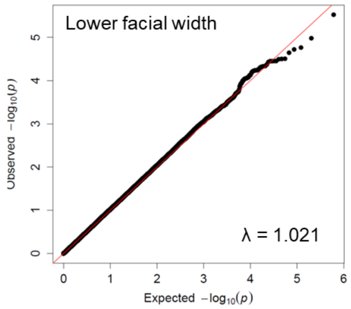

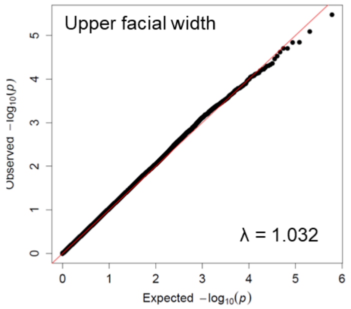

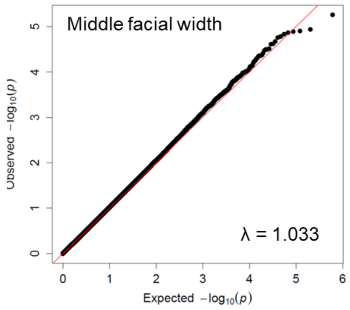

[Face shape – height]

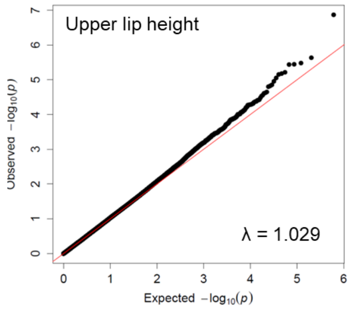

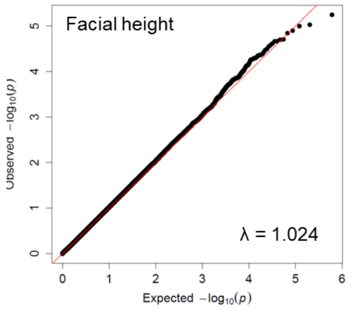

[Face shape – area]

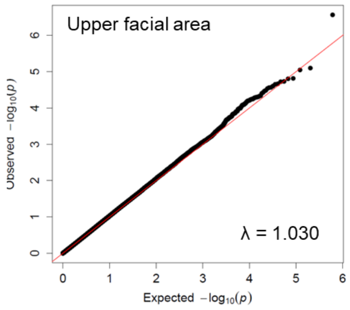

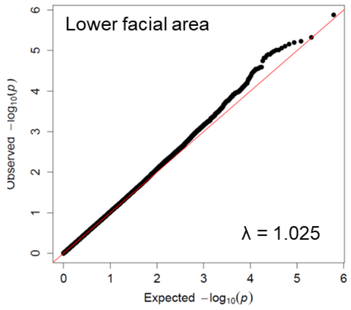

[Face shape – ratio]

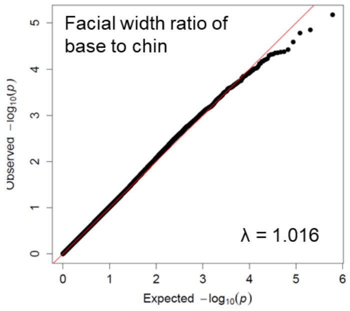

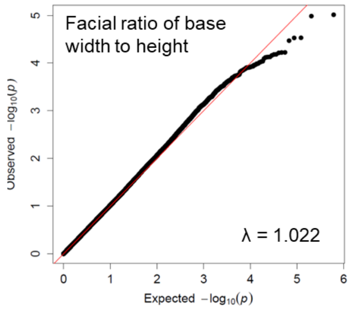

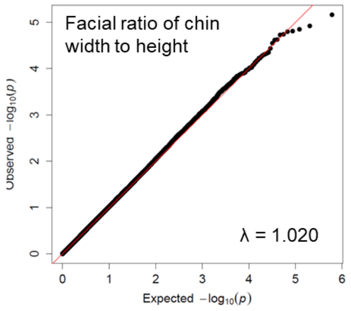

[Face shape – angle]

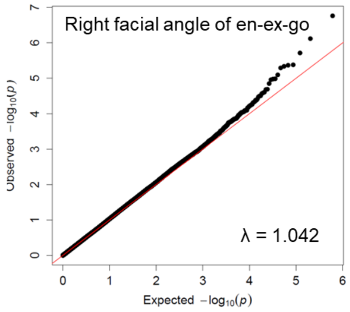

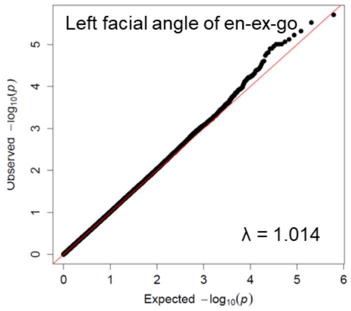

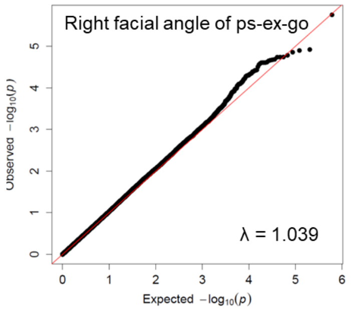

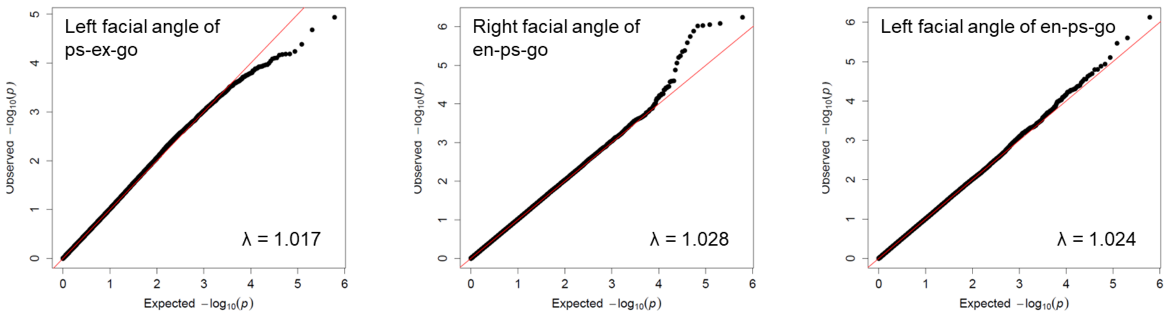

[Forehead – height]

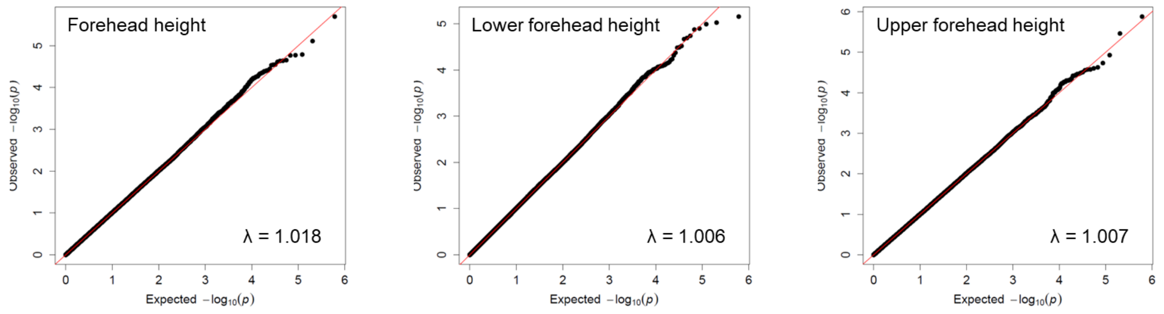

[Forehead – depth]

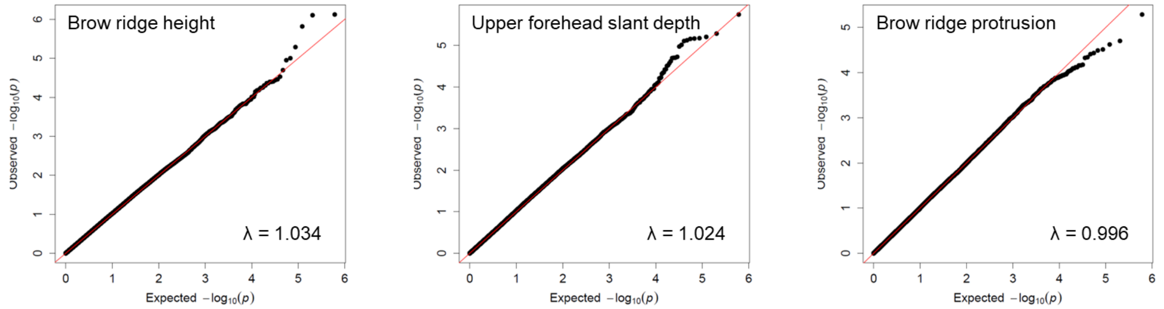

[Forehead – angle]

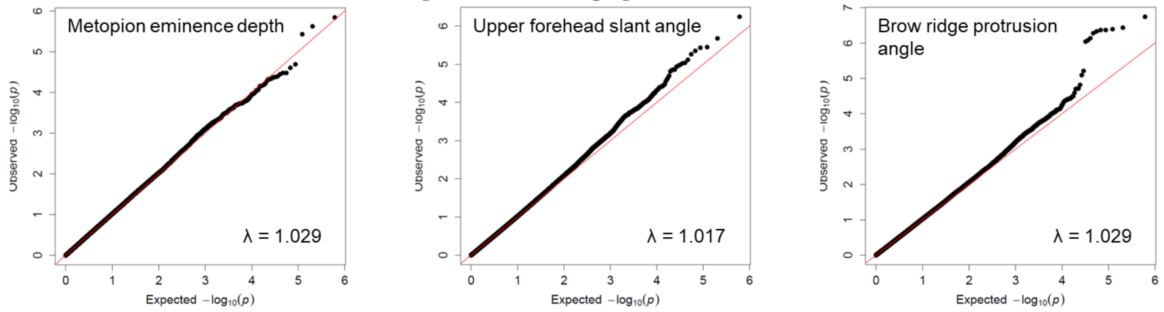

[Forehead – ratio]

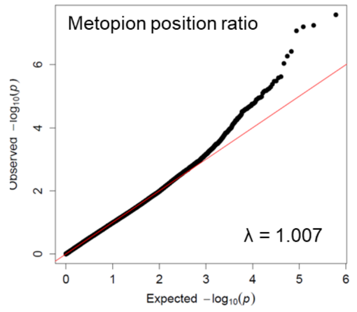

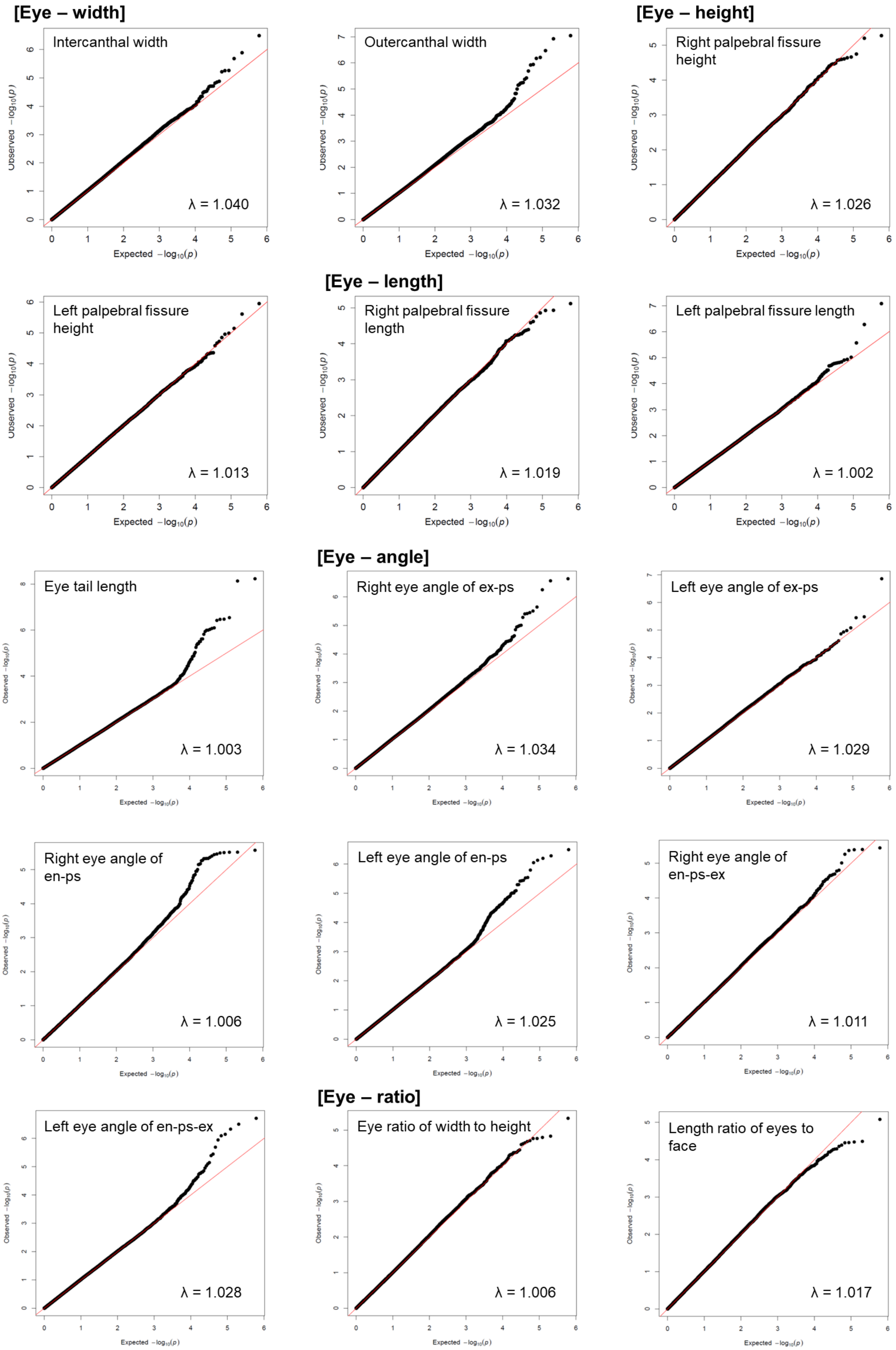

[Eye – width]
Intercanthal width
λ = 1.040
Outercanthal width
λ = 1.032
[Eye – height]
Right palpebral fissure height
λ = 1.026
Left palpebral fissure height
λ = 1.013
[Eye – length]
Right palpebral fissure length
λ = 1.019
Left palpebral fissure length
λ = 1.002
Eye tail length
λ = 1.003
[Eye – angle]
Right eye angle of ex-ps
λ = 1.034
Left eye angle of ex-ps
λ = 1.029
Right eye angle of en-ps
λ = 1.006
Left eye angle of en-ps
λ = 1.025
Right eye angle of en-ps-ex
λ = 1.011
Left eye angle of en-ps-ex
λ = 1.028
[Eye – ratio]
Eye ratio of width to height
λ = 1.006
Length ratio of eyes to face
λ = 1.017
Expected $-\log_{10}(p)$
Observed $-\log_{10}(p)$

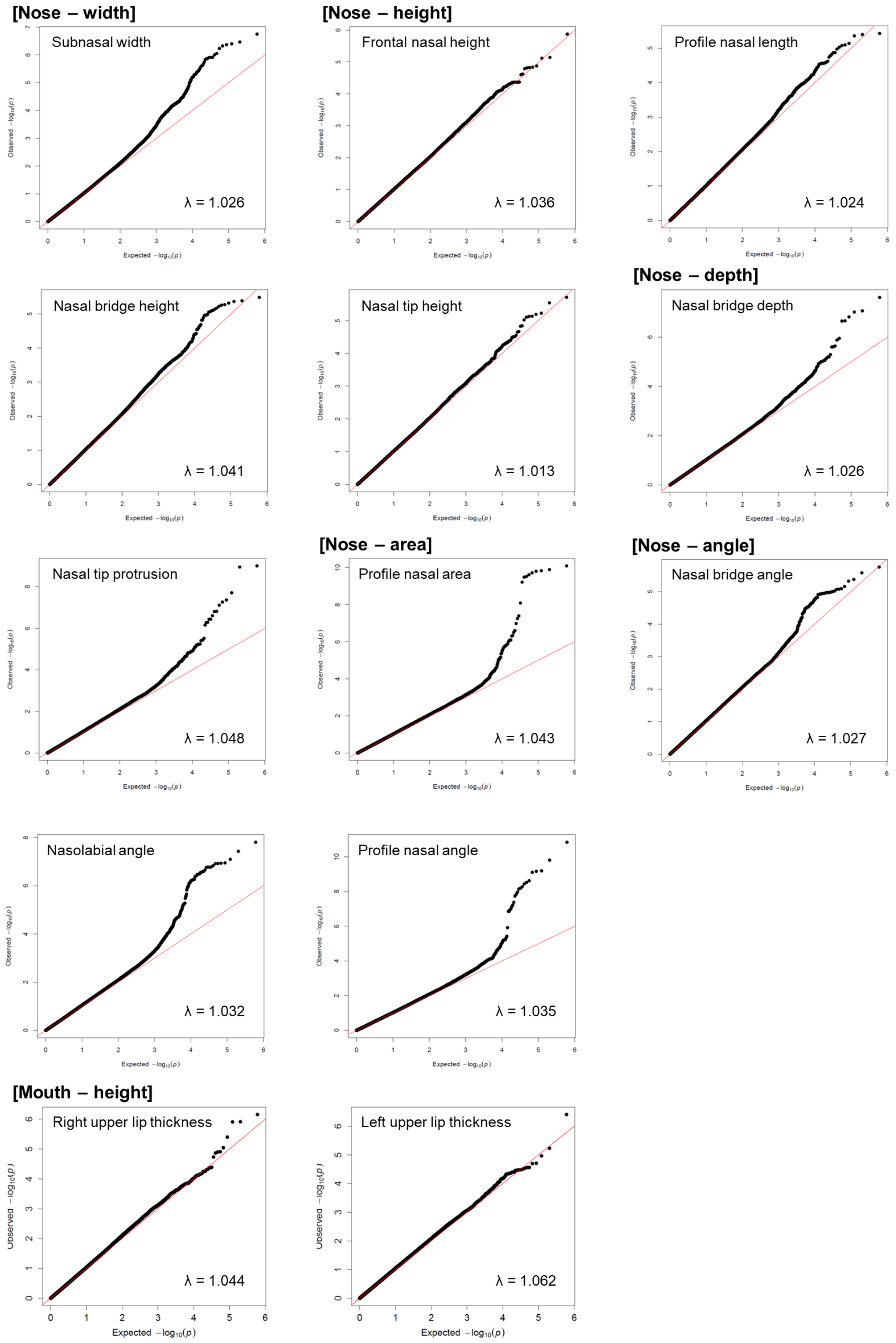
[Nose – width]
Subnasal width
λ = 1.026
[Nose – height]
Frontal nasal height
λ = 1.036
Profile nasal length
λ = 1.024
Nasal bridge height
λ = 1.041
Nasal tip height
λ = 1.013
[Nose – depth]
Nasal bridge depth
λ = 1.026
Nasal tip protrusion
λ = 1.048
[Nose – area]
Profile nasal area
λ = 1.043
[Nose – angle]
Nasal bridge angle
λ = 1.027
Nasolabial angle
λ = 1.032
Profile nasal angle
λ = 1.035
[Mouth – height]
Right upper lip thickness
λ = 1.044
Left upper lip thickness
λ = 1.062
Observed −log10(p)
Expected −log10(p)

[Upper eyelid – width]

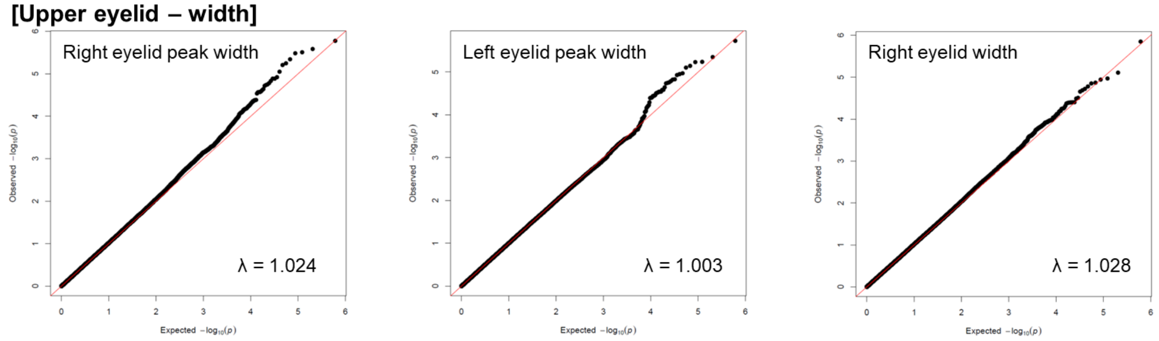

[Upper eyelid – angle]

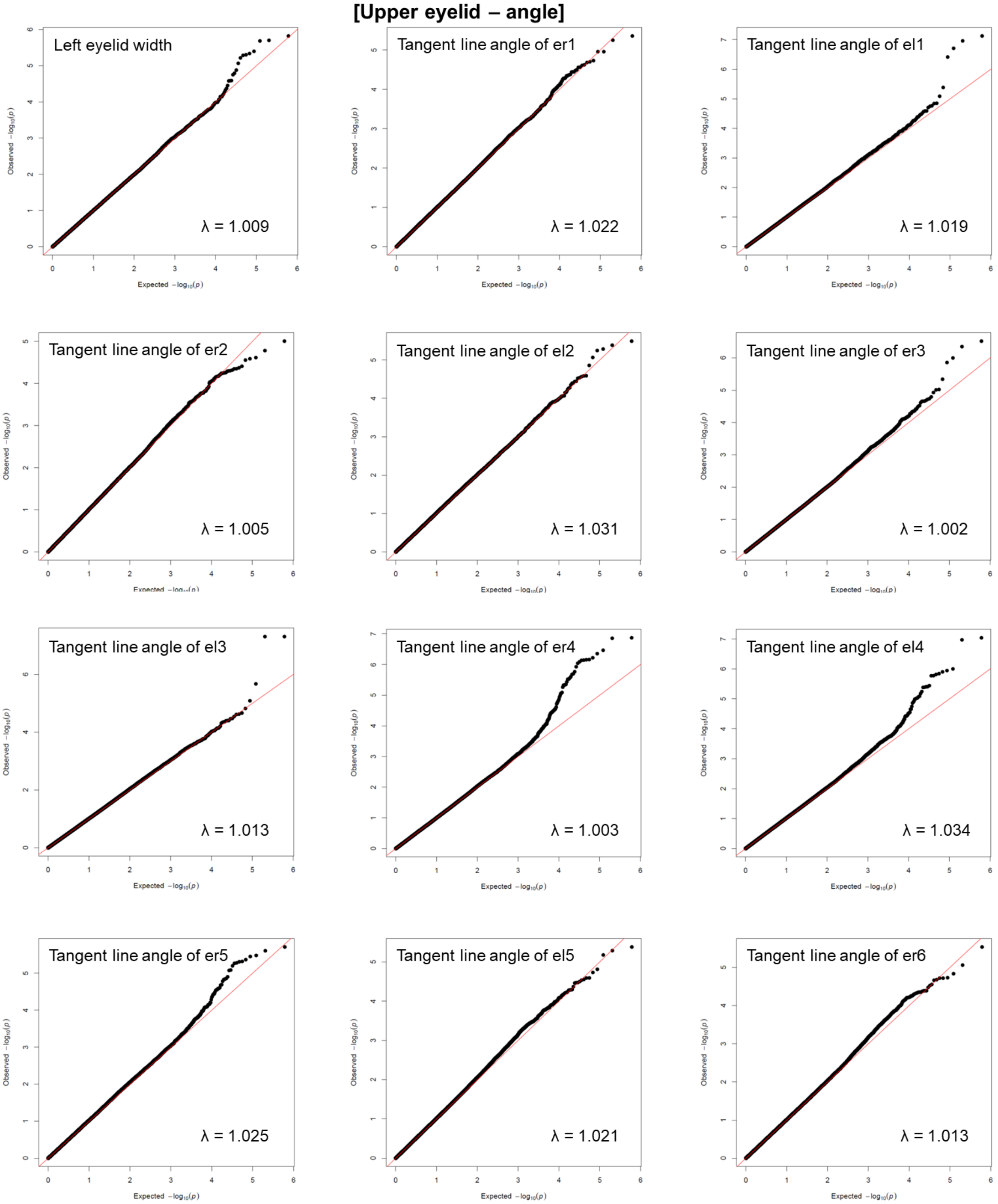

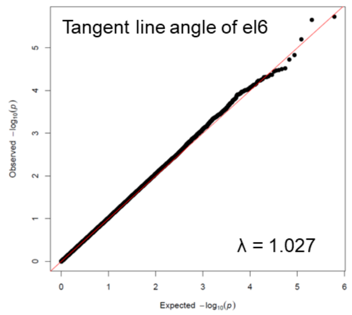

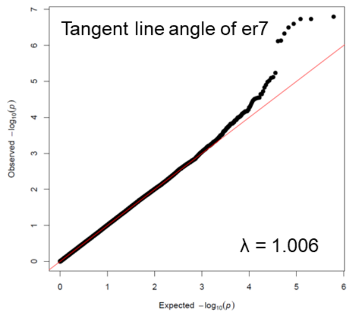

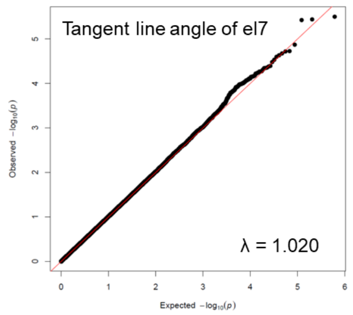

[Upper eyelid – ratio]

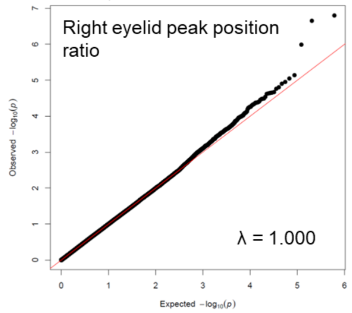

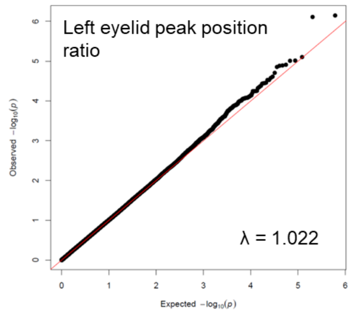

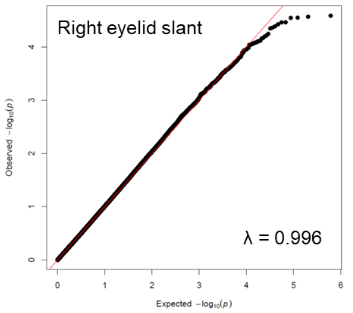

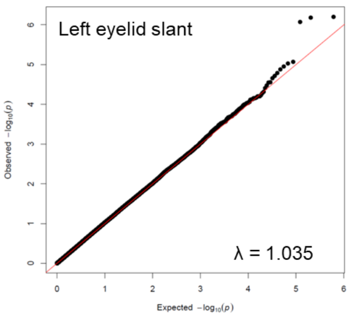

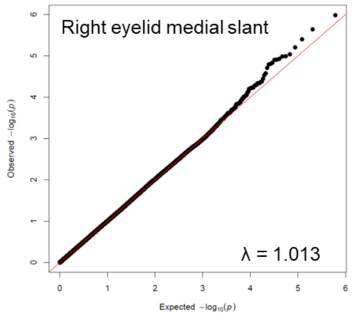

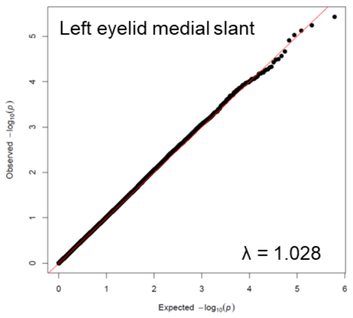

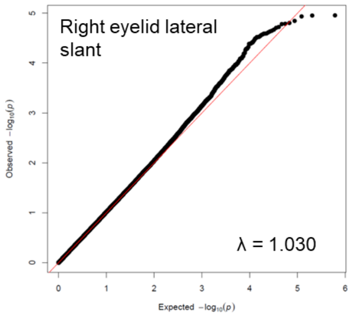

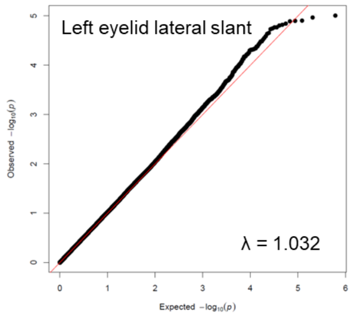

[Upper eyelid – curvature]

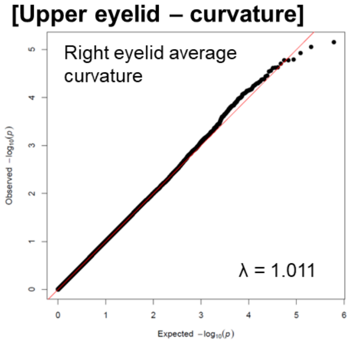

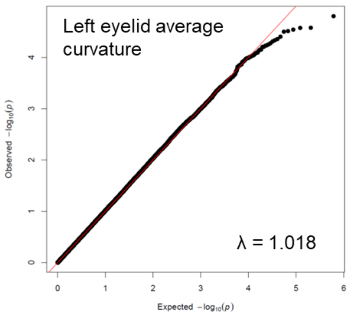

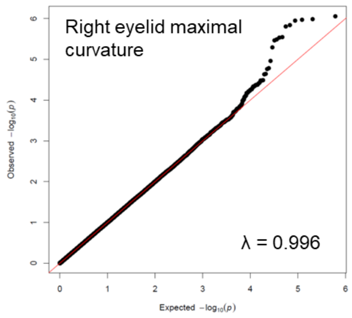

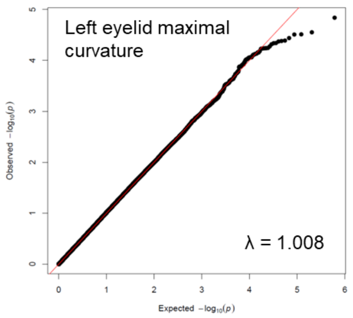

Supplement: Supplementary file 6 — Figure S2. Q-Q plots for the discovery GWAS (85 facial traits). (PDF 1945 kb) [file 12864_2018_4865_MOESM6_ESM.pdf]
