# Supplementary material for: Identification of five novel genetic loci related to facial morphology by genome-wide association studies
Source: BMC Genomics. 2018 Jun 19;19:481. doi: 10.1186/s12864-018-4865-9 (PMC6008943; doi:10.1186/s12864-018-4865-9)
Supplement: Supplementary file 7 — Figure S3. Manhattan plots for the discovery GWAS (85 facial traits). (PDF 2645 kb) [file 12864_2018_4865_MOESM7_ESM.pdf]

Figure S3: Manhattan plots for the discovery GWAS (85 facial traits)

-$\log_{10}(P)$ values are plotted against chromosomal base-pair positions. Green labels indicate significant loci showing associations with each facial trait in the discovery GWAS (Phase 1). The red line represents the genome-wide significance level (P = 5 × 10^{-8}), and the blue line represents the suggestive P value (P = 5 × 10^{-6}).

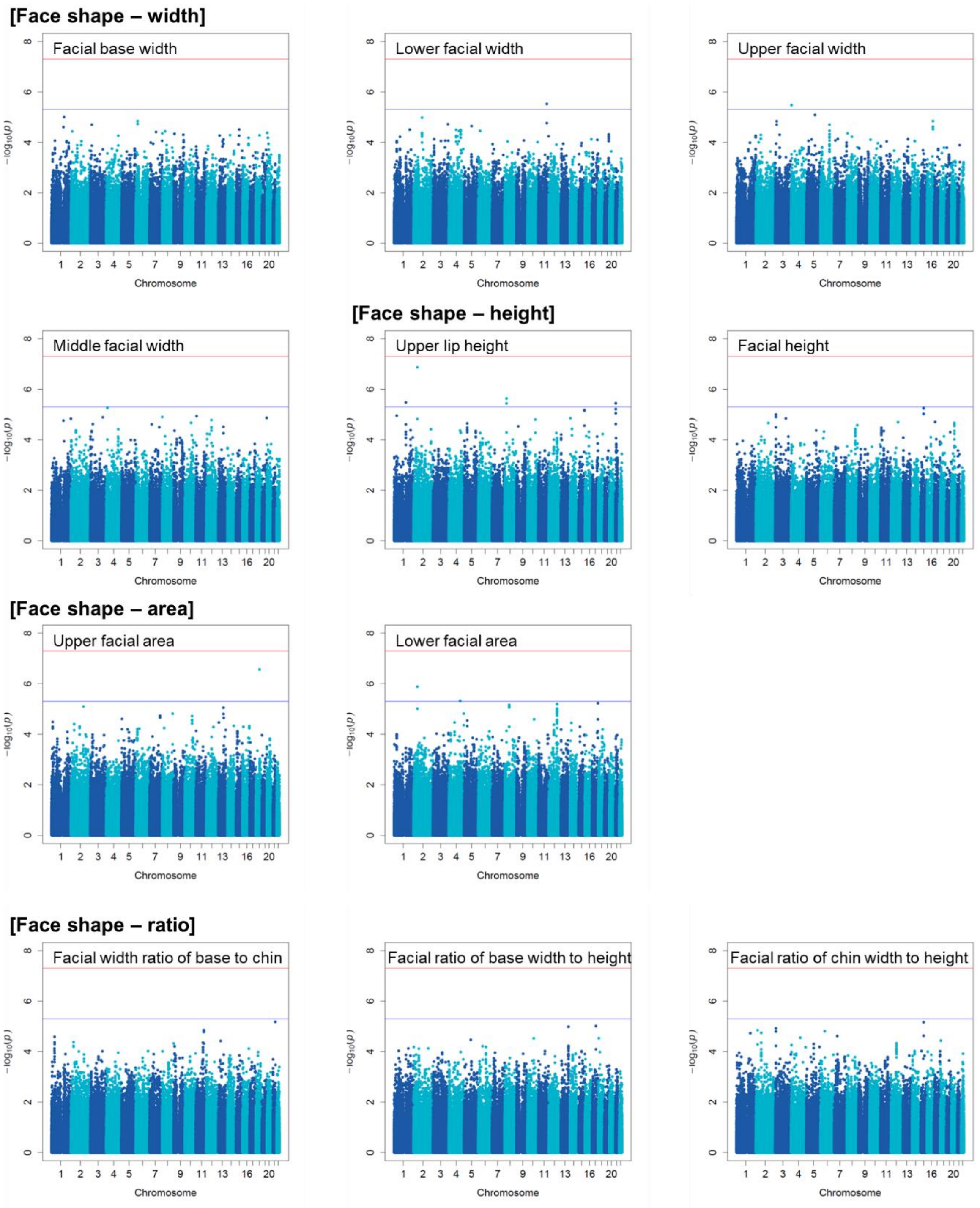

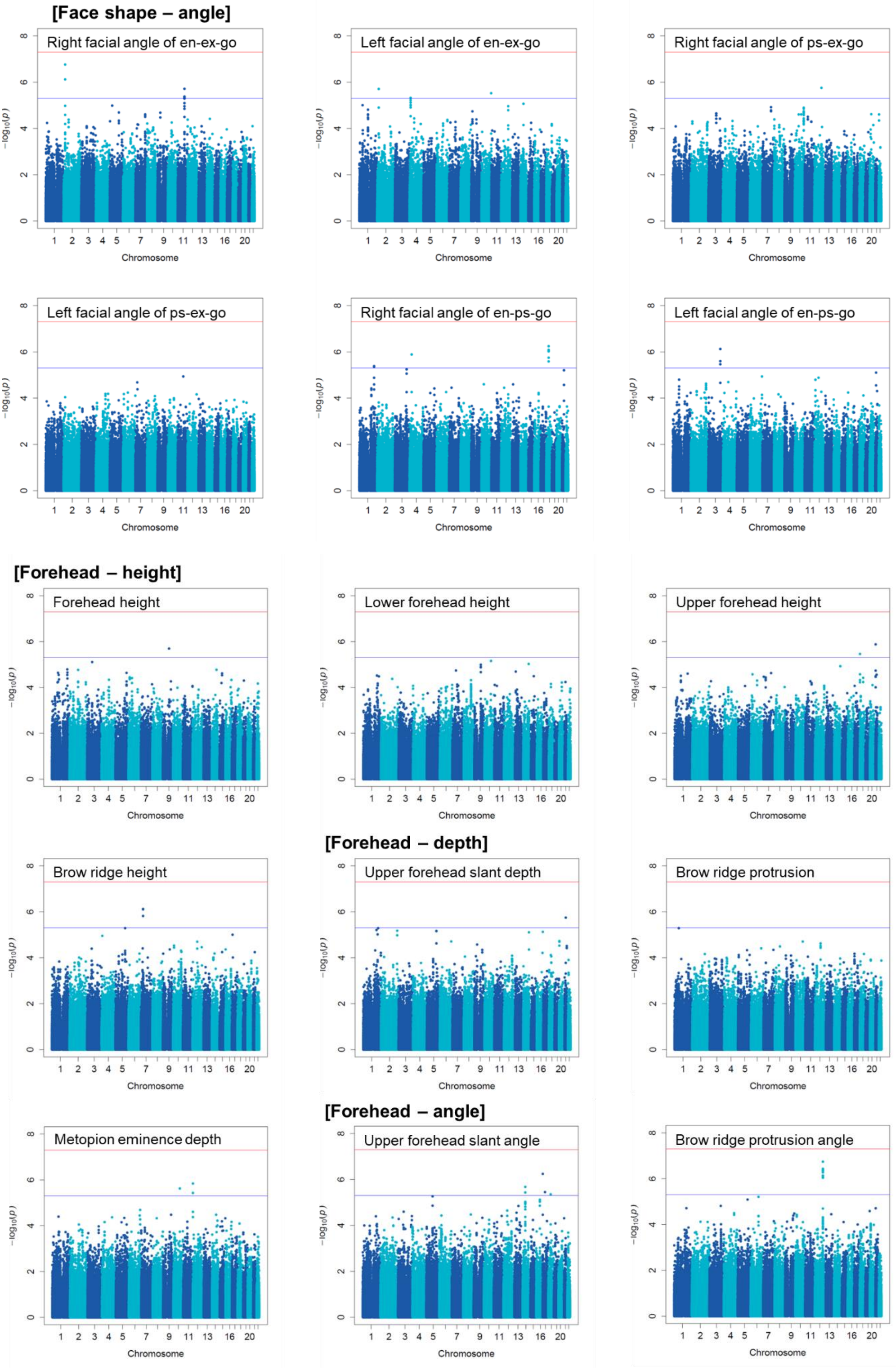
[Face shape – angle]
Right facial angle of en-ex-go
Left facial angle of en-ex-go
Right facial angle of ps-ex-go
Left facial angle of ps-ex-go
Right facial angle of en-ps-go
Left facial angle of en-ps-go
[Forehead – height]
Forehead height
Lower forehead height
Upper forehead height
Brow ridge height
[Forehead – depth]
Upper forehead slant depth
Brow ridge protrusion
Metopion eminence depth
[Forehead – angle]
Upper forehead slant angle
Brow ridge protrusion angle
Chromosome
$-\log_{10}(p)$

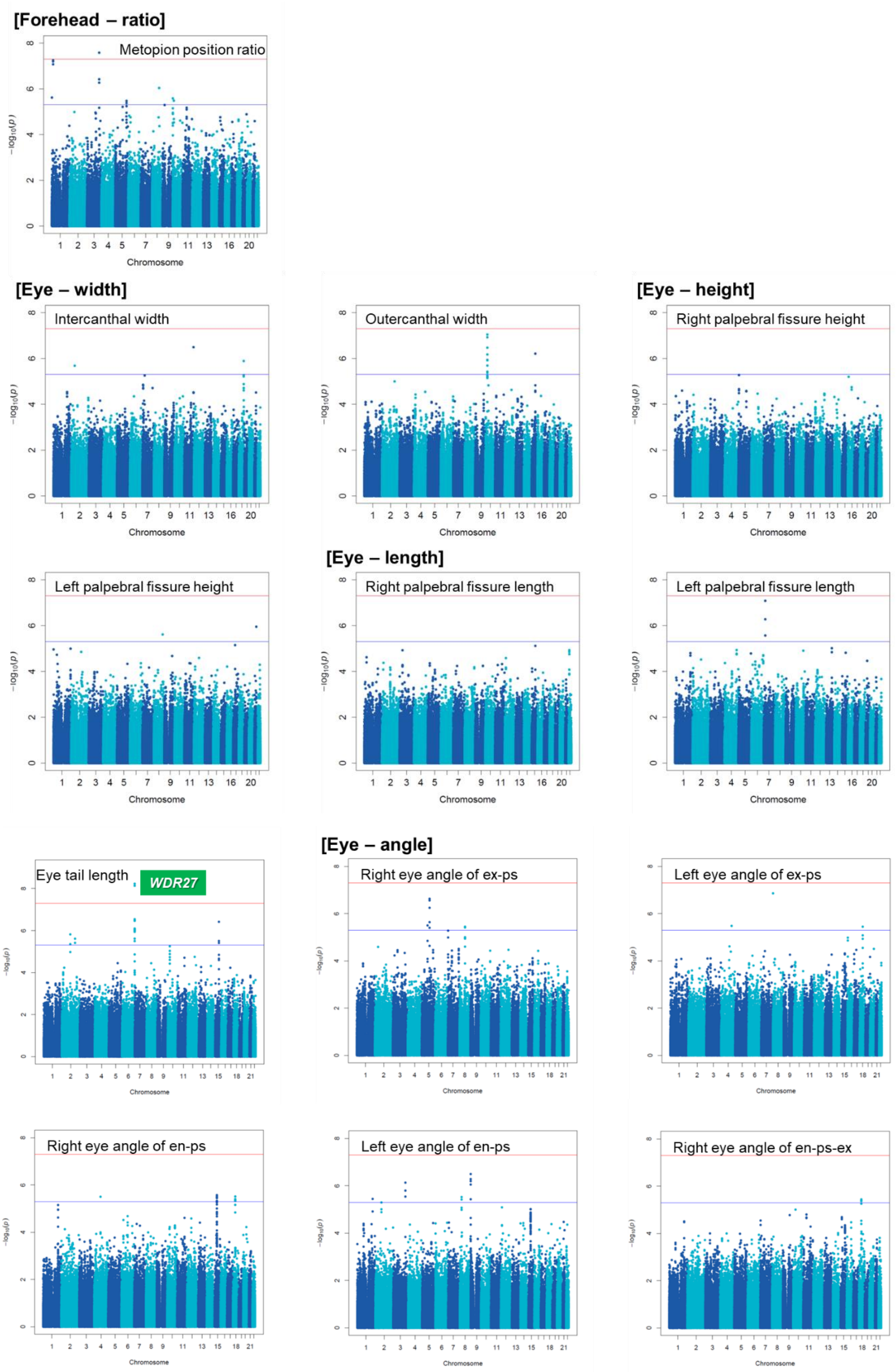
[Forehead – ratio]
Metopion position ratio
[Eye – width]
Intercanthal width
Outercanthal width
[Eye – height]
Right palpebral fissure height
Left palpebral fissure height
[Eye – length]
Right palpebral fissure length
Left palpebral fissure length
Eye tail length
WDR27
[Eye – angle]
Right eye angle of ex-ps
Left eye angle of ex-ps
Right eye angle of en-ps
Left eye angle of en-ps
Right eye angle of en-ps-ex
Chromosome

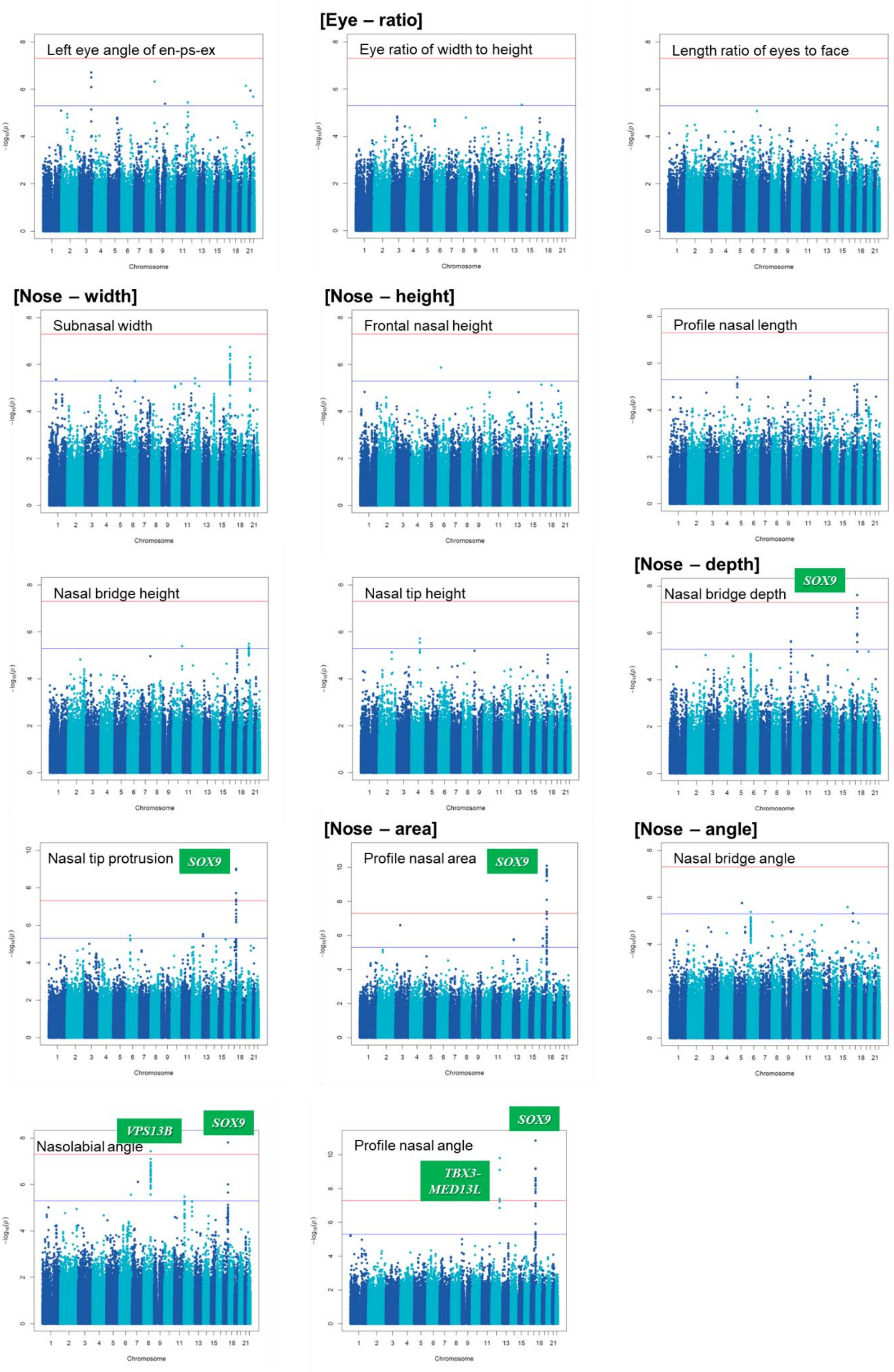
[Eye – ratio]
Left eye angle of en-ps-ex
Eye ratio of width to height
Length ratio of eyes to face
[Nose – width]
Subnasal width
[Nose – height]
Frontal nasal height
Profile nasal length
Nasal bridge height
Nasal tip height
[Nose – depth]
SOX9
Nasal bridge depth
Nasal tip protrusion
SOX9
[Nose – area]
Profile nasal area
SOX9
[Nose – angle]
Nasal bridge angle
VPS13B
SOX9
Nasolabial angle
SOX9
Profile nasal angle
TBX3-MED13L
Chromosome

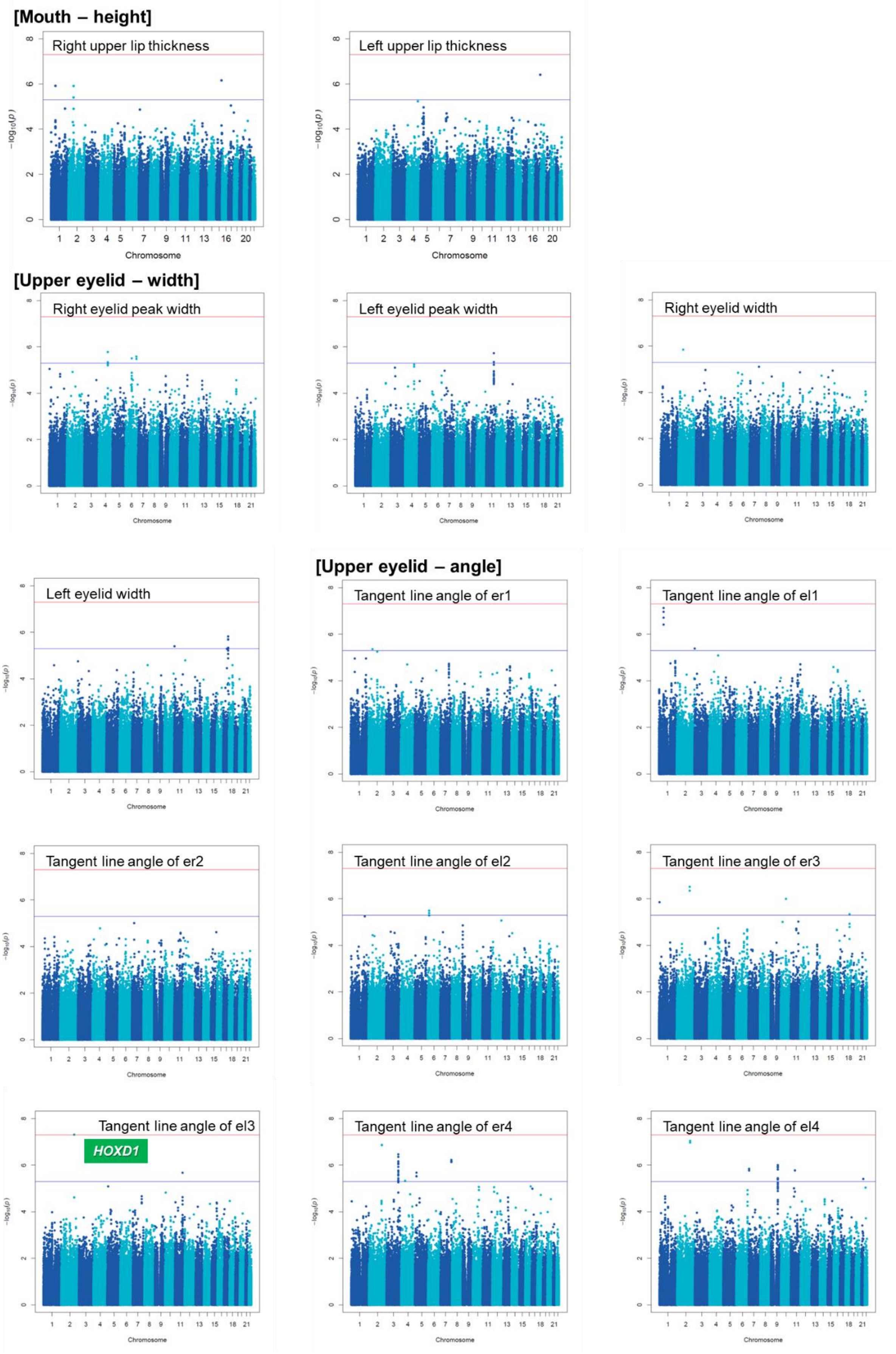
[Mouth – height]
Right upper lip thickness
Left upper lip thickness
[Upper eyelid – width]
Right eyelid peak width
Left eyelid peak width
Right eyelid width
Left eyelid width
[Upper eyelid – angle]
Tangent line angle of er1
Tangent line angle of el1
Tangent line angle of er2
Tangent line angle of el2
Tangent line angle of er3
Tangent line angle of el3
HOXD1
Tangent line angle of er4
Tangent line angle of el4
Chromosome

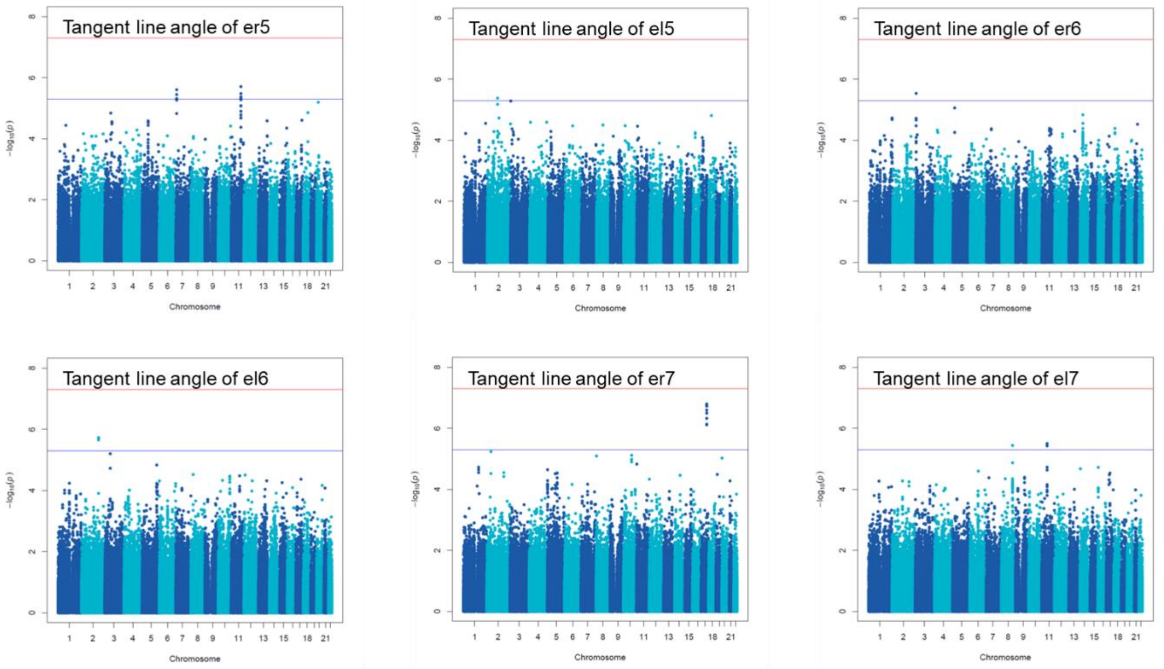

[Upper eyelid – ratio]

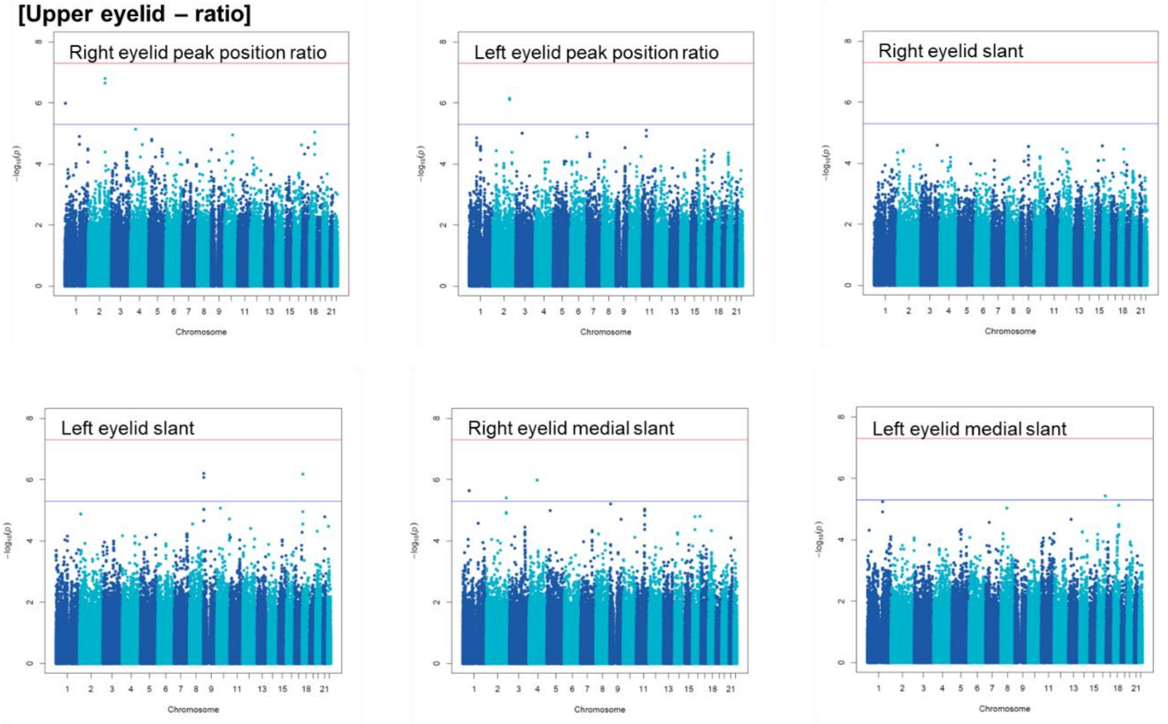

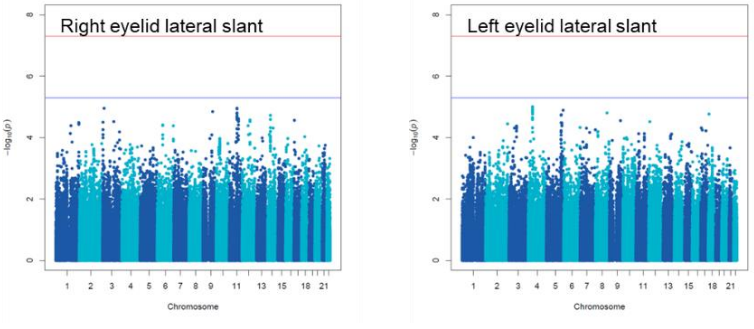

[Upper eyelid – curvature]

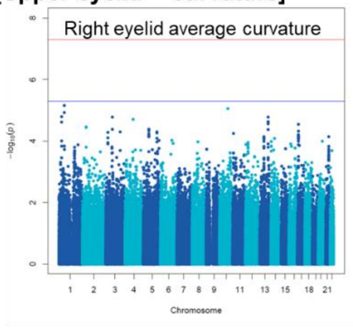

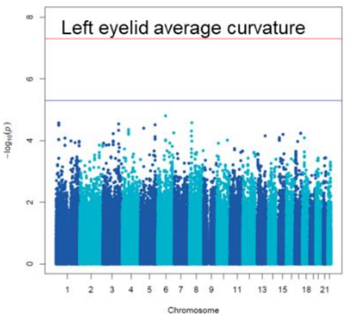
Left eyelid average curvature
$-\log_{10}(p)$
Chromosome

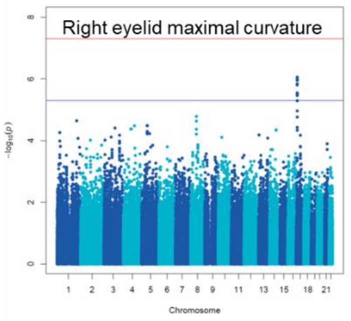
Right eyelid maximal curvature
$-\log_{10}(p)$
Chromosome

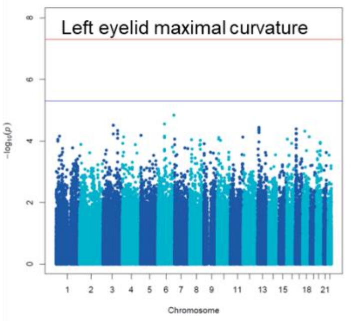
Left eyelid maximal curvature
$-\log_{10}(p)$
Chromosome
