# Supplementary material for: Identification of five novel genetic loci related to facial morphology by genome-wide association studies
Source: BMC Genomics. 2018 Jun 19;19:481. doi: 10.1186/s12864-018-4865-9 (PMC6008943; doi:10.1186/s12864-018-4865-9)
Supplement: Supplementary file 8 — Table S5. Association results in Phase 1, Phase 2, and the Phase 1 + 2 meta-analysis (63 phenotypes, 117 SNPs). (DOCX 78 kb) [file 12864_2018_4865_MOESM8_ESM.docx]

**Table S5.** Association results in Phase 1, Phase 2, and the Phase 1+2 meta-analysis (63 phenotypes, 117 SNPs)

| **Facial Traits** |  | **SNP** | **CHR** | **Position**  **(bp)^a^** | **Gene^b^** | **coded allele** | **Non-coded allele** | **Phase 1 (n=5,643)** | | |  | **Phase 2 (n=1,926)** | | |  | **Meta-analysis (Phase 1+2)** | | | |
| --- | --- | --- | --- | --- | --- | --- | --- | --- | --- | --- | --- | --- | --- | --- | --- | --- | --- | --- | --- |
|  |  |  |  |  |  |  |  | **AF** | **beta ± se** | ***P*-value** |  | **AF** | **beta ± se** | ***P*-value** |  | **beta** | ***P*-value** | ***Q*** | ***I^2^*** |
| ***Face shape*** |  |  |  |  |  |  |  |  |  |  |  |  |  |  |  |  |  |  |  |
| Angle | Left facial angle of en-ps-go | rs551016 | 3 | 162,972,574 | *OTOL1* | G | A | 0.40 | -0.681 ± 0.137 | 7.49E-07 |  | 0.41 | 0.072 ± 0.244 | 7.67E-01 |  | -0.500 | 3.01E-05 | 0.01 | 86.17 |
|  | Right facial angle of en-ps-go | rs1980050 | 1 | 202,713,311 | *PIK3C2B* | T | C | 0.19 | 0.784 ± 0.170 | 4.13E-06 |  | 0.20 | 0.548 ± 0.294 | 6.26E-02 |  | 0.725 | 8.55E-07 | 0.49 | 0.00 |
|  |  | rs11722052 | 4 | 29,387,746 | intergenic | C | T | 0.15 | -0.914 ± 0.189 | 1.30E-06 |  | 0.15 | 0.447 ± 0.333 | 1.80E-01 |  | -0.584 | 3.78E-04 | 0.00 | 92.10 |
|  |  | rs10502642 | 18 | 30,986,872 | *MAPRE2*  *-ZNF397* | A | T | 0.19 | -0.856 ± 0.171 | 5.68E-07 |  | 0.18 | -0.285 ± 0.303 | 3.48E-01 |  | -0.718 | 1.42E-06 | 0.10 | 62.91 |
|  | Left facial angle of en-ex-go | rs7567283 | 2 | 19,595,772 | *OSR1-WDR35* | G | A | 0.24 | -0.525 ± 0.110 | 1.96E-06 |  | 0.24 | -0.524 ± 0.208 | 1.16E-02 |  | -0.525 | 7.05E-08 | 1.00 | 0.00 |
|  |  | rs3899794 | 4 | 14,773,927 | *CPEB2-CC2D2A* | A | G | 0.19 | -0.542 ± 0.118 | 4.78E-06 |  | 0.19 | 0.220 ± 0.229 | 3.36E-01 |  | -0.381 | 2.91E-04 | 0.00 | 88.59 |
|  |  | rs6482814 | 10 | 132,295,907 | intergenic | C | T | 0.45 | -0.443 ± 0.095 | 2.99E-06 |  | 0.45 | -0.251 ± 0.184 | 1.72E-01 |  | -0.403 | 1.73E-06 | 0.35 | 0.00 |
|  | Right facial angle of en-ex-go | rs7567283 | 2 | 19,595,772 | *OSR1-WDR35* | G | A | 0.24 | -0.572 ± 0.109 | 1.72E-07 |  | 0.24 | -0.410 ± 0.205 | 4.59E-02 |  | -0.536 | **2.75E-08** | 0.48 | 0.00 |
|  |  | rs3847535 | 11 | 92,197,672 | *FAT3* | A | G | 0.19 | -0.575 ± 0.121 | 1.93E-06 |  | 0.17 | -0.089 ± 0.239 | 7.09E-01 |  | -0.476 | 9.85E-06 | 0.07 | 69.78 |
|  | Right facial angle of ps-ex-go | rs10860276 | 12 | 96,976,282 | *MIR4303*  *-SLC9A7P1* | G | A | 0.33 | -0.575 ± 0.120 | 1.76E-06 |  | 0.33 | -0.233 ± 0.219 | 2.88E-01 |  | -0.496 | 2.54E-06 | 0.17 | 46.54 |
| Area | Lower facial area | rs2881324 | 2 | 46,430,398 | *EPAS1* | G | T | 0.10 | 67.900 ± 14.020 | 1.31E-06 |  | 0.11 | 46.270 ± 24.920 | 6.35E-02 |  | 62.700 | 2.88E-07 | 0.45 | 0.00 |
|  |  | rs2303414 | 4 | 142,047,919 | *RNF150* | T | C | 0.39 | 40.080 ± 8.748 | 4.71E-06 |  | 0.39 | 5.531 ± 16.050 | 7.30E-01 |  | 32.167 | 2.82E-05 | 0.06 | 72.01 |
| Height | Upper lip height | rs2881324 | 2 | 46,430,398 | *EPAS1* | G | T | 0.10 | 0.438 ± 0.083 | 1.35E-07 |  | 0.11 | 0.109 ± 0.142 | 4.46E-01 |  | 0.355 | 7.62E-07 | 0.05 | 74.98 |
|  |  | rs750022 | 8 | 23,688,687 | *SLC25A37-STC1* | G | T | 0.18 | -0.314 ± 0.066 | 2.32E-06 |  | 0.15 | -0.015 ± 0.126 | 9.03E-01 |  | -0.249 | 2.27E-05 | 0.04 | 77.37 |
|  |  | rs2824582 | 21 | 18,261,874 | *CHODL* | G | A | 0.31 | 0.257 ± 0.055 | 3.58E-06 |  | 0.32 | -0.088 ± 0.095 | 3.56E-01 |  | 0.169 | 3.96E-04 | 0.00 | 89.83 |
| Width | Lower facial width | rs3741060 | 11 | 107,885,191 | *EXPH5* | G | A | 0.21 | -0.786 ± 0.168 | 2.98E-06 |  | 0.22 | 0.126 ± 0.317 | 6.92E-01 |  | -0.586 | 7.90E-05 | 0.01 | 84.48 |
|  | Upper facial width | rs1838973 | 4 | 5,208,163 | *STK32B* | A | G | 0.08 | -1.180 ± 0.254 | 3.34E-06 |  | 0.08 | -0.118 ± 0.454 | 7.96E-01 |  | -0.927 | 2.81E-05 | 0.04 | 76.04 |
| ***Forehead*** |  |  |  |  |  |  |  |  |  |  |  |  |  |  |  |  |  |  |  |
| Angle | Upper forehead slant angle | rs12895072 | 14 | 55,238,252 | *KTN1* | T | C | 0.22 | -0.961 ± 0.202 | 2.10E-06 |  | 0.24 | -0.008 ± 0.346 | 9.81E-01 |  | -0.718 | 3.91E-05 | 0.02 | 82.27 |
|  |  | rs216215 | 17 | 2,087,418 | *SMG6* | T | C | 0.42 | -0.863 ± 0.172 | 5.71E-07 |  | 0.43 | -0.308 ± 0.297 | 3.00E-01 |  | -0.723 | 1.23E-06 | 0.11 | 61.79 |
|  |  | rs4795157 | 17 | 32,305,940 | *MRM1-LHX1* | G | T | 0.15 | 1.124 ± 0.242 | 3.55E-06 |  | 0.15 | -0.185 ± 0.413 | 6.55E-01 |  | 0.789 | 1.60E-04 | 0.01 | 86.60 |
|  |  | rs17747476 | 18 | 32,435,285 | *FHOD3* | T | C | 0.18 | -1.016 ± 0.221 | 4.45E-06 |  | 0.18 | -0.125 ± 0.387 | 7.48E-01 |  | -0.797 | 3.33E-05 | 0.05 | 75.01 |
| Depth | Upper forehead slant depth | rs363444 | 21 | 29,908,319 | *GRIK1* | A | G | 0.19 | 0.056 ± 0.012 | 1.80E-06 |  | 0.20 | 0.029 ± 0.022 | 1.85E-01 |  | 0.050 | 1.30E-06 | 0.28 | 14.97 |
|  | Metopion eminence depth | rs3758477 | 10 | 89,253,352 | *LINC00864*  *-MINPP1* | T | G | 0.35 | 0.068 ± 0.014 | 2.39E-06 |  | 0.35 | 0.031 ± 0.024 | 1.87E-01 |  | 0.058 | 2.34E-06 | 0.19 | 42.56 |
|  |  | rs4283041 | 12 | 1,508,174 | *ERC1*  *-FBXL14* | T | C | 0.17 | -0.089 ± 0.018 | 1.44E-06 |  | 0.17 | -0.030 ± 0.030 | 3.07E-01 |  | -0.073 | 3.45E-06 | 0.10 | 63.86 |
| Height | Upper forehead height | rs2017823 | 18 | 2,373,523 | *METTL4* | G | A | 0.26 | -0.757 ± 0.163 | 3.45E-06 |  | 0.25 | 0.009 ± 0.306 | 9.76E-01 |  | -0.588 | 4.33E-05 | 0.03 | 79.50 |
|  | Forehead height | rs10081631 | 9 | 83,580,994 | *TLE1*  *-SPATA31D4* | C | T | 0.45 | 0.824 ± 0.173 | 2.02E-06 |  | 0.44 | 0.218 ± 0.305 | 4.75E-01 |  | 0.676 | 7.22E-06 | 0.08 | 66.54 |
|  | Brow ridge height | rs323187 | 7 | 29,117,232 | *CPVL* | G | A | 0.42 | -0.395 ± 0.080 | 7.54E-07 |  | 0.41 | -0.092 ± 0.140 | 5.11E-01 |  | -0.320 | 3.74E-06 | 0.06 | 71.94 |
| Ratio | Metopion position ratio | rs10909872 | 1 | 2,713,412 | *MMEL1*  *-ACTRT2* | C | A | 0.09 | -0.109 ± 0.023 | 2.41E-06 |  | 0.09 | -0.002 ± 0.007 | 8.22E-01 |  | -0.011 | 1.05E-01 | 0.00 | 94.93 |
|  |  | rs3790774 | 1 | 19,543,034 | *CAPZB* | A | G | 0.12 | -0.108 ± 0.020 | 5.66E-08 |  | 0.13 | 0.000 ± 0.006 | 9.35E-01 |  | -0.009 | 1.04E-01 | 0.00 | 96.29 |
|  |  | rs157668 | 5 | 156,046,667 | *SGCD* | C | G | 0.15 | -0.086 ± 0.019 | 3.36E-06 |  | 0.14 | 0.007 ± 0.006 | 2.08E-01 |  | -0.001 | 8.71E-01 | 0.00 | 95.69 |
|  |  | rs920200 | 8 | 96,971,052 | *C8orf37*  *-AS1-GDF6* | C | A | 0.13 | -0.096 ± 0.020 | 9.14E-07 |  | 0.13 | -0.014 ± 0.006 | 2.16E-02 |  | -0.020 | 2.94E-04 | 0.00 | 93.88 |
|  |  | rs816598 | 10 | 537,658 | *DIP2C* | C | A | 0.07 | -0.120 ± 0.026 | 2.61E-06 |  | 0.08 | 0.006 ± 0.008 | 4.15E-01 |  | -0.004 | 5.81E-01 | 0.00 | 95.55 |
|  |  | rs7080073 | 10 | 14,505,116 | *FRMD4A*  *-FAM107B* | G | T | 0.09 | -0.110 ± 0.024 | 3.29E-06 |  | 0.09 | -0.003 ± 0.007 | 6.86E-01 |  | -0.012 | 7.92E-02 | 0.00 | 94.67 |
| ***Eye*** |  |  |  |  |  |  |  |  |  |  |  |  |  |  |  |  |  |  |  |
| Angle | Left eye angle of en-ps | rs551016 | 3 | 162,972,574 | *OTOL1* | G | A | 0.40 | 0.578 ± 0.117 | 7.40E-07 |  | 0.41 | -0.160 ± 0.191 | 4.05E-01 |  | 0.378 | 1.47E-04 | 0.00 | 90.77 |
|  |  | rs2936 | 8 | 23,758,165 | *SLC25A37*  *-STC1* | T | C | 0.13 | -0.783 ± 0.167 | 2.98E-06 |  | 0.14 | 0.169 ± 0.274 | 5.37E-01 |  | -0.524 | 2.42E-04 | 0.00 | 88.61 |
|  |  | rs4742094 | 9 | 550,077 | *KANK1* | G | A | 0.27 | 0.655 ± 0.128 | 3.19E-07 |  | 0.28 | 0.136 ± 0.215 | 5.25E-01 |  | 0.519 | 2.35E-06 | 0.04 | 76.78 |
|  | Right eye angle of en-ps | rs17010857 | 4 | 86,915,002 | *ARHGAP24* | G | T | 0.41 | 0.526 ± 0.113 | 3.13E-06 |  | 0.40 | -0.076 ± 0.185 | 6.83E-01 |  | 0.363 | 1.59E-04 | 0.01 | 86.99 |
|  |  | rs2570228 | 15 | 49,964,204 | *TMOD3* | C | T | 0.48 | -0.518 ± 0.111 | 3.05E-06 |  | 0.48 | 0.182 ± 0.182 | 3.18E-01 |  | -0.328 | 5.23E-04 | 0.00 | 90.74 |
|  |  | rs10502642 | 18 | 30,986,872 | *MAPRE2*  *-ZNF397* | A | T | 0.19 | 0.669 ± 0.143 | 3.07E-06 |  | 0.18 | -0.053 ± 0.236 | 8.22E-01 |  | 0.475 | 1.06E-04 | 0.01 | 85.39 |
|  | Left eye angle of ex-ps | rs901686 | 4 | 141,997,657 | *TBC1D9*  *-RNF150* | C | A | 0.23 | 0.488 ± 0.105 | 3.30E-06 |  | 0.23 | 0.042 ± 0.183 | 8.17E-01 |  | 0.377 | 3.30E-05 | 0.03 | 77.68 |
|  |  | rs2972180 | 8 | 1,669,140 | *DLGAP2*  *-CLN8* | T | C | 0.18 | 0.606 ± 0.115 | 1.38E-07 |  | 0.16 | 0.325 ± 0.213 | 1.28E-01 |  | 0.543 | 8.12E-08 | 0.25 | 25.63 |
|  |  | rs12457805 | 18 | 40,853,235 | *SETBP1* | G | A | 0.43 | 0.411 ± 0.088 | 3.57E-06 |  | 0.43 | 0.083 ± 0.152 | 5.85E-01 |  | 0.328 | 1.81E-05 | 0.06 | 71.01 |
|  | Right eye angle of ex-ps | rs10447178 | 5 | 77,224,047 | *TBCA-AP3B1* | G | A | 0.39 | -0.435 ± 0.093 | 3.15E-06 |  | 0.39 | -0.082 ± 0.155 | 5.98E-01 |  | -0.341 | 1.94E-05 | 0.05 | 73.61 |
|  | Left eye angle of en-ps-ex | rs551016 | 3 | 162,972,574 | *OTOL1* | G | A | 0.40 | -0.859 ± 0.165 | 1.96E-07 |  | 0.41 | 0.372 ± 0.269 | 1.67E-01 |  | -0.523 | 2.01E-04 | 0.00 | 93.44 |
|  |  | rs6994270 | 8 | 119,417,676 | *SAMD12* | G | C | 0.15 | -1.159 ± 0.230 | 4.72E-07 |  | 0.15 | -0.580 ± 0.386 | 1.33E-01 |  | -1.007 | 3.39E-07 | 0.20 | 39.95 |
|  |  | rs2676622 | 9 | 114,494,325 | *KIAA1958*  *-SNX30* | A | C | 0.16 | -1.015 ± 0.220 | 4.08E-06 |  | 0.16 | 0.159 ± 0.354 | 6.54E-01 |  | -0.688 | 2.31E-04 | 0.00 | 87.38 |
|  |  | rs7970587 | 12 | 14,466,726 | *ATF7IP* | G | A | 0.38 | 0.771 ± 0.166 | 3.59E-06 |  | 0.40 | 0.117 ± 0.265 | 6.58E-01 |  | 0.586 | 3.12E-05 | 0.04 | 77.06 |
|  |  | rs6016745 | 20 | 40,383,597 | *PTPRT* | A | T | 0.19 | -1.014 ± 0.204 | 7.20E-07 |  | 0.17 | -0.173 ± 0.353 | 6.24E-01 |  | -0.803 | 5.58E-06 | 0.04 | 76.45 |
|  |  | rs17231256 | 21 | 39,370,759 | *ETS2*  *-PSMG1* | T | C | 0.13 | 1.186 ± 0.244 | 1.14E-06 |  | 0.13 | 0.105 ± 0.391 | 7.89E-01 |  | 0.884 | 1.90E-05 | 0.02 | 81.85 |
|  |  | rs16989337 | 22 | 30,355,055 | *PISD* | T | A | 0.06 | 1.579 ± 0.332 | 2.06E-06 |  | 0.06 | -0.864 ± 0.574 | 1.32E-01 |  | 0.966 | 7.78E-04 | 0.00 | 92.63 |
|  | Right eye angle of en-ps-ex | rs10502642 | 18 | 30,986,872 | *MAPRE2*  *-ZNF397* | A | T | 0.19 | -0.950 ± 0.206 | 4.09E-06 |  | 0.18 | -0.050 ± 0.335 | 8.82E-01 |  | -0.703 | 6.20E-05 | 0.02 | 80.92 |
| Length | Left palpebrale fissure length | rs6461561 | 7 | 21,441,135 | *SP4* | G | C | 0.05 | 0.773 ± 0.144 | 8.29E-08 |  | 0.05 | -0.213 ± 0.236 | 3.66E-01 |  | 0.505 | 3.91E-05 | 0.00 | 92.14 |
|  | Right eye tail length | rs970797 | 2 | 176,820,065 | *HOXD1*  *-MTX2* | A | C | 0.33 | 0.260 ± 0.056 | 3.87E-06 |  | 0.34 | 0.171 ± 0.094 | 6.83E-02 |  | 0.236 | 9.41E-07 | 0.42 | 0.00 |
|  |  | rs3736712 | 6 | 169,699,889 | *WDR27* | C | T | 0.37 | 0.322 ± 0.055 | **5.89E-09** |  | 0.38 | 0.208 ± 0.095 | 2.93E-02 |  | 0.293 | **8.44E-10** | 0.30 | 6.92 |
|  |  | rs8026249 | 15 | 69,727,672 | *THSD4* | T | C | 0.34 | 0.283 ± 0.056 | 3.82E-07 |  | 0.32 | 0.073 ± 0.096 | 4.46E-01 |  | 0.230 | 1.76E-06 | 0.06 | 72.18 |
| Height | Left palpebrale fissure height | rs12675712 | 8 | 121,672,671 | *SNTB1* | T | A | 0.16 | 0.174 ± 0.037 | 2.44E-06 |  | 0.16 | 0.068 ± 0.064 | 2.88E-01 |  | 0.147 | 3.92E-06 | 0.15 | 51.93 |
|  |  | rs17231256 | 21 | 39,370,759 | *ETS2-PSMG1* | T | C | 0.13 | -0.203 ± 0.042 | 1.12E-06 |  | 0.13 | -0.074 ± 0.071 | 2.98E-01 |  | -0.170 | 2.25E-06 | 0.11 | 59.78 |
| Ratio | Eye ratio of width to height | rs7144116 | 14 | 57,209,281 | *SLC35F4* | A | T | 0.41 | -0.004 ± 0.001 | 4.64E-06 |  | 0.40 | 0.002 ± 0.002 | 3.16E-01 |  | -0.003 | 5.65E-04 | 0.00 | 90.14 |
| Width | Intercanthal width | rs13404793 | 2 | 47,315,815 | *CALM2*  *-EPCAM* | T | C | 0.17 | -0.432 ± 0.091 | 2.07E-06 |  | 0.16 | -0.020 ± 0.154 | 8.97E-01 |  | -0.325 | 3.23E-05 | 0.02 | 81.17 |
|  |  | rs7127228 | 11 | 127,407,910 | intergenic | A | G | 0.29 | -0.384 ± 0.075 | 3.21E-07 |  | 0.29 | -0.070 ± 0.125 | 5.79E-01 |  | -0.301 | 2.96E-06 | 0.03 | 78.49 |
|  |  | rs1879682 | 18 | 67,648,221 | *LOC100505776* | A | T | 0.47 | 0.333 ± 0.069 | 1.29E-06 |  | 0.47 | 0.171 ± 0.113 | 1.29E-01 |  | 0.289 | 8.29E-07 | 0.22 | 33.82 |
|  | Outercanthal width | rs8026249 | 15 | 69,727,672 | *THSD4* | T | C | 0.34 | 0.568 ± 0.114 | 6.14E-07 |  | 0.32 | 0.375 ± 0.212 | 7.69E-02 |  | 0.525 | 1.66E-07 | 0.42 | 0.00 |
| ***Nose*** |  |  |  |  |  |  |  |  |  |  |  |  |  |  |  |  |  |  |  |
| Angle | Profile nasal angle | rs10744843 | 12 | 113,897,126 | *TBX3-MED13L* | A | G | 0.27 | -0.007 ± 0.001 | **1.54E-10** |  | 0.28 | 0.001 ± 0.002 | 6.82E-01 |  | -0.006 | **4.73E-08** | 0.00 | 91.31 |
|  |  | rs2159042 | 17 | 66,920,924 | *CASC17-SOX9* | C | T | 0.48 | -0.006 ± 0.001 | **5.22E-09** |  | 0.49 | -0.006 ± 0.002 | 1.14E-03 |  | -0.006 | **2.17E-11** | 0.98 | 0.00 |
|  |  | rs2024070 | 17 | 66,931,526 | *CASC17-SOX9* | C | T | 0.48 | -0.006 ± 0.001 | **3.00E-09** |  | 0.48 | -0.006 ± 0.002 | 2.90E-03 |  | -0.006 | **3.06E-11** | 0.80 | 0.00 |
|  |  | rs2193054 | 17 | 67,537,404 | *SOX9* | C | G | 0.47 | -0.007 ± 0.001 | **1.43E-11** |  | 0.46 | -0.009 ± 0.002 | 4.60E-07 |  | -0.007 | **6.17E-17** | 0.22 | 33.67 |
|  |  | rs2058742 | 17 | 67,552,142 | *SOX9* | T | G | 0.28 | 0.007 ± 0.001 | **6.41E-09** |  | 0.28 | 0.010 ± 0.002 | 9.32E-07 |  | 0.007 | **8.41E-14** | 0.13 | 57.43 |
|  | Nasal bridge angle | rs9327968 | 5 | 105,996,082 | *LOC102467213*  *-EFNA5* | C | A | 0.22 | -0.528 ± 0.110 | 1.74E-06 |  | 0.22 | -0.496 ± 0.191 | 9.40E-03 |  | -0.520 | 5.21E-08 | 0.88 | 0.00 |
|  |  | rs9395049 | 6 | 44,900,696 | *CDC5L*  *-SUPT3H* | T | C | 0.35 | 0.438 ± 0.095 | 4.20E-06 |  | 0.35 | 0.289 ± 0.164 | 7.85E-02 |  | 0.401 | 1.13E-06 | 0.43 | 0.00 |
|  |  | rs4787778 | 16 | 25,808,481 | *HS3ST4* | C | T | 0.26 | -0.490 ± 0.104 | 2.63E-06 |  | 0.26 | -0.280 ± 0.179 | 1.19E-01 |  | -0.437 | 1.24E-06 | 0.31 | 3.01 |
|  |  | rs6503196 | 17 | 9,242,511 | *STX8* | G | A | 0.15 | -0.601 ± 0.131 | 4.76E-06 |  | 0.15 | 0.280 ± 0.220 | 2.04E-01 |  | -0.370 | 1.03E-03 | 0.00 | 91.53 |
|  | Nasolabial angle | rs12666591 | 7 | 83,353,031 | *SEMA3E*  *-SEMA3A* | C | T | 0.08 | 0.021 ± 0.004 | 7.75E-07 |  | 0.09 | 0.002 ± 0.008 | 8.37E-01 |  | 0.017 | 8.08E-06 | 0.03 | 78.24 |
|  |  | rs3105176 | 8 | 100,581,074 | *VPS13B* | C | T | 0.42 | 0.014 ± 0.002 | **3.72E-08** |  | 0.42 | 0.008 ± 0.005 | 8.39E-02 |  | 0.013 | **1.34E-08** | 0.30 | 8.59 |
|  |  | rs11054145 | 12 | 11,043,467 | *PRH1-PRR4* | C | T | 0.24 | 0.013 ± 0.003 | 3.34E-06 |  | 0.24 | 0.001 ± 0.005 | 8.45E-01 |  | 0.011 | 2.62E-05 | 0.04 | 75.22 |
|  |  | rs2193054 | 17 | 67,537,404 | *SOX9* | C | G | 0.47 | -0.014 ± 0.002 | **1.56E-08** |  | 0.46 | -0.008 ± 0.005 | 7.49E-02 |  | -0.012 | **5.21E-09** | 0.28 | 12.91 |
|  |  | rs2058742 | 17 | 67,552,142 | *SOX9* | T | G | 0.28 | 0.013 ± 0.003 | 9.92E-07 |  | 0.28 | 0.012 ± 0.005 | 1.41E-02 |  | 0.013 | **4.30E-08** | 0.89 | 0.00 |
| Area | Profile nasal area | rs6445445 | 3 | 65,012,525 | *ADAMTS9*  *-MAGI1* | C | A | 0.43 | -0.017 ± 0.003 | 2.48E-07 |  | 0.44 | 0.001 ± 0.006 | 9.24E-01 |  | -0.013 | 1.03E-05 | 0.01 | 86.21 |
|  |  | rs7330390 | 13 | 26,274,974 | *GPR12-USP12* | C | T | 0.29 | -0.017 ± 0.004 | 1.68E-06 |  | 0.29 | -0.005 ± 0.006 | 4.09E-01 |  | -0.014 | 4.81E-06 | 0.10 | 63.67 |
|  |  | rs11078212 | 17 | 13,776,934 | *HS3ST3A1*  *-COX10* | C | T | 0.08 | 0.029 ± 0.006 | 1.50E-06 |  | 0.08 | -0.002 ± 0.011 | 8.87E-01 |  | 0.022 | 3.57E-05 | 0.01 | 83.70 |
|  |  | rs2159042 | 17 | 66,920,924 | *CASC17-SOX9* | C | T | 0.48 | 0.021 ± 0.003 | **1.47E-10** |  | 0.49 | 0.013 ± 0.006 | 2.90E-02 |  | 0.019 | **2.95E-11** | 0.18 | 43.50 |
|  |  | rs2024070 | 17 | 66,931,526 | *CASC17-SOX9* | C | T | 0.48 | 0.021 ± 0.003 | **1.29E-10** |  | 0.48 | 0.011 ± 0.006 | 4.98E-02 |  | 0.019 | **5.45E-11** | 0.13 | 57.06 |
|  |  | rs2058742 | 17 | 67,552,142 | *SOX9* | T | G | 0.28 | -0.017 ± 0.004 | 3.02E-06 |  | 0.28 | -0.010 ± 0.006 | 1.03E-01 |  | -0.015 | 1.13E-06 | 0.37 | 0.00 |
| Depth | Nasal bridge depth | rs10817791 | 9 | 117,351,060 | *DEC1*  *-LOC101928775* | C | T | 0.37 | 0.307 ± 0.065 | 2.28E-06 |  | 0.36 | 0.113 ± 0.115 | 3.23E-01 |  | 0.260 | 4.11E-06 | 0.14 | 53.79 |
|  |  | rs2159042 | 17 | 66,920,924 | *CASC17-SOX9* | C | T | 0.48 | 0.339 ± 0.064 | 9.54E-08 |  | 0.49 | 0.094 ± 0.108 | 3.85E-01 |  | 0.277 | 4.45E-07 | 0.05 | 73.73 |
|  |  | rs2024070 | 17 | 66,931,526 | *CASC17-SOX9* | C | T | 0.48 | 0.330 ± 0.064 | 2.18E-07 |  | 0.48 | 0.088 ± 0.109 | 4.16E-01 |  | 0.268 | 1.00E-06 | 0.05 | 72.87 |
|  | Nasal tip protrusion | rs12212993 | 6 | 49,189,507 | *MUT* | C | T | 0.10 | -0.026 ± 0.006 | 3.53E-06 |  | 0.09 | -0.010 ± 0.009 | 3.09E-01 |  | -0.022 | 6.34E-06 | 0.14 | 54.63 |
|  |  | rs7330390 | 13 | 26,274,974 | *GPR12-USP12* | C | T | 0.29 | -0.017 ± 0.004 | 3.90E-06 |  | 0.29 | -0.002 ± 0.006 | 6.84E-01 |  | -0.013 | 3.12E-05 | 0.04 | 76.04 |
|  |  | rs2159042 | 17 | 66,920,924 | *CASC17-SOX9* | C | T | 0.48 | 0.017 ± 0.003 | 6.87E-07 |  | 0.49 | 0.019 ± 0.005 | 5.08E-04 |  | 0.017 | **1.37E-09** | 0.72 | 0.00 |
|  |  | rs2024070 | 17 | 66,931,526 | *CASC17-SOX9* | C | T | 0.48 | 0.017 ± 0.003 | 3.61E-07 |  | 0.48 | 0.019 ± 0.005 | 5.93E-04 |  | 0.018 | **8.18E-10** | 0.78 | 0.00 |
|  |  | rs2193054 | 17 | 67,537,404 | *SOX9* | C | G | 0.47 | 0.019 ± 0.003 | **1.93E-08** |  | 0.46 | 0.011 ± 0.005 | 4.98E-02 |  | 0.017 | **5.34E-09** | 0.23 | 31.80 |
|  |  | rs2058742 | 17 | 67,552,142 | *SOX9* | T | G | 0.28 | -0.023 ± 0.004 | **9.77E-10** |  | 0.28 | -0.013 ± 0.006 | 3.12E-02 |  | -0.020 | **2.09E-10** | 0.18 | 43.16 |
| Height | Frontal nasal height | rs550668 | 6 | 42,417,123 | *TRERF1* | G | A | 0.36 | -0.318 ± 0.066 | 1.34E-06 |  | 0.36 | 0.037 ± 0.120 | 7.60E-01 |  | -0.236 | 4.20E-05 | 0.01 | 85.16 |
|  | Profile nasal length | rs318070 | 5 | 41,480,555 | *PLCXD3* | C | T | 0.26 | -0.008 ± 0.002 | 4.02E-06 |  | 0.27 | 0.000 ± 0.003 | 9.39E-01 |  | -0.006 | 4.42E-05 | 0.03 | 78.35 |
|  |  | rs6591148 | 11 | 105,425,885 | *MSANTD4*  *-KBTBD3* | T | C | 0.41 | 0.007 ± 0.001 | 3.75E-06 |  | 0.41 | -0.003 ± 0.003 | 2.33E-01 |  | 0.005 | 5.27E-04 | 0.00 | 90.77 |
|  | Nasal bridge height | rs12573823 | 10 | 127,630,157 | *FANK1* | A | G | 0.42 | -0.337 ± 0.073 | 4.09E-06 |  | 0.42 | 0.169 ± 0.129 | 1.92E-01 |  | -0.214 | 7.55E-04 | 0.00 | 91.39 |
|  |  | rs2092846 | 20 | 7,415,296 | *LOC101929312*  *-HAO1* | T | C | 0.31 | 0.366 ± 0.079 | 3.25E-06 |  | 0.31 | 0.254 ± 0.138 | 6.63E-02 |  | 0.339 | 7.12E-07 | 0.48 | 0.00 |
|  | Nasal tip height | rs6851773 | 4 | 117,606,691 | *MIR1973* | G | A | 0.09 | -0.022 ± 0.005 | 1.94E-06 |  | 0.09 | 0.013 ± 0.008 | 1.16E-01 |  | -0.014 | 5.97E-04 | 0.00 | 92.53 |
| Width | Subnasal width | rs12140446 | 1 | 94,137,147 | *GCLM* | T | A | 0.44 | -0.217 ± 0.047 | 4.30E-06 |  | 0.44 | -0.004 ± 0.087 | 9.67E-01 |  | -0.169 | 4.91E-05 | 0.03 | 78.63 |
|  |  | rs4315762 | 4 | 155,082,875 | *SFRP2-DCHS2* | G | C | 0.26 | -0.247 ± 0.054 | 4.84E-06 |  | 0.26 | -0.098 ± 0.101 | 3.32E-01 |  | -0.214 | 7.04E-06 | 0.19 | 41.00 |
|  |  | rs17123109 | 12 | 47,173,860 | *C12orf54* | C | T | 0.40 | 0.222 ± 0.048 | 3.82E-06 |  | 0.39 | -0.119 ± 0.089 | 1.83E-01 |  | 0.146 | 5.80E-04 | 0.00 | 91.17 |
|  |  | rs1429433 | 16 | 71,251,853 | *ZFHX3* | A | G | 0.29 | -0.269 ± 0.051 | 1.79E-07 |  | 0.28 | 0.041 ± 0.100 | 6.81E-01 |  | -0.204 | 8.43E-06 | 0.01 | 86.92 |
|  |  | rs2206437 | 20 | 37,426,155 | *DHX35* | A | T | 0.26 | -0.272 ± 0.054 | 4.75E-07 |  | 0.28 | -0.316 ± 0.095 | 8.67E-04 |  | -0.283 | **1.61E-09** | 0.69 | 0.00 |
| ***Mouth*** |  |  |  |  |  |  |  |  |  |  |  |  |  |  |  |  |  |  |  |
| Height | Left upper lip thickness | rs4969189 | 17 | 73,956,453 | *SOCS3-CYTH1* | G | A | 0.35 | -0.023 ± 0.004 | 3.90E-07 |  | 0.34 | 0.007 ± 0.007 | 3.45E-01 |  | -0.014 | 1.49E-04 | 0.00 | 91.87 |
|  | Right upper lip thickness | rs6656763 | 1 | 62,434,380 | *L1TD1* | G | A | 0.36 | -0.021 ± 0.004 | 1.21E-06 |  | 0.35 | -0.004 ± 0.007 | 5.74E-01 |  | -0.017 | 8.78E-06 | 0.04 | 76.09 |
|  |  | rs6712370 | 2 | 69,708,598 | *AAK1* | G | C | 0.46 | -0.020 ± 0.004 | 1.23E-06 |  | 0.46 | -0.002 ± 0.007 | 8.05E-01 |  | -0.015 | 1.68E-05 | 0.02 | 80.50 |
|  |  | rs3743299 | 15 | 92,825,595 | *MCTP2* | A | G | 0.09 | -0.036 ± 0.007 | 6.94E-07 |  | 0.08 | 0.015 ± 0.013 | 2.49E-01 |  | -0.024 | 1.96E-04 | 0.00 | 91.77 |
| ***Upper eyelid*** |  |  |  |  |  |  |  |  |  |  |  |  |  |  |  |  |  |  |  |
| Angle | Tangent line angle of el1 | rs310201 | 1 | 65,122,196 | *JAK1* | T | C | 0.44 | -0.024 ± 0.004 | 7.51E-08 |  | 0.44 | 0.000 ± 0.006 | 9.78E-01 |  | -0.015 | 1.78E-05 | 0.00 | 90.58 |
|  |  | rs749613 | 3 | 2,527,565 | *CNTN4* | C | T | 0.45 | -0.020 ± 0.004 | 4.14E-06 |  | 0.44 | 0.000 ± 0.006 | 9.33E-01 |  | -0.013 | 1.84E-04 | 0.01 | 86.24 |
|  | Tangent line angle of el2 | rs7761214 | 6 | 18,697,112 | *MIR548A1* | A | G | 0.23 | 0.012 ± 0.002 | 3.22E-06 |  | 0.23 | 0.004 ± 0.004 | 3.84E-01 |  | 0.010 | 7.64E-06 | 0.12 | 59.24 |
|  | Tangent line angle of el3 | rs970797 | 2 | 176,820,065 | *HOXD1*  *-MTX2* | A | C | 0.33 | 0.017 ± 0.003 | **4.90E-08** |  | 0.34 | 0.011 ± 0.005 | 2.96E-02 |  | 0.015 | **7.40E-09** | 0.29 | 12.52 |
|  |  | rs10750754 | 11 | 105,721,720 | *AASDHPPT*  *-GUCY1A2* | G | A | 0.36 | 0.014 ± 0.003 | 2.12E-06 |  | 0.37 | 0.004 ± 0.005 | 3.43E-01 |  | 0.011 | 6.22E-06 | 0.08 | 66.70 |
|  | Tangent line angle of el4 | rs970797 | 2 | 176,820,065 | *HOXD1*  *-MTX2* | A | C | 0.33 | 0.015 ± 0.003 | 9.12E-08 |  | 0.34 | 0.001 ± 0.004 | 8.87E-01 |  | 0.011 | 3.89E-06 | 0.01 | 86.34 |
|  |  | rs10821147 | 9 | 95,311,188 | *FAM120A* | A | G | 0.50 | 0.013 ± 0.003 | 9.99E-07 |  | 0.50 | 0.004 ± 0.004 | 3.90E-01 |  | 0.010 | 3.66E-06 | 0.07 | 69.63 |
|  |  | rs2000737 | 11 | 56,643,443 | *LOC101927120*  *-LRRC55* | C | A | 0.10 | 0.021 ± 0.004 | 1.69E-06 |  | 0.10 | 0.008 ± 0.007 | 2.78E-01 |  | 0.017 | 3.43E-06 | 0.11 | 61.29 |
|  |  | rs6517645 | 21 | 41,304,348 | *DSCAM-BACE2* | G | T | 0.12 | 0.018 ± 0.004 | 3.91E-06 |  | 0.11 | -0.003 ± 0.007 | 5.95E-01 |  | 0.013 | 2.44E-04 | 0.00 | 87.76 |
|  | Tangent line angle of el5 | rs1901455 | 2 | 106,039,568 | *NCK2-C2orf40* | A | T | 0.30 | -0.012 ± 0.003 | 4.18E-06 |  | 0.31 | -0.001 ± 0.005 | 7.73E-01 |  | -0.009 | 3.76E-05 | 0.04 | 76.92 |
|  | Tangent line angle of el6 | rs970797 | 2 | 176,820,065 | *HOXD1-MTX2* | A | C | 0.33 | -0.013 ± 0.003 | 2.21E-06 |  | 0.34 | -0.010 ± 0.004 | 1.78E-02 |  | -0.012 | 1.33E-07 | 0.63 | 0.00 |
|  | Tangent line angle of el7 | rs2737227 | 8 | 116,713,296 | *TRPS1* | C | T | 0.22 | -0.046 ± 0.010 | 3.64E-06 |  | 0.21 | 0.027 ± 0.015 | 6.26E-02 |  | -0.023 | 4.94E-03 | 0.00 | 94.13 |
|  | Tangent line angle of er1 | rs1357204 | 2 | 53,462,867 | *ASB3* | G | A | 0.48 | 0.018 ± 0.004 | 4.40E-06 |  | 0.47 | 0.001 ± 0.006 | 9.16E-01 |  | 0.013 | 1.15E-04 | 0.01 | 84.05 |
|  | Tangent line angle of er3 | rs17032657 | 1 | 8,519,329 | *RERE* | G | T | 0.33 | 0.014 ± 0.003 | 1.40E-06 |  | 0.33 | 0.008 ± 0.005 | 1.16E-01 |  | 0.013 | 7.46E-07 | 0.25 | 24.90 |
|  |  | rs970797 | 2 | 176,820,065 | *HOXD1*  *-MTX2* | A | C | 0.33 | 0.015 ± 0.003 | 4.51E-07 |  | 0.34 | 0.014 ± 0.005 | 2.57E-03 |  | 0.015 | **3.97E-09** | 0.93 | 0.00 |
|  |  | rs2252421 | 10 | 71,198,730 | *NEUROG3*  *-COL13A1* | G | A | 0.25 | -0.016 ± 0.003 | 1.01E-06 |  | 0.25 | -0.005 ± 0.005 | 3.87E-01 |  | -0.013 | 3.65E-06 | 0.07 | 69.44 |
|  |  | rs17836498 | 18 | 55,839,149 | *PMAIP1-MC4R* | C | T | 0.25 | 0.015 ± 0.003 | 4.59E-06 |  | 0.25 | 0.004 ± 0.005 | 4.16E-01 |  | 0.012 | 1.37E-05 | 0.10 | 63.94 |
|  | Tangent line angle of er4 | rs970797 | 2 | 176,820,065 | *HOXD1*  *-MTX2* | A | C | 0.33 | 0.014 ± 0.003 | 1.39E-07 |  | 0.34 | 0.006 ± 0.004 | 1.70E-01 |  | 0.012 | 1.66E-07 | 0.13 | 56.19 |
|  |  | rs750688 | 3 | 160,841,472 | *SCHIP1* | A | G | 0.18 | -0.016 ± 0.003 | 3.43E-07 |  | 0.17 | -0.001 ± 0.006 | 8.48E-01 |  | -0.013 | 5.94E-06 | 0.02 | 82.08 |
|  |  | rs8180203 | 4 | 55,503,175 | *KIT-KDR* | A | G | 0.36 | 0.012 ± 0.003 | 4.71E-06 |  | 0.37 | 0.000 ± 0.004 | 9.29E-01 |  | 0.009 | 6.42E-05 | 0.02 | 80.13 |
|  |  | rs8180522 | 5 | 20,274,979 | *CDH18* | C | G | 0.26 | 0.013 ± 0.003 | 2.09E-06 |  | 0.25 | 0.000 ± 0.005 | 9.51E-01 |  | 0.010 | 4.40E-05 | 0.02 | 82.95 |
|  |  | rs1608157 | 7 | 149,877,957 | *GIMAP7*  *-GIMAP4* | G | C | 0.49 | -0.012 ± 0.002 | 6.02E-07 |  | 0.48 | -0.003 ± 0.004 | 5.12E-01 |  | -0.010 | 3.37E-06 | 0.05 | 73.79 |
|  | Tangent line angle of er5 | rs10263442 | 7 | 9,758,100 | *PER4* | G | A | 0.39 | 0.011 ± 0.002 | 2.49E-06 |  | 0.38 | 0.001 ± 0.004 | 8.83E-01 |  | 0.009 | 2.84E-05 | 0.03 | 78.91 |
|  |  | rs734953 | 11 | 101,142,416 | *TRPC6*  *-ANGPTL5* | G | A | 0.30 | 0.012 ± 0.003 | 1.97E-06 |  | 0.31 | -0.011 ± 0.005 | 1.85E-02 |  | 0.007 | 2.34E-03 | 0.00 | 94.72 |
|  | Tangent line angle of er7 | rs8069687 | 17 | 59,236,755 | *DDX42* | C | T | 0.07 | 0.084 ± 0.016 | 1.60E-07 |  | 0.07 | -0.011 ± 0.023 | 6.34E-01 |  | 0.053 | 5.68E-05 | 0.00 | 91.35 |
| Curvature | Right eyelid maximal curvature | rs764134 | 17 | 10,302,402 | *MYH4* | C | G | 0.11 | 0.069 ± 0.014 | 8.83E-07 |  | 0.10 | -0.010 ± 0.027 | 7.14E-01 |  | 0.053 | 2.52E-05 | 0.01 | 84.88 |
| Ratio | Left eyelid peak position ratio | rs970797 | 2 | 176,820,065 | *HOXD1*  *-MTX2* | A | C | 0.33 | -0.007 ± 0.001 | 7.73E-07 |  | 0.34 | 0.000 ± 0.003 | 9.81E-01 |  | -0.005 | 1.53E-05 | 0.02 | 82.68 |
|  | Right eyelid peak position ratio | rs17032657 | 1 | 8,519,329 | *RERE* | G | T | 0.33 | -0.007 ± 0.001 | 1.03E-06 |  | 0.33 | -0.003 ± 0.003 | 3.00E-01 |  | -0.006 | 1.72E-06 | 0.15 | 52.86 |
|  |  | rs970797 | 2 | 176,820,065 | *HOXD1*  *-MTX2* | A | C | 0.33 | -0.007 ± 0.001 | 2.23E-07 |  | 0.34 | -0.004 ± 0.003 | 8.78E-02 |  | -0.007 | 8.51E-08 | 0.29 | 11.39 |
|  | Left eyelid slant | rs6476878 | 9 | 4,553,316 | *SLC1A1* | T | C | 0.24 | 0.008 ± 0.002 | 6.29E-07 |  | 0.25 | 0.000 ± 0.003 | 8.87E-01 |  | 0.006 | 2.13E-05 | 0.01 | 85.37 |
|  |  | rs9948162 | 18 | 6,543,040 | *L3MBTL4*  *-ARHGAP28* | G | A | 0.28 | 0.007 ± 0.001 | 6.60E-07 |  | 0.28 | -0.001 ± 0.003 | 7.24E-01 |  | 0.005 | 3.13E-05 | 0.01 | 86.80 |
|  | Left eyelid medial slant | rs4405545 | 16 | 80,415,673 | *PLCG2* | G | A | 0.09 | 0.022 ± 0.005 | 3.74E-06 |  | 0.08 | 0.012 ± 0.009 | 1.60E-01 |  | 0.020 | 2.32E-06 | 0.29 | 9.39 |
|  | Right eyelid medial slant | rs11209151 | 1 | 68,011,522 | *GNG12* | G | C | 0.11 | 0.020 ± 0.004 | 2.30E-06 |  | 0.10 | 0.009 ± 0.008 | 2.45E-01 |  | 0.017 | 2.70E-06 | 0.19 | 41.47 |
|  |  | rs7561038 | 2 | 207,945,095 | *KLF7*  *-CREB1* | T | C | 0.21 | -0.015 ± 0.003 | 3.97E-06 |  | 0.19 | 0.003 ± 0.006 | 6.09E-01 |  | -0.011 | 1.37E-04 | 0.01 | 85.83 |
|  |  | rs2869695 | 4 | 88,885,241 | *DMP1-IBSP* | G | A | 0.13 | -0.019 ± 0.004 | 1.04E-06 |  | 0.13 | 0.003 ± 0.007 | 6.48E-01 |  | -0.013 | 7.00E-05 | 0.00 | 87.98 |
| Width | Left eyelid width | rs952667 | 11 | 5,412,659 | *OR51B5* | C | T | 0.08 | -0.057 ± 0.012 | 4.00E-06 |  | 0.08 | -0.010 ± 0.020 | 6.19E-01 |  | -0.044 | 2.75E-05 | 0.05 | 74.86 |
|  |  | rs2159042 | 17 | 66,920,924 | *CASC17-SOX9* | C | T | 0.48 | -0.032 ± 0.007 | 1.51E-06 |  | 0.49 | 0.010 ± 0.011 | 3.57E-01 |  | -0.020 | 3.01E-04 | 0.00 | 90.88 |
|  |  | rs2024070 | 17 | 66,931,526 | *CASC17-SOX9* | C | T | 0.48 | -0.030 ± 0.007 | 4.61E-06 |  | 0.48 | 0.009 ± 0.011 | 4.07E-01 |  | -0.019 | 5.20E-04 | 0.00 | 89.66 |
|  | Left eyelid peak width | rs10750754 | 11 | 105,721,720 | *AASDHPPT*  *-GUCY1A2* | G | A | 0.36 | -0.022 ± 0.005 | 1.87E-06 |  | 0.37 | -0.002 ± 0.007 | 8.03E-01 |  | -0.016 | 2.77E-05 | 0.02 | 81.06 |
|  | Right eyelid width | rs2241241 | 2 | 65,822,288 | *SPRED2* | G | A | 0.29 | -0.034 ± 0.007 | 1.42E-06 |  | 0.30 | 0.007 ± 0.012 | 5.31E-01 |  | -0.023 | 1.47E-04 | 0.00 | 89.24 |
|  | Right eyelid peak width | rs4240275 | 4 | 126,272,497 | *FAT4* | A | G | 0.13 | -0.032 ± 0.007 | 1.68E-06 |  | 0.13 | -0.004 ± 0.011 | 7.08E-01 |  | -0.025 | 1.52E-05 | 0.04 | 77.31 |
|  |  | rs880245 | 6 | 150,209,091 | *LRP11* | G | A | 0.21 | 0.025 ± 0.005 | 2.62E-06 |  | 0.22 | -0.012 ± 0.009 | 1.93E-01 |  | 0.016 | 7.34E-04 | 0.00 | 91.95 |

^a^Positions according to NCBI Build 36. ^b^Genes are defined as the gene within the SNP locates or genes closest to the SNP within a ±400-kb window when the SNP dose not locate within a gene.

Bold and underlined text indicates genome-wide significant *P*-values (5 × 10^-8^). CHR, chromosome; AF, coded allele frequency; Q, *P*-value for Cochrane’s Q statistic; *I^2^*, heterogeneity index.
