# Supplementary material for: Identification of five novel genetic loci related to facial morphology by genome-wide association studies
Source: BMC Genomics. 2018 Jun 19;19:481. doi: 10.1186/s12864-018-4865-9 (PMC6008943; doi:10.1186/s12864-018-4865-9)
Supplement: Supplementary file 9 — Table S6. Phenotypic variance explained by five face-associated SNPs shown in Table 2 in the Phase 1 population (DOCX 15 kb) [file 12864_2018_4865_MOESM9_ESM.docx]

**Table S6.** Phenotypic variance explained by five face-associated SNPs shown in Table 2 in the Phase 1 population

| Facial trait | Phenotypic variance explained by SNPs (%) | | | | | | |
| --- | --- | --- | --- | --- | --- | --- | --- |
|  | Covariates | rs7567283 | rs970797 | rs3736712 | rs2193054 | rs2206437 | 5 SNPs |
| Right facial angle of en-ex-go | 12.53 | **0.37** | 0.00 | -0.03 | 0.11 | 0.12 | 0.55 |
| Tangent line angle of er3 | 3.70 | 0.19 | **0.51** | 0.04 | 0.01 | 0.08 | 0.87 |
| Tangent line angle of el3 | 3.53 | 0.23 | **0.45** | 0.10 | 0.00 | 0.02 | 0.86 |
| Eye tail length | 7.46 | 0.13 | 0.35 | **0.54** | 0.00 | 0.00 | 1.06 |
| Profile nasal angle | 12.54 | 0.10 | 0.00 | -0.02 | **0.74** | 0.00 | 0.79 |
| Nasal tip protrusion | 13.63 | 0.15 | 0.00 | 0.00 | **0.50** | 0.00 | 0.66 |
| Subnasal width | 22.85 | 0.00 | 0.00 | 0.02 | 0.00 | **0.35** | 0.52 |

Covariates: age, sex, BMI, and recruit region

Phenotypic variance explained by SNP(s) (%) is obtained by R^2^ fraction of the associated SNP(s) from linear regression model (R^2^ of SNP(s) – R^2^ of covariates).
