# Supplementary material for: Identification of five novel genetic loci related to facial morphology by genome-wide association studies
Source: BMC Genomics. 2018 Jun 19;19:481. doi: 10.1186/s12864-018-4865-9 (PMC6008943; doi:10.1186/s12864-018-4865-9)
Supplement: Supplementary file 10 — Table S7. Additional associated facial traits of five novel SNPs in the discovery GWAS (P < 0.0001). (DOCX 18 kb) [file 12864_2018_4865_MOESM10_ESM.docx]

**Table S7. Additional associated facial traits of five novel SNPs in the discovery GWAS (*P* < 0.0001)**

| **SNP** | **CHR** | **Position (bp)^a^** | **Gene^b^** | **Coded allele** | **Non-coded allele** | **Facial Traits** | |  | **beta ± se** | ***P*-value** |
| --- | --- | --- | --- | --- | --- | --- | --- | --- | --- | --- |
| rs7567283 | 2 | 19,595,772 | *OSR1-WDR35* | G | A | Face shape | Angle | Left facial angle of ps-ex-go | -0.504 ± 0.129 | 9.08E-05 |
|  |  |  |  |  |  |  |  | Left facial angle of en-ex-go | -0.525 ± 0.110 | 1.96E-06 |
|  |  |  |  |  |  |  |  | Right facial angle of en-ex-go | -0.572 ± 0.109 | 1.72E-07 |
|  |  |  |  |  |  |  | Ratio | Facial ratio of chin width to height | -0.015 ± 0.003 | 1.42E-05 |
|  |  |  |  |  |  | Nose | Height | Frontal nasal height | 0.295 ± 0.075 | 7.82E-05 |
| rs970797 | 2 | 176,820,065 | *HOXD1-MTX2* | A | C | Eye | Length | Eye tail length | 0.260 ± 0.056 | 3.87E-06 |
|  |  |  |  |  |  | Upper eyelid | Angle | Tangent line angle of el3 | 0.017 ± 0.003 | **4.90E-08** |
|  |  |  |  |  |  |  |  | Tangent line angle of el4 | 0.015 ± 0.003 | 9.12E-08 |
|  |  |  |  |  |  |  |  | Tangent line angle of el6 | -0.013 ± 0.003 | 2.21E-06 |
|  |  |  |  |  |  |  |  | Tangent line angle of el7 | -0.035 ± 0.009 | 5.66E-05 |
|  |  |  |  |  |  |  |  | Tangent line angle of er3 | 0.015 ± 0.003 | 4.51E-07 |
|  |  |  |  |  |  |  |  | Tangent line angle of er4 | 0.014 ± 0.003 | 1.39E-07 |
|  |  |  |  |  |  |  |  | Tangent line angle of er7 | -0.035 ± 0.008 | 2.83E-05 |
|  |  |  |  |  |  |  | Ratio | Left eyelid peak position ratio | -0.007 ± 0.001 | 7.73E-07 |
|  |  |  |  |  |  |  |  | Right eyelid peak position ratio | -0.007 ± 0.001 | 2.23E-07 |
|  |  |  |  |  |  |  | Width | Left eyelid peak width | -0.020 ± 0.005 | 3.65E-05 |
| rs3736712 | 6 | 169,699,889 | *WDR27* | C | T | Eye | Length | Eye tail length | 0.322 ± 0.055 | **5.89E-09** |
|  |  |  |  |  |  |  | Width | Outercanthal width | 0.453 ± 0.113 | 6.34E-05 |
| rs2193054 | 17 | 67,537,404 | *SOX9* | C | G | Nose | Angle | Profile nasal angle | -0.007 ± 0.001 | **1.43E-11** |
|  |  |  |  |  |  |  |  | Nasolabial angle | -0.014 ± 0.002 | **1.56E-08** |
|  |  |  |  |  |  |  | Depth | Nasal tip protrusion | 0.019 ± 0.003 | **1.93E-08** |
| rs2206437 | 20 | 37,426,155 | *DHX35* | A | T | Face shape | Height | Facial height | -0.397 ± 0.102 | 9.59E-05 |
|  |  |  |  |  |  | Nose | Width | Subnasal width | -0.272 ± 0.054 | 4.75E-07 |

^a^Positions according to NCBI Build 36. ^b^Genes are defined as the gene within the SNP locates or genes closest to the SNP within a ±400-kb window when the SNP dose not locate within a gene.

Bold and underlined text indicates genome-wide significant *P* values (5 × 10^-8^). CHR, chromosome.
