# Supplementary material for: Identification of five novel genetic loci related to facial morphology by genome-wide association studies
Source: BMC Genomics. 2018 Jun 19;19:481. doi: 10.1186/s12864-018-4865-9 (PMC6008943; doi:10.1186/s12864-018-4865-9)
Supplement: Supplementary file 11 — Figure S4. Multiple associations of five loci among the 85 facial traits in the discovery GWAS. (PDF 266 kb) [file 12864_2018_4865_MOESM11_ESM.pdf]

Figure S4: Multiple associations of five loci among the 85 facial traits in the discovery GWAS

Green color, $P < 10^{-4}$; blue color, $P < 10^{-5}$; red color, $P < 5 \times 10^{-8}$; ® , ratio of two lines (same number)

A. rs7567283 (*OSR1-WDR35*)

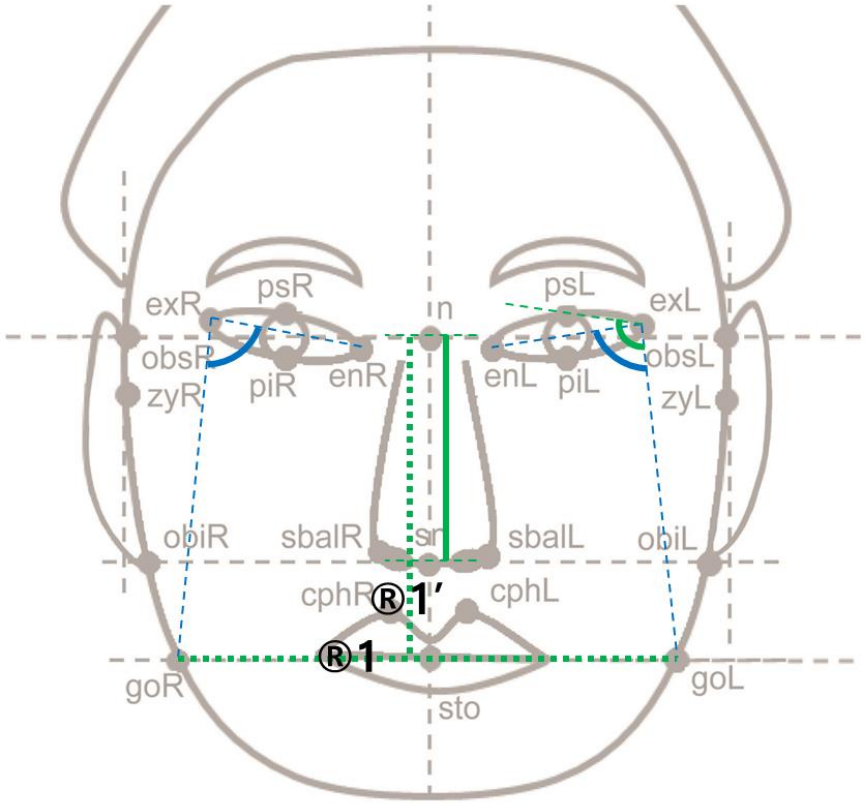

facial traits		Phenotype	BETA	P-value
Face shape	Angle	Left facial angle of ps-ex-go	-0.5043	9.08E-05
		Left facial angle of en-ex-go	-0.5252	1.96E-06
		Right facial angle of en-ex-go	-0.5719	1.72E-07
	Ratio	Facial ratio of chin width to height	-0.0148	1.42E-05
Nose	Height	Frontal nasal height	0.2947	7.82E-05

B. rs970797 (*HOXD1-MTX2*)

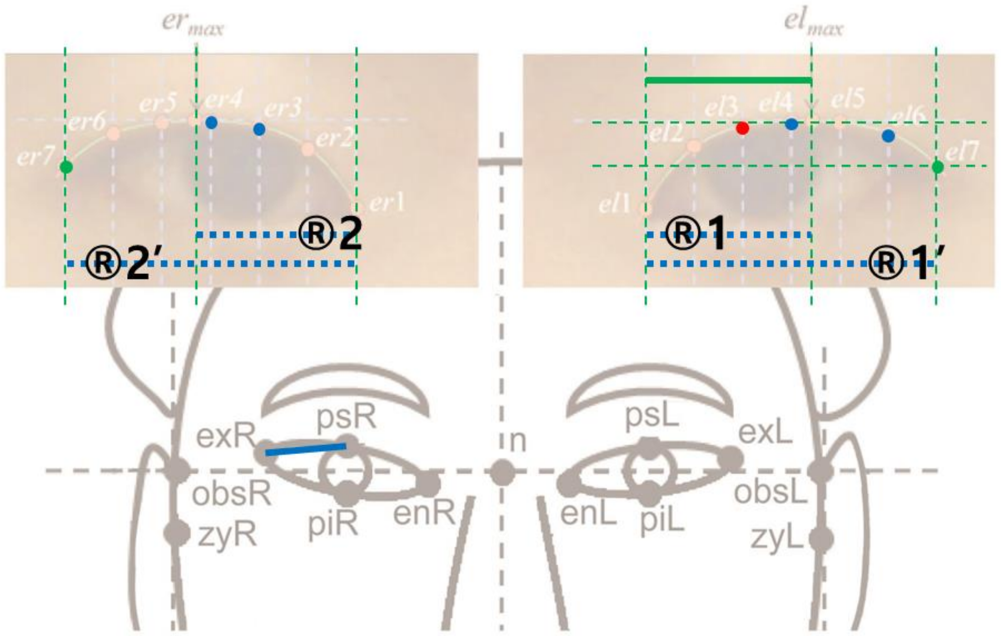

facial traits		Phenotype	BETA	P-value
Eye	Distance	Eye tail length	0.2595	3.87E-06
Upper eyelid	Angle	Tangent line angle of el3	0.0167	4.90E-08
		Tangent line angle of el4	0.0148	9.12E-08
		Tangent line angle of el6	-0.0126	2.21E-06
		Tangent line angle of el7	-0.0351	5.66E-05
		Tangent line angle of er3	0.0148	4.51E-07
		Tangent line angle of er4	0.0139	1.39E-07
		Tangent line angle of er7	-0.0353	2.83E-05
	Ratio	Left eyelid peak position ratio	-0.0071	7.73E-07
		Right eyelid peak position ratio	-0.0073	2.23E-07
	Width	Left eyelid peak width	-0.0196	3.65E-05

C. rs3736712 (WDR27)

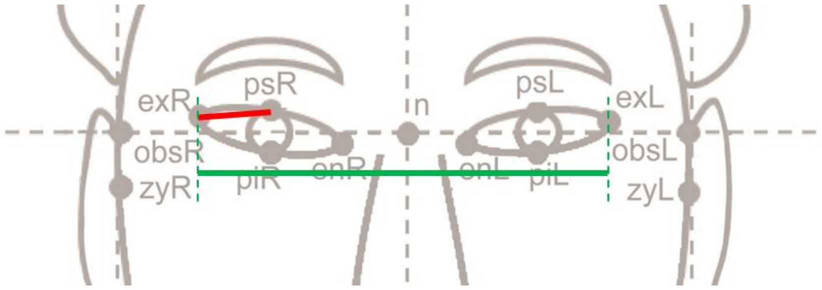

facial traits		Phenotype	BETA	P-value
Eye	Distance	Eye tail length	0.3216	5.89E-09
	Width	Outercanthal width	0.4527	6.34E-05

D. rs2193054 (*SOX9*)

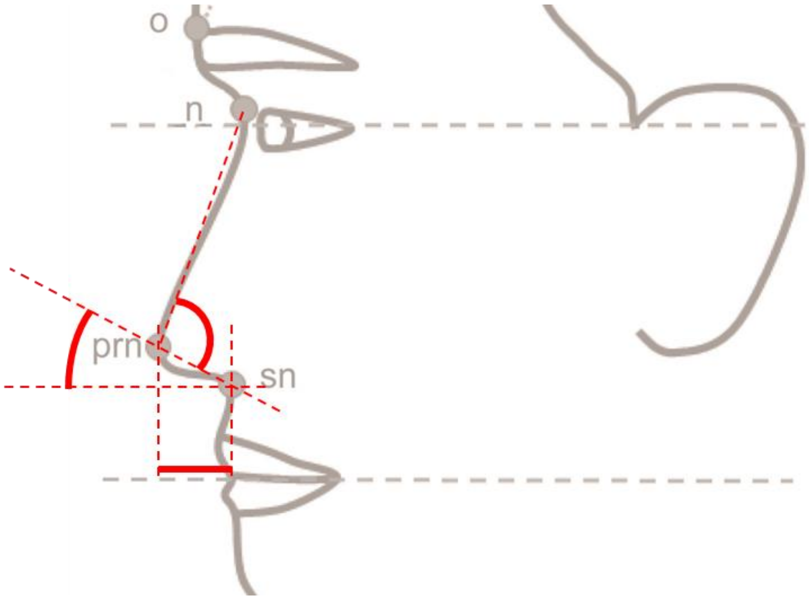

facial traits		Phenotype	BETA	P-value
Nose	Angle	Profile nasal angle	-0.0068	1.43E-11
		Nasolabial angle	-0.0137	1.56E-08
	Depth	Nasal tip protrusion	0.0185	1.93E-08

E. rs2206437 (*DHX35*)

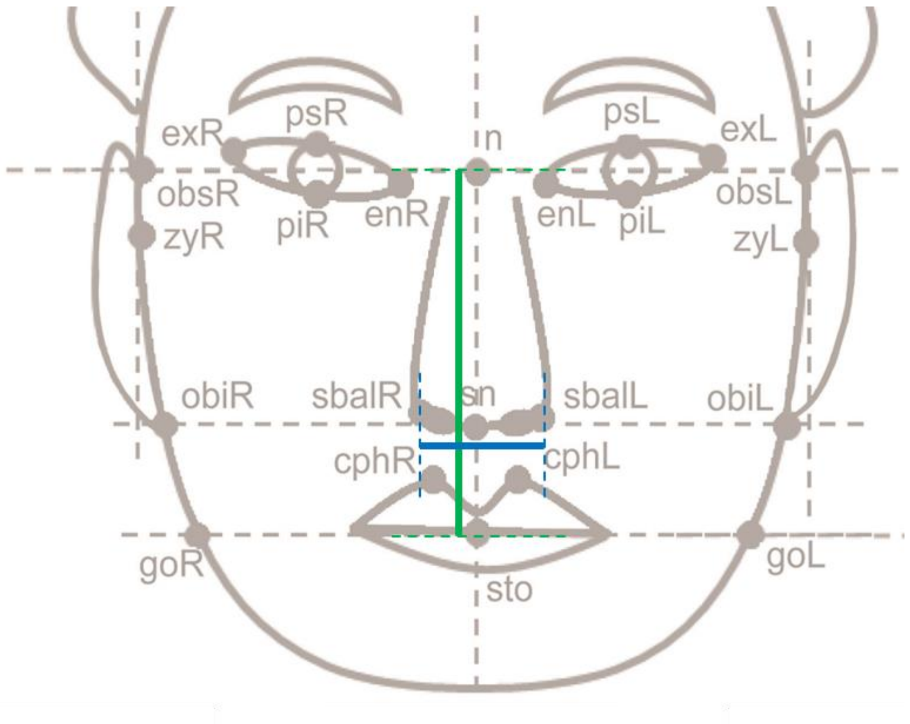

facial traits		Phenotype	BETA	P-value
Face shape	Height	Facial height	-0.3971	9.59E-05
Nose	Width	Subnasal width	-0.2724	4.75E-07
