# Supplementary material for: Identification of five novel genetic loci related to facial morphology by genome-wide association studies
Source: BMC Genomics. 2018 Jun 19;19:481. doi: 10.1186/s12864-018-4865-9 (PMC6008943; doi:10.1186/s12864-018-4865-9)
Supplement: Supplementary file 12 — Table S8. Pairwise linkage disequilibrium analyses with four nose-associated SNPs in the SOX9 locus. (DOCX 16 kb) [file 12864_2018_4865_MOESM12_ESM.docx]

**Table S8.** Pairwise linkage disequilibrium analyses with four nose-associated SNPs in the *SOX9* locus

| **SNP1** | **SNP2** | **LD (r^2^)** |  | **Population** | **Description** |
| --- | --- | --- | --- | --- | --- |
| **Studied population** | | | | | |
| rs9915190 | rs1859979 | 6.94E-05 | Korean | Phase 1 | Ansan and Ansung in Korea |
|  | rs9910003 | 1.96E-04 | Korean | Phase 1 | Ansan and Ansung in Korea |
|  | rs2193054 | 9.08 E-04 | Korean | Phase 1 | Ansan and Ansung in Korea |
| rs1859979 | rs9910003 | 6.16E-08 | Korean | Phase 1 | Ansan and Ansung in Korea |
|  | rs2193054 | 7.93E-05 | Korean | Phase 1 | Ansan and Ansung in Korea |
| rs9910003 | rs2193054 | 0.197 | Korean | Phase 1 | Ansan and Ansung in Korea |
| **1000 genomes population** | | | | | |
| rs9915190 | rs1859979 | 0.08 | African | 1000GENOMES:phase_3:ESN | Esan in Nigeria |
|  |  | 0.05 | American | 1000GENOMES:phase_3:PEL | Peruvian in Lima, Peru |
|  |  | 0.09 | American | 1000GENOMES:phase_3:PUR | Puerto Rican in Puerto Rico |
| rs9915190 | rs9910003 | 0.06 | African | 1000GENOMES:phase_3:ESN | Esan in Nigeria |
| rs9915190 | rs2193054 | 0.08 | African | 1000GENOMES:phase_3:ESN | Esan in Nigeria |
|  |  | 0.08 | African | 1000GENOMES:phase_3:ASW | African Ancestry in Southwest US |
| rs1859979 | rs9910003 | 0.06 | South Asian | 1000GENOMES:phase_3:GIH | Gujarati Indian in Houston, TX |
| rs9910003 | rs2193054 | 0.08 | African | 1000GENOMES:phase_3:ASW | African Ancestry in Southwest US |
|  |  | 0.10 | African | 1000GENOMES:phase_3:ESN | Esan in Nigeria |
|  |  | 0.06 | African | 1000GENOMES:phase_3:GWD | Gambian in Western Division |
|  |  | 0.06 | East Asian | 1000GENOMES:phase_3:CDX | Chinese Dai in Xishuangbanna, China |
|  |  | 0.10 | East Asian | 1000GENOMES:phase_3:CHB | Han Chinese in Bejing, China |
|  |  | 0.25 | East Asian | 1000GENOMES:phase_3:CHS | Southern Han Chinese, China |
|  |  | 0.12 | East Asian | 1000GENOMES:phase_3:JPT | Japanese in Tokyo, Japan |
|  |  | 0.09 | East Asian | 1000GENOMES:phase_3:KHV | Kinh in Ho Chi Minh City, Vietnam |
|  |  | 0.15 | South Asian | 1000GENOMES:phase_3:BEB | Bengali in Bangladesh |
|  |  | 0.14 | South Asian | 1000GENOMES:phase_3:GIH | Gujarati Indian in Houston, TX |
|  |  | 0.24 | South Asian | 1000GENOMES:phase_3:ITU | Indian Telugu in the UK |
|  |  | 0.20 | South Asian | 1000GENOMES:phase_3:STU | Sri Lankan Tamil in the UK |
|  |  | 0.08 | European | 1000GENOMES:phase_3:GBR | British in England and Scotland |
|  |  | 0.09 | European | 1000GENOMES:phase_3:IBS | Iberian populations in Spain |
|  |  | 0.13 | European | 1000GENOMES:phase_3:TSI | Toscani in Italy |
|  |  | 0.09 | American | 1000GENOMES:phase_3:MXL | Mexican Ancestry in Los Angeles |
