# Supplementary material for: Identification of five novel genetic loci related to facial morphology by genome-wide association studies
Source: BMC Genomics. 2018 Jun 19;19:481. doi: 10.1186/s12864-018-4865-9 (PMC6008943; doi:10.1186/s12864-018-4865-9)
Supplement: Supplementary file 13 — Table S9. Results of conditional analysis for four variants in the upstream region of SOX9 (Phase 1). (DOCX 34 kb) [file 12864_2018_4865_MOESM13_ESM.docx]

**Table S9.** Results of conditional analysis for four variants in the upstream region of *SOX9* (Phase 1)

| **SNP** | **CHR** | **Position (bp)^a^** | **Coded allele** | **Non-coded allele** | **Nose Traits** | **conditioning SNPs** | **beta ± se** | ***P*-value** |
| --- | --- | --- | --- | --- | --- | --- | --- | --- |
| rs9915190 | 17 | 66,654,223 | A | C | Nasal tip protrusion | None | -0.015 ± 0.003 | 6.03E-06 |
|  |  |  |  |  |  | rs1859979 | -0.015 ± 0.003 | 4.02E-06 |
|  |  |  |  |  |  | rs9910003 | -0.015 ± 0.003 | 7.45E-06 |
|  |  |  |  |  |  | rs2193054 | -0.016 ± 0.003 | 2.40E-06 |
|  |  |  |  |  |  | 3 SNPs | -0.016 ± 0.003 | 2.08E-06 |
|  |  |  |  |  | Nasal bridge depth | None | -0.198 ± 0.063 | 1.68E-03 |
|  |  |  |  |  |  | rs1859979 | -0.203 ± 0.063 | 1.31E-03 |
|  |  |  |  |  |  | rs9910003 | -0.191 ± 0.063 | 2.45E-03 |
|  |  |  |  |  |  | rs2193054 | -0.205 ± 0.063 | 1.20E-03 |
|  |  |  |  |  |  | 3 SNPs | -0.204 ± 0.063 | 1.22E-03 |
|  |  |  |  |  | Profile nasal area | None | -0.017 ± 0.003 | 3.10E-07 |
|  |  |  |  |  |  | rs1859979 | -0.017 ± 0.003 | 1.84E-07 |
|  |  |  |  |  |  | rs9910003 | -0.017 ± 0.003 | 3.91E-07 |
|  |  |  |  |  |  | rs2193054 | -0.017 ± 0.003 | 1.77E-07 |
|  |  |  |  |  |  | 3 SNPs | -0.017 ± 0.003 | 1.26E-07 |
|  |  |  |  |  | Nasolabial angle | None | 0.007 ± 0.002 | 3.21E-03 |
|  |  |  |  |  |  | rs1859979 | 0.007 ± 0.002 | 2.58E-03 |
|  |  |  |  |  |  | rs9910003 | 0.007 ± 0.002 | 3.84E-03 |
|  |  |  |  |  |  | rs2193054 | 0.008 ± 0.002 | 1.81E-03 |
|  |  |  |  |  |  | 3 SNPs | 0.008 ± 0.002 | 1.69E-03 |
|  |  |  |  |  | Profile nasal angle | None | 0.005 ± 0.001 | 5.79E-06 |
|  |  |  |  |  |  | rs1859979 | 0.005 ± 0.001 | 3.57E-06 |
|  |  |  |  |  |  | rs9910003 | 0.005 ± 0.001 | 6.28E-06 |
|  |  |  |  |  |  | rs2193054 | 0.005 ± 0.001 | 1.98E-06 |
|  |  |  |  |  |  | 3 SNPs | 0.005 ± 0.001 | 1.02E-06 |
| rs1859979 | 17 | 66,940,738 | C | T | Nasal tip protrusion | None | 0.018 ± 0.003 | 1.56E-07 |
|  |  |  |  |  |  | rs9915190 | 0.017 ± 0.003 | 4.61E-07 |
|  |  |  |  |  |  | rs9910003 | 0.018 ± 0.003 | 2.06E-07 |
|  |  |  |  |  |  | rs2193054 | 0.017 ± 0.003 | 2.19E-07 |
|  |  |  |  |  |  | 3 SNPs | 0.017 ± 0.003 | 7.50E-07 |
|  |  |  |  |  | Nasal bridge depth | None | 0.331 ± 0.064 | 2.21E-07 |
|  |  |  |  |  |  | rs9915190 | 0.329 ± 0.064 | 2.98E-07 |
|  |  |  |  |  |  | rs9910003 | 0.328 ± 0.064 | 3.00E-07 |
|  |  |  |  |  |  | rs2193054 | 0.329 ± 0.064 | 2.65E-07 |
|  |  |  |  |  |  | 3 SNPs | 0.323 ± 0.064 | 4.76E-07 |
|  |  |  |  |  | Profile nasal area | None | 0.022 ± 0.003 | **8.07E-11** |
|  |  |  |  |  |  | rs9915190 | 0.021 ± 0.003 | **8.64E-10** |
|  |  |  |  |  |  | rs9910003 | 0.022 ± 0.003 | **1.06E-10** |
|  |  |  |  |  |  | rs2193054 | 0.022 ± 0.003 | **1.01E-10** |
|  |  |  |  |  |  | 3 SNPs | 0.020 ± 0.003 | **1.39E-09** |
|  |  |  |  |  | Nasolabial angle | None | -0.011 ± 0.002 | 9.86E-06 |
|  |  |  |  |  |  | rs9915190 | -0.011 ± 0.002 | 1.72E-05 |
|  |  |  |  |  |  | rs9910003 | -0.011 ± 0.002 | 1.01E-05 |
|  |  |  |  |  |  | rs2193054 | -0.011 ± 0.002 | 1.29E-05 |
|  |  |  |  |  |  | 3 SNPs | -0.011 ± 0.002 | 2.10E-05 |
|  |  |  |  |  | Profile nasal angle | None | -0.006 ± 0.001 | **2.37E-09** |
|  |  |  |  |  |  | rs9915190 | -0.006 ± 0.001 | **3.19E-09** |
|  |  |  |  |  |  | rs9910003 | -0.006 ± 0.001 | **1.43E-09** |
|  |  |  |  |  |  | rs2193054 | -0.006 ± 0.001 | **3.70E-09** |
|  |  |  |  |  |  | 3 SNPs | -0.006 ± 0.001 | **2.53E-09** |
| rs9910003 | 17 | 67,390,279 | A | G | Nasal tip protrusion | None | 0.015 ± 0.004 | 4.05E-05 |
|  |  |  |  |  |  | rs9915190 | 0.015 ± 0.004 | 6.18E-05 |
|  |  |  |  |  |  | rs1859979 | 0.015 ± 0.004 | 4.68E-05 |
|  |  |  |  |  |  | rs2193054 | 0.007 ± 0.004 | 7.27E-02 |
|  |  |  |  |  |  | 3 SNPs | 0.007 ± 0.004 | 9.07E-02 |
|  |  |  |  |  | Nasal bridge depth | None | 0.038 ± 0.071 | 5.90E-01 |
|  |  |  |  |  |  | rs9915190 | 0.034 ± 0.071 | 6.37E-01 |
|  |  |  |  |  |  | rs1859979 | 0.034 ± 0.071 | 6.26E-01 |
|  |  |  |  |  |  | rs2193054 | -0.058 ± 0.079 | 4.63E-01 |
|  |  |  |  |  |  | 3 SNPs | -0.077 ± 0.079 | 3.33E-01 |
|  |  |  |  |  | Profile nasal area | None | 0.007 ± 0.004 | 7.71E-02 |
|  |  |  |  |  |  | rs9915190 | 0.006 ± 0.004 | 9.04E-02 |
|  |  |  |  |  |  | rs1859979 | 0.006 ± 0.004 | 8.37E-02 |
|  |  |  |  |  |  | rs2193054 | 0.002 ± 0.004 | 7.06E-01 |
|  |  |  |  |  |  | 3 SNPs | 0.001 ± 0.004 | 8.50E-01 |
|  |  |  |  |  | Nasolabial angle | None | -0.011 ± 0.003 | 1.12E-04 |
|  |  |  |  |  |  | rs9915190 | -0.010 ± 0.003 | 2.29E-04 |
|  |  |  |  |  |  | rs1859979 | -0.011 ± 0.003 | 1.25E-04 |
|  |  |  |  |  |  | rs2193054 | -0.005 ± 0.003 | 1.34E-01 |
|  |  |  |  |  |  | 3 SNPs | -0.004 ± 0.003 | 1.99E-01 |
|  |  |  |  |  | Profile nasal angle | None | -0.004 ± 0.001 | 3.87E-04 |
|  |  |  |  |  |  | rs9915190 | -0.004 ± 0.001 | 8.40E-04 |
|  |  |  |  |  |  | rs1859979 | -0.004 ± 0.001 | 4.65E-04 |
|  |  |  |  |  |  | rs2193054 | -0.001 ± 0.001 | 5.63E-01 |
|  |  |  |  |  |  | 3 SNPs | 0.0003 ± 0.001 | 8.06E-01 |
| rs2193054 | 17 | 67,537,404 | C | G | Nasal tip protrusion | None | 0.019 ± 0.003 | **1.93E-08** |
|  |  |  |  |  |  | rs9915190 | 0.019 ± 0.003 | **2.19E-08** |
|  |  |  |  |  |  | rs1859979 | 0.018 ± 0.003 | **2.70E-08** |
|  |  |  |  |  |  | rs9910003 | 0.015 ± 0.004 | 2.82E-05 |
|  |  |  |  |  |  | 3 SNPs | 0.015 ± 0.004 | 3.26E-05 |
|  |  |  |  |  | Nasal bridge depth | None | 0.165 ± 0.062 | 8.02E-03 |
|  |  |  |  |  |  | rs9915190 | 0.184 ± 0.063 | 3.34E-03 |
|  |  |  |  |  |  | rs1859979 | 0.161 ± 0.062 | 9.70E-03 |
|  |  |  |  |  |  | rs9910003 | 0.190 ± 0.070 | 6.41E-03 |
|  |  |  |  |  |  | 3 SNPs | 0.211 ± 0.070 | 2.50E-03 |
|  |  |  |  |  | Profile nasal area | None | 0.010 ± 0.003 | 1.33E-03 |
|  |  |  |  |  |  | rs9915190 | 0.011 ± 0.003 | 6.14E-04 |
|  |  |  |  |  |  | rs1859979 | 0.010 ± 0.003 | 1.69E-03 |
|  |  |  |  |  |  | rs9910003 | 0.010 ± 0.004 | 6.92E-03 |
|  |  |  |  |  |  | 3 SNPs | 0.011 ± 0.004 | 3.62E-03 |
|  |  |  |  |  | Nasolabial angle | None | -0.014 ± 0.002 | **1.56E-08** |
|  |  |  |  |  |  | rs9915190 | -0.014 ± 0.002 | **1.40E-08** |
|  |  |  |  |  |  | rs1859979 | -0.014 ± 0.002 | **2.04E-08** |
|  |  |  |  |  |  | rs9910003 | -0.012 ± 0.003 | 1.01E-05 |
|  |  |  |  |  |  | 3 SNPs | -0.012 ± 0.003 | 7.34E-06 |
|  |  |  |  |  | Profile nasal angle | None | -0.007 ± 0.001 | **1.43E-11** |
|  |  |  |  |  |  | rs9915190 | -0.007 ± 0.001 | **4.96E-12** |
|  |  |  |  |  |  | rs1859979 | -0.007 ± 0.001 | **2.22E-11** |
|  |  |  |  |  |  | rs9910003 | -0.007 ± 0.001 | **6.00E-09** |
|  |  |  |  |  |  | 3 SNPs | -0.007 ± 0.001 | **1.50E-09** |

^a^Positions according to NCBI Build 36.

Bold and underlined text indicates genome-wide significant *P*-values (5 × 10^-8^). CHR, chromosome.
