# Supplementary material for: Identification of five novel genetic loci related to facial morphology by genome-wide association studies
Source: BMC Genomics. 2018 Jun 19;19:481. doi: 10.1186/s12864-018-4865-9 (PMC6008943; doi:10.1186/s12864-018-4865-9)
Supplement: Supplementary file 14 — Table S10. Functional analysis of three variants in the upstream region of SOX9. (DOCX 15 kb) [file 12864_2018_4865_MOESM14_ESM.docx]

**Table S10.** Functional analysis of three variants in the upstream region of *SOX9*

| **SNP** | **CHR** | **Position (bp)^a^** | **Distance  from 5'  of *SOX9*** | **Coded allele** | **Non-coded allele** | **KOR** | **EAS** | **EUR** | **AFR** | **Promoter histone marks** | **Enhancer histone marks** | **DNAse** | **Motifs changed** | **eQTL** | **RegulomeDB score^b^** |
| --- | --- | --- | --- | --- | --- | --- | --- | --- | --- | --- | --- | --- | --- | --- | --- |
| rs9915190 | 17 | 66,654,223 | 974kb | A | C | 0.45 | 0.46 | 0.36 | 0.17 |  | ES-deriv, Breast, Adipose,  Muscle, Gastrointestine,  Pancreatic Islets |  | CTCF, Tgif1 |  | - |
| rs1859979 | 17 | 66,940,738 | 688kb | C | T | 0.46 | 0.44 | 0.95 | 0.90 | Muscle, Skin, Breast | ES-deriv, Adipose, Muscle,  Skin, Breast, Pancreatic Islets,  Lung, Cervical, Bone | Skin,  Breast | AFP1 |  | 5 |
| rs2193054 | 17 | 67,537,404 | 91kb | C | G | 0.47 | 0.45 | 0.50 | 0.24 | ESC, iPSC, ES-deriv, Blood, Stromal, Brain, Thymus,  Adipose, Muscle, Heart, Gastrointestine, Lung, Ovary,  Liver, Breast, Skin, Bone | ESC, iPSC, ES-deriv, Blood,  Stromal, Adipose, Muscle, Skin,  Breast, Brain, Thymus, Heart, Gastrointestine, Kidney, Lung,  Ovary, Pancreatic Islets, Spleen, Liver, Bone | Cervical | STAT | *SOX9* (Brain) | 6 |

^a^Positions according to NCBI Build 36. ^b^RegulomeDB score - 5, minimal binding evidence (TF binding or DNase peak); 6, minimal biding evidence; -, no data. CHR, chromosome; KOR, Korean (coded allele frequency); EAS, East Asian (coded allele frequency); EUR, European (coded allele frequency); AFR, African (coded allele frequency).
