# Supplementary material for: Identification of five novel genetic loci related to facial morphology by genome-wide association studies
Source: BMC Genomics. 2018 Jun 19;19:481. doi: 10.1186/s12864-018-4865-9 (PMC6008943; doi:10.1186/s12864-018-4865-9)
Supplement: Supplementary file 15 — Table S11. Comparison of clinical phenotypes and facial traits. (DOCX 14 kb) [file 12864_2018_4865_MOESM15_ESM.docx]

**Table S11. Comparison of clinical phenotypes and facial traits**

| **Gene** | **Known craniofacial malformation** | **Clinical phenotypes related to facial shape** | **Related-associated phenotypes** | **Associated SNP** |
| --- | --- | --- | --- | --- |
| *SOX9* | Campomelic dysplasia (CMPD) | Flat face |  | rs2193054 |
|  |  | Depressed nasal bridge | Profile nasal angle, nasal tip protrusion |  |
|  | Pierre robin sequence (PRS) | Small mandible |  |  |
| *WDR35* | Cranioectodermal dysplasia 2 (CED2) | Forehead bossing |  | rs7567283 |
|  |  | narrow or long face (Dolicholcephaly) | Right facial angle of en-ex-go (related to facial width) |  |
|  |  | Mandibular prognathism (Metaphyseal dysplasia) |  |  |
