# Supplementary material for: Identification of five novel genetic loci related to facial morphology by genome-wide association studies
Source: BMC Genomics. 2018 Jun 19;19:481. doi: 10.1186/s12864-018-4865-9 (PMC6008943; doi:10.1186/s12864-018-4865-9)
Supplement: Supplementary file 16 — Table S12. Comparisons of allele frequencies between Koreans and other populations from the 1000 Genomes Project Phase 3. (DOCX 16 kb) [file 12864_2018_4865_MOESM16_ESM.docx]

**Table S12. Comparisons of allele frequencies between Koreans and other populations from the 1000 Genomes Project Phase 3**

| **CHR** | **SNP** | **Gene**^a^ | **Allele**  **(Ref/Alt)** | **Minor allele**  **in Korean** | **Facial effect**  **(effect direction)** | **Minor allele frequency**  **(fold-difference)** | | | |
| --- | --- | --- | --- | --- | --- | --- | --- | --- | --- |
|  |  |  |  |  |  | **Korean** | **EAS** | **EUR** | **AFR** |
| 2 | rs7567283 | *OSR1-WDR35* | G/A | G | Facial angle of eye-chin (-) | 0.24 | 0.25 | 0.82  (3.4) | 0.67  (2.8) |
| 2 | rs970797 | *HOXD1-MTX2* | A/C | A | Eyelid peak position ratio (-) | 0.33 | 0.31 | 0.43  (1.3) | 0.4  (1.2) |
| 6 | rs3736712 | *WDR27* | C/T | C | Eye tail length (+) | 0.37 | 0.39 | 0.93  (2.5) | 0.93  (2.5) |
| 17 | rs9915190 | *SOX9* | C/A | A | Profile nasal area (-) | 0.45 | 0.46 | 0.36  (1.3) | 0.17  (2.6) |
| 17 | rs1859979 | *SOX9* | T/C | C | Profile nasal area (+) | 0.46 | 0.44 | 0.95  (2.1) | 0.90  (2.0) |
| 17 | rs9910003 | *SOX9* | A/G | A | Nasal tip protrusion (+) | 0.28 | 0.30 | 0.46  (1.6) | 0.57  (2.0) |
| 17 | rs2193054 | *SOX9* | C/G | C | Profile nasal size (+) | 0.47 | 0.45 | 0.5  (1.1) | 0.24  (2.0) |
| 20 | rs2206437 | *DHX35* | T/A | A | Nasal width (-) | 0.26 | 0.23 | 0.48  (1.8) | 0.25  (1.0) |

^a^Genes are defined as the gene within the SNP locates or genes closest to the SNP within a ±400-kb window when the SNP dose not locate within a gene.

CHR, chromosome; Ref, reference allele; Alt, alternate allele; EAS, East Asian; EUR, European; AFR, African.
