# Supplementary material for: Identification of five novel genetic loci related to facial morphology by genome-wide association studies
Source: BMC Genomics. 2018 Jun 19;19:481. doi: 10.1186/s12864-018-4865-9 (PMC6008943; doi:10.1186/s12864-018-4865-9)
Supplement: Supplementary file 17 — Figure S5. The procedure for facial feature point extraction. (PDF 362 kb) [file 12864_2018_4865_MOESM17_ESM.pdf]

Figure S5: The procedure for facial feature point extraction

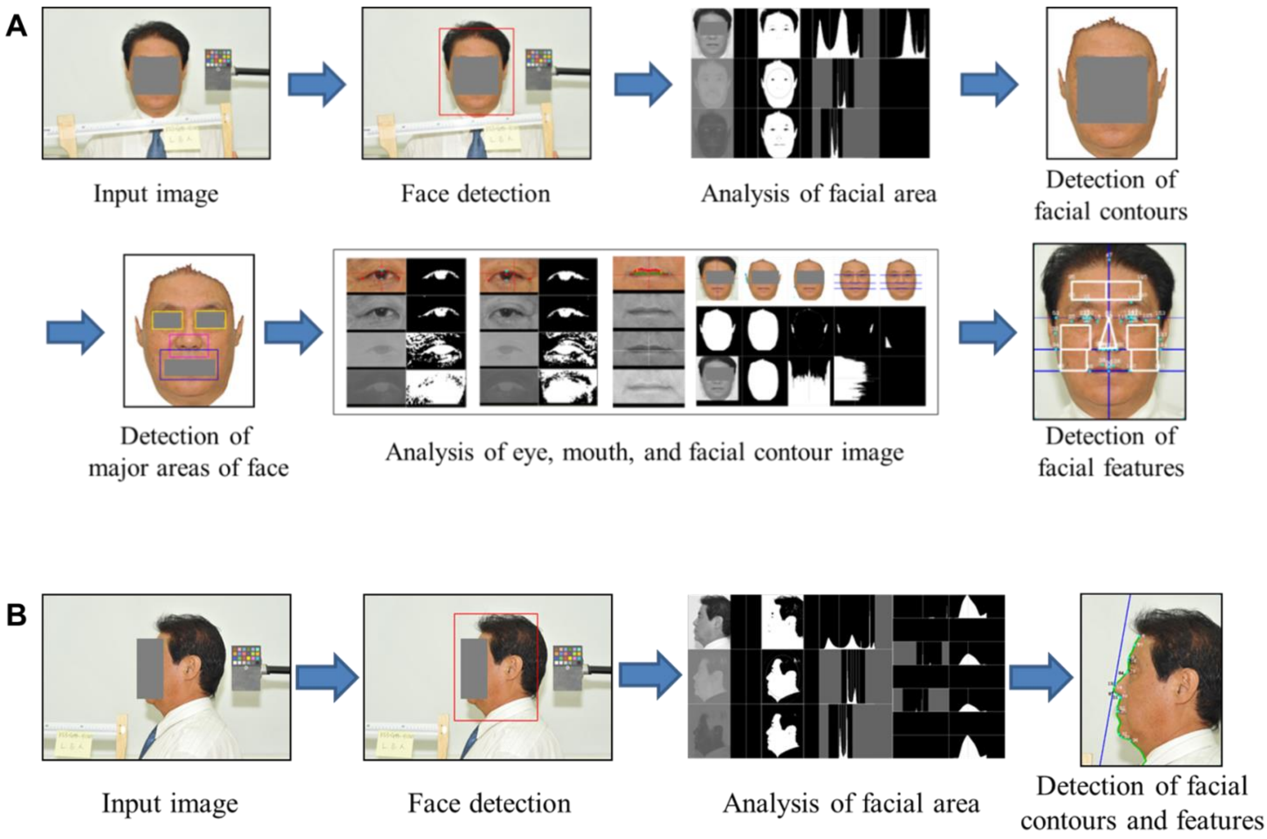

The participants were photographed with a neutral expression in both the frontal and profile views, and 23 frontal and seven lateral face points were extracted. Distance, angle, and area measurements were carried out based on in-house facial data acquisition software [25, 29]. (**A**) frontal image extraction, (**B**) lateral image extraction.
